# New Small Nuclear RNA Gene-Like Transcriptional Units as Sources of Regulatory Transcripts

**DOI:** 10.1371/journal.pgen.0030001

**Published:** 2007-02-02

**Authors:** Aldo Pagano, Manuele Castelnuovo, Federico Tortelli, Roberto Ferrari, Giorgio Dieci, Ranieri Cancedda

**Affiliations:** 1 Dipartimento di Oncologia Biologia e Genetica, Università di Genova, Genoa, Italy; 2 Istituto Nazionale per la Ricerca sul Cancro, Genoa, Italy; 3 Dipartimento di Biochimica e Biologia Molecolare, Università di Parma, Parma, Italy; Lawrence Livermore National Laboratory, United States of America

## Abstract

By means of a computer search for upstream promoter elements (distal sequence element and proximal sequence element) typical of small nuclear RNA genes, we have identified in the human genome a number of previously unrecognized, putative transcription units whose predicted products are novel noncoding RNAs with homology to protein-coding genes. By elucidating the function of one of them, we provide evidence for the existence of a sense/antisense-based gene-regulation network where part of the polymerase III transcriptome could control its polymerase II counterpart.

## Author Summary

After the sequence of the human genome was determined, it was immediately recognized that a large part of the regulation of the gene expression occurring in the cells under physiological, as well as under pathological conditions, is carried out by RNA molecules that do not code for proteins (the “noncoding portion” of the genome). Here, we focus on small RNA molecules transcribed by the RNA polymerase III and identify a novel set of approximately 30 noncoding (nc) RNA genes. We propose that these RNA transcripts play a key role in regulating the expression of specific protein-coding genes transcribed by the RNA polymerase II, thus constituting an unprecedented example of cogene/gene pairs. Furthermore, we provide evidence that the RNA polymerase III, in addition to the well-known task in the constitutive synthesis of small RNAs (such as *5S* rRNA and tRNAs), also plays a key role in the area of gene-expression control. A detailed investigation of the function of one of the novel ncRNA genes, called *21A,* revealed that its transcript plays a role in the control of the proliferation of some tumor cells. The above findings significantly expand our understanding of the ncRNA universe and open the way to further studies aimed at the elucidation of the molecular pathways involving this novel class of regulatory RNAs.

## Introduction

Recent advances in mammalian genome studies are bringing to light the occurrence of a widespread transcription of noncoding (nc) regions devoted to the regulation of the protein coding genome expression [1–4]. The mechanisms of action of these transcripts are various and of different natures, although all of them are devoted to the regulation of fundamental genetic pathways involved in the determination of the cell phenotype. The concomitant evolution of noncoding regulatory transcripts and proteins that target different RNA:RNA or RNA:DNA complexes emphasizes the importance of studying the regulatory processes mediated by nucleic acid interactions. It has been demonstrated that in both prokaryotes and eukaryotes, *cis*-acting RNA regulatory regions (i.e., 3′ UTRs forming secondary structures with regulatory features or containing sequences recognized by proteins involved in RNA stability modulation) can be simultaneously regulated in *trans* by other noncoding RNAs (i.e., microRNAs) or by protein complexes. The simultaneous occurrence of *cis*- and *trans*-regulatory elements brings to light the complexity of this network where the coexistence of different ncRNAs plays a key role in the control of other target gene expression [5]. In this context a prominent role is played by the enlarging family of microRNAs (miRNAs) that act at a posttranscriptional level by inhibiting the translation of protein-coding genes [6]. The known miRNAs, similar to protein-coding mRNAs, are synthesized as polyadenylated precursor molecules by the RNA polymerase (pol) II transcription machinery [7]. The vast majority of the tools used in molecular biology are based on transcript collections obtained by oligo (dT) reverse transcriptase (RT)-PCR, thus encompassing only polyadenylated pol II products. However, a wide contribution of nonpolyadenylated transcripts to the human transcriptome has been revealed [8]. The role of such transcripts in pol II transcriptome–expression regulation remains largely unexplored.

Among the noncoding elements, one of the most investigated has been the *Alu* class of repetitive sequences that represents about one-tenth of the whole human genome. Although it is not yet possible to discern a peculiar role of *Alu* sequences, their short transcripts have been shown to be involved in several biological processes such as: RNA editing (where *Alus* are preferential sites for A to I RNA editing thus having profound implications both in gene-expression regulation and in mammalian genome evolution) [9], alternative splicing (internal exons that contain an *Alu* sequence are almost always alternatively spliced) [10], chromosomal recombination (the recombination between *Alu* elements is at the base of genomic deletions associated with many human genetic disorders) [11], gene-expression regulation (functioning as naturally occurring antisense RNAs) [12], cell-stress response (such as heat-shock response and/or translation inhibition) [13], and as putative miRNAs targets [14]. The physiological role of *Alus* and all the other *7SL*-derived transcripts needs to be studied in more detail. In particular, the fact that their transcription is RNA pol III–dependent brings to light a previously unexpected role in gene-expression regulation of this enzyme that deserves investigation.

In this study, starting from the observation that pol III is specialized in transcription of ncRNA genes, we addressed the hypothesis that the human genome might contain pol III transcription units each specifically regulating one (or more) specific pol II genes, thus constituting functional “cogene”/gene pairs.

## Results/Discussion

### In Silico Identification of a Novel Set of Small Nuclear RNA Gene-Like Transcriptional Units in the Human Genome

To test our hypothesis we focused on pol III type 3–extragenic promoters, which are located upstream of the transcribed region. We screened the human genome for regions containing the consensus sequences characteristic of pol III type 3 promoters: the proximal sequence element (PSE) and the distal sequence element (DSE) [15,16]. First, we tested the PSE sequences of three well-characterized pol III type 3–ncRNA genes *(U6, H1,* and *7SK)* as query sequences for the search of similar (if not equal) elements in the human genome by using the BLAST algorithm (http://www.ncbi.nlm.nih.gov/BLAST; under “Nucleotide” subsection select “short, nearly exact matches” option, then pull down *“Homo sapiens”* organism database) (see Materials and Methods for sequences used as query). While the search with *U6* and *7SK* PSE sequences did not identify a significant number of homologous regions scattered throughout the genome, the *H1* PSE element shared a high homology with 60 novel putative PSE sequences. Among these we selected (by a BLAST analysis) those that contained a DSE sequence element within an arbitrarily defined distance of 1,000 basepairs upstream the PSE. In addition to the expected *H1,* results evidenced 31 novel putative PSE/DSE-dependent promoters characterized by the concomitant occurrence of the PSE and the DSE sequences within that defined genomic distance. Moreover, a detailed sequence analysis showed that the vast majority of the distances between the PSE and a downstream TATA box or TATA-like element are within 18–22 basepairs, as expected for a canonical type 3 promoter [16]. Altogether these observations were taken as preliminary indication of a functional pol III type 3 structure of these novel promoters (Table S1).

Since our search was based on pol III type 3 promoters, some additional features of these promoters needed to be considered: (i) the occurrence of a PSE consensus sequence does not identify per se a pol III type 3 promoter; that is, rather, the result of the simultaneous occurrence at an appropriate distance of the PSE and an A/T-rich (TATA-like) element. Indeed, it has been clearly shown that the occurrence of a PSE consensus that lacks a downstream A/T-rich element makes the promoter readable by RNA pol II, such as in the case of the *U2* snRNA gene [17]. In this context, the transcription initiation region is not relevant for the choice of the RNA polymerase, at least in humans, although it seems to be of fundamental importance in Xenopus [18]. Therefore, the PSE/DSE-flanked, putative transcription units identified by our search might in theory be transcribed either by pol II or by pol III, depending on the occurrence of a functional A/T-rich region downstream of the PSE. (ii) Pol III transcription units are characterized by the presence of very simple and easily recognizable termination signals, consisting in a run of four or more consecutive T residues. We thus searched, within the collection of DSE/PSE-containing sequences, for the further occurrence of a TATA-like element downstream of the PSE and for the occurrence of a termination signal at a significant distance downstream of the hypothetical transcription start site, assumed to be located approximately 30 basepairs downstream of the TATA element. Such a search refinement revealed that most of the newly identified sequences have features compatible with a pol III type 3 promoter structure (Table S2).

Our in silico search was based on the *H1* PSE sequence, which was used as a query only allowing for mismatches in the first and last positions. The search thus likely identified only those promoters whose structure is very similar to that of *H1*. This is supported by the fact that out of *H1* no other previously known pol III type 3 promoters were found in our search. Given the divergence among the PSE sequences of *U6, 7SK,* and *H1,* it is to be expected that the use, as a query sequence, of a more degenerated PSE consensus, derived from the known pol III type 3 PSE sequences, would bring to light a considerably higher number of putative PSE-dependent transcription units in the human genome.

To further characterize in silico the novel transcription units, we arbitrarily assumed as transcribed the region starting from the 30th nucleotide downstream of the first nucleotide of the predicted TATA box. In addition, a run of at least four T residues was considered as a pol III transcription–termination signal, although events of “read-through” are possible at T_4_ sequences depending on sequence context features [19,20]. While it has to be emphasized that the transcribed region of each element of this collection needs to be experimentally determined, we selected 32 putative novel transcripts to be subjected to additional analysis on the basis of their in silico characterization.

To test if a common secondary structure could be a hallmark of the novel molecules, an in silico analysis of their secondary structure was performed by mfold algorithm (http://www.bioinfo.rpi.edu/applications/mfold/rna/form1.cgi) [21]. Results showed that although hairpins with short stems (5–7 basepairs) were frequent, no shared secondary structures were recurrent, indicating that a peculiar molecular organization is not the common hallmark of this set of noncoding molecules. Although their averaged free energy (ΔG) was extremely variable (−42.7 ± 41.2), a group of four transcripts *(11A, 20A, 21A,* and *29A)* showed a ΔG value significantly lower than all the others (ΔG < −100). A statistical analysis of such ΔG differences was performed evidencing that the differences between this group of transcripts *(11A, 20A, 21A,* and *29A),* and the rest of the pool is highly significant (Student's *t*-test, 33 degrees of freedom, α significance level = 0.1 corresponding to a *p-*value of 0.0001), thus keeping in line with their physiologically functional–secondary structure organization (Table S3).

In order to assess if the pool of transcription units was prevalently constituted by repeats such as retroposons, we analyzed their sequences by Repeat Masker algorithm (http://repeatmasker.org) evidencing that: (i) only two out of 32 (6.2%) are short interspersed nucleotide elements (*21A* and *29A,* which were marked as *AluJb* elements); (ii) three of them *(24A, 37A,* and *38A)* are part of long interspersed nucleotide elements; (iii) two sequences *(17A* and *40A)* contained a mammalian interspersed repeat (MIR), and (iv) three sequences *(30A, 32A,* and *44A)* contained different types of long terminal repeats (Table S4). Considering that Alus, long interspersed nucleotide elements, and MIRs constitute about 15%, 30%, and 1%–5% of the human genome, respectively, one would have expected a higher frequency of repeats in the pool of sequences. Altogether these observations provide evidence that the novel PSE-dependent transcripts are not associated to a specific class of repetitive sequences scattered throughout the human genome, but instead they constitute a novel heterogeneous set of type 3 promoter-driven elements.

When these noncoding sequences were used to challenge the human genome database (BLAST analysis), it was found that seven of them were internal to known or predicted protein-coding genes, four being in antisense and three in sense configuration. Most of the novel sequence elements not mapping in coding regions shared a high sequence homology (∼80%) to a pol II transcript/expressed sequence tag that maps in a different locus (Table S5). Such homologies reached even higher values (up to 90%), if only parts of the putative transcripts were considered. In fact, no expressed sequence tags entirely containing one of our transcription units were found, so that if a sense/antisense-based regulation would occur, it would likely be related to parts of the ncRNA sequences, while the other part could have structural properties that facilitate this regulatory action (perhaps by binding specific structural proteins). Based on these observations, a novel control mechanism of gene expression could be postulated where pol III (or pol III-like) elements act as *trans*-locus antisense of their homologous protein-coding RNAs. In this model, the pol III cogenes in antisense configuration with respect to one (or more) specific target gene(s) could regulate their expression either by interfering with its mRNA maturation (if the homologous region is internal to an intron) or by inhibiting protein translation (if the homology is associated to an exon).

### *21A* as Cogene Experimental Model

**Figure 1:**
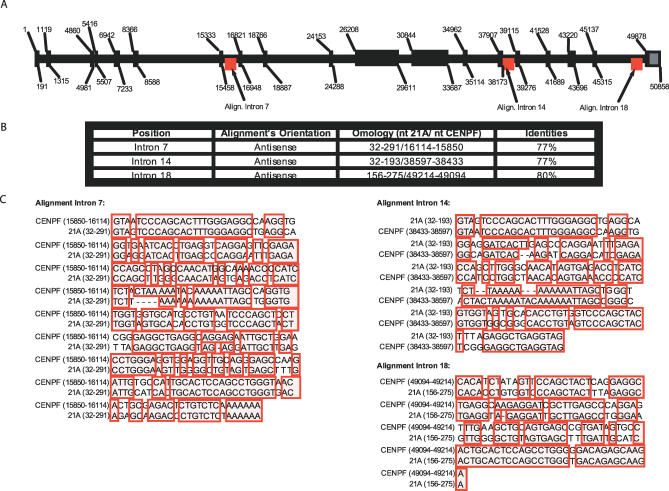
*CENP-F* as *21A*-Specific Molecular Target (A) Human *CENP-F* gene structure as resulting from GI:89161185 (region 212843155 - 212904537). (B) The positions of the *21A* antisense homologous regions are reported together with their percentage of identity. (C) Sequence alignment of *21A/CENP-F* homologous regions.

To test our hypothesis we selected one of the novel transcription units (here referred to as *21A*) that maps in 8q24.13. If aligned to the human genome, it shows several homology hits among which the most highly significant were associated to multiple intronic regions of centromeric protein F gene (*CENP-F;* 1q32-q41) [22], thus constituting its putative natural *trans*-chromosomal antisense (Figure 1A–1C). Although, similarly to all of the *7SL/Alu*-derived elements, *21A* is expected to be primate-specific [23], an evolutionary conservation analysis was performed aligning its sequence with the mouse-predicted *CENP-F* gene. No significant similarities were found indicating that in rodents a putative *CENP-F* antisense regulatory role, if any, would be associated to a different class of noncoding elements. Despite its high sequence similarity with other human *Alus, 21A* lacks the *Alu-*specific intragenic consensus elements needed to promote its pol III transcription such as the blocks A and B [24]. This observation further pointed to a *21A* transcription driven by an extragenic type 3 pol III promoter.

**Figure 2:**
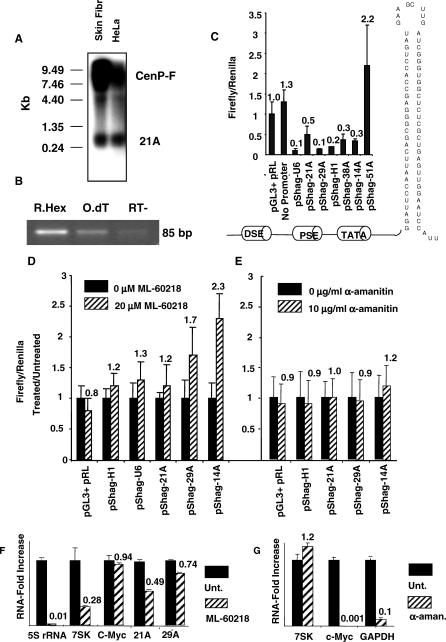
Pol III-Dependent Synthesis of Novel Transcription Units (A) Northern blot analysis of human skin fibroblasts and HeLa cells. Results show two bands: the first (detected at about 300 nucleotides) being the *21A* endogenous product and the second (of a very high molecular mass) representing *CENP-F* mRNA. (B) *21A*-specific RT-PCR amplification. As expected for nonpolyadenylated transcripts, an efficient amplification product was obtained only in the random hexamers-primed reactions. (C) Promoter activity transfection assay. A specific luciferase-silencing hairpin is transcribed by six PSE/DSE-dependent promoter elements *(11A* [*H1* RNA], *14A, 21A, 29A, 38A, 51A).* A view of the silencing constructs including the hairpin nucleotide sequence is enclosed. The promoter region encompasses the putative pol III type 3 regulatory regions (TATA box, PSE, and DSE). pGL3 + pRL, negative control; pSHAG-*U6,* canonical pol III promoter; No promoter, hairpin without PSE/DSE-dependent promoter thus resulting transcriptionally inactive. (D and E) Promoter-activity transfection assay in presence/absence of 20 μM ML-60218 cell-permeable pol III inhibitor or 10 μg/ml α-amanitin pol II–specific inhibitor. Results are reported as luciferase emission of treated versus untreated samples. (F) Real-time RT-PCR analysis of transcript levels for *21A* and *29A* transcription units, two known pol III–transcribed genes *(7SK* and *5S rRNA),* and one pol II–transcribed gene *(c-Myc)* in ML-60218 treated/untreated cells, as resulting after normalization of each treated sample with its untreated counterpart. All the measures were referred to a ML-60218 unaffected pol II housekeeping gene *(GAPDH).* (G) Real-time RT-PCR analysis of the RNA level of two known pol II–transcribed *(c-Myc* and *GAPDH)* and one pol III–transcribed *(7SK)* genes in α-amanitin treated/untreated cells as resulting after normalization of each treated sample with its untreated counterpart. All the measures were referred to an α-amanitin unaffected pol III gene *(5S rRNA).*

To check for *21A* expression in cultured cells, we performed Northern blot analysis on total HeLa cell RNA and skin fibroblast RNA using a *21A* dsDNA probe. Two positive bands were detected: one corresponding in size to the expected *21A* transcript (∼300 nucleotides), and the other one corresponding to a high molecular mass transcript (as expected for *CENP-F* mRNA) (Figure 2A). However, considering that the *21A* double-strand cDNA probe would detect transcription of *21A-*similar *Alus* from multiple loci, we also amplified a *21A-*specific cDNA from total RNA samples, extracted from skin fibroblasts and four tumor cell lines (293T, LAN5, HCT, and HeLa), by random hexamer-based RT-PCR in order to better identify a *21A-*specific transcription product (Figure 2B). The DNA band obtained was then purified and sequenced, evidencing that the amplification product was the expected *21A*. In addition, to better assess *21A* transcription, we fused its promoter region to a luciferase silencer hairpin and cotransfected this construct with a plasmid-expressing luciferase. Results showed a halved luciferase activity 48 h after transfection, thus demonstrating an efficient transcription directed by *21A* promoter. In the same experiment, a set of five novel promoters from our collection was tested, demonstrating an active transcription of the hairpin promoted by four of them (Figure 2C). These data support the conclusion that the majority of the novel putative transcription units is under the control of active extragenic PSE/TATA-containing promoters.

### Pol III–Dependence of the Novel Transcription Units

The same experiment as above was repeated after 24 h of cell treatment with ML-60218, a cell-permeable indazolo-sulfonamide compound that displays broad-spectrum inhibitory activity against pol III [25]. Results showed an efficient luciferase-silencing activity in the absence of the pol III inhibitor (as evidenced by a decreased luciferase emission), while after treatment with ML-60218 the luciferase signal was increased (Figure 2D). As a control, a similar experiment was performed by treating the cells for 12 h with a pol II inhibitor (α-amanitin). In this case, no major transcription variations were observed either for the novel transcript units or for the well-known pol III-dependent *H1* gene (Figure 2E).

To better assess pol III dependence of *21A* transcription, we directly measured the endogenous *21A* (and *29A*) RNA amount in ML-60218 treated cells versus untreated control samples. Results evidenced a significant transcription downregulation of the two novel pol III units (50% and 30% inhibition in the case of *21A* and *29A,* respectively), thus keeping in line with the occurrence of a specific effect of ML-60218 on their promoters.

To further assess the specificity of action of the two inhibitors, we analyzed by real-time RT-PCR the transcription activity of two pol III *(5S rRNA* and *7SK)* and two pol II-dependent genes *(c-Myc* and *glyceraldehyde 3 phosphate dehydrogenase [GAPDH])* both in ML-60218 and in α-amanitin-treated cells. Results showed a significant inhibition of the pol III–dependent genes after ML-60218 treatment and a stable transcription level of the pol II–dependent genes in the same samples (Figure 2F). On the contrary, pol III transcription activity was stable in α-amanitin–treated cells, while in the same samples the pol II–transcribed genes were downregulated, thus demonstrating the specificity of action of the two inhibitors in these experimental conditions (Figure 2G). Altogether, these results provide evidence that the novel PSE/TATA-containing transcription units are transcribed by pol III.

### *21A* Acts as *CENP-F* Regulatory Cogene Modulating Its Expression at Posttranscriptional Level

**Figure 3:**
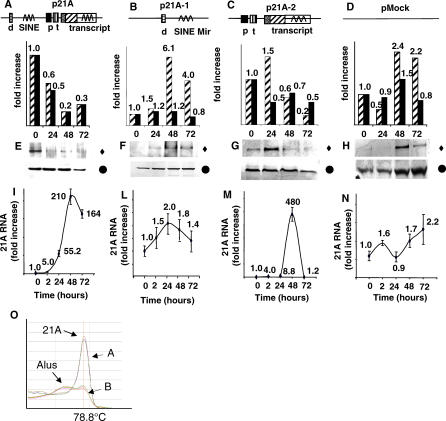
*21A-*Driven *CENP-F* Expression Regulation (A–D) *21A* constructs. d, DSE Element; p, PSE Element; p*21A,* whole transcription unit; p*21A-1,* upstream, DSE-containing region; p*21A-2,* transcription region; pMock, empty vector; t, TATA box. (E–H) CENP-F protein expression level after 0, 24, 48, and 72 h of constructs transfection. Full triangle, anti-CENP-F antibody; Full circle, anti-tubulin antibody (for protein loading normalization); Striped columns, quantitative determination of CENP-F expression modulation as determined by Western blot analysis; Full columns, quantitative determination of *CENP-F* mRNA expression modulation as determined real-time RT-PCR analysis. (I–N) *21A* RNA level in transfected samples indicating that the exogenous *21A* expression inversely correlates with CENP-F protein expression. (O) Dissociation curve of *21A* amplification products. A, *21A*-transfected HeLa cells; B, untransfected HeLa cells showing the very low basal *21A* transcription level.

To test whether the *21A* transcript acts as an antisense inhibitor of *CENP-F* expression, we measured by Western analysis *CENP-F* protein level in HeLa cells transiently transfected with four different *21A* constructs carrying: (i) the whole *21A* region containing both DSE and PSE elements *(p21A);* (ii) its upstream moiety, which contains the DSE and a MIR element, but not the *CENP-F* homology region *(p21A-1);* (iii) the novel pol III type 3–transcription region (which includes an *Alu Jb* module) *(p21A-2);* and (iv) an empty vector as mock control (pMock). As shown in Figure 3, starting at 24 h from transfection of the whole *21A* region, inhibition of *CENP-F* accumulation (followed by a rapid degradation) was observed. Such inhibition was specifically associated to constructs expressing the *21A* RNA *(p21A, p21A-2),* while the MIR element in the upstream moiety of the fragment (p*21A-1* construct) was ineffective (Figure 3A–3D).

In this context, it has to be noted that a slight delay occurred in *21A-2* inhibitory action (and in the expression of *21A*-specific RNA), compared to what was observed with the complete *21A* construct (more rapid increase in *21A* RNA expression and decrease of CENP-F protein levels), suggesting a positive transcriptional role of the DSE element. The actual occurrence of *21A* transcription in transfected cells was analyzed by real-time quantitative RT-PCR. As expected, a very high amount of *21A* transcript was detected in p*21A* and p*21A-2*–transfected cells (210- and 480-fold, respectively, at 48 h from transfection), while the *21A* RNA content of samples transfected with pMock control plasmid and/or with a construct containing the promoter lacking the transcribed region (p*21A-1* construct) were essentially stable, showing a very low basal level of *21A* expression in untransfected HeLa cells (Figure 3I–3N). All the PCR products were analyzed in their dissociation curve, showing a single characteristic pick (at 78–79 °C) in p*21A*/p*21A2*-transfected samples significantly reduced in pMOCK/p*21A-1.* On the contrary, the cells transfected with the two control plasmids (pMock/p*21A-1*) showed a dissociation pattern characteristic of a heterogeneous population of molecules (Figure 3O). Again these results confirmed an active synthesis of the exogenous *21A* ncRNA transcript in p*21A*/p*21A-2*–transfected samples that was strongly reduced at a very low endogenous–basal level in the samples lacking the transcript region (pMOCK/p*21A-1*). As a consequence of *21A* very active transcription, the level of *CENP-F* mRNA (as determined by real-time RT-PCR) was significantly decreased in p*21A*/p*21A-2*–transfected cells, while no major *CENP-F* mRNA variations were observed in pMOCK/p*21A-1*–transfected cells (Figure 3E–3H). Altogether these results demonstrate an inverse correlation between *21A* transcription and *CENP-F* expression. Therefore, considering the high sequence homology between *21A* transcript and three *CENP-F* hnRNA intronic portions, we suggest a mechanism of antisense inhibition of *CENP-F* mRNA maturation by the *21A* transcript.

### *21A* Overexpression Specifically Inhibits Cell Proliferation in Humans

**Figure 4:**
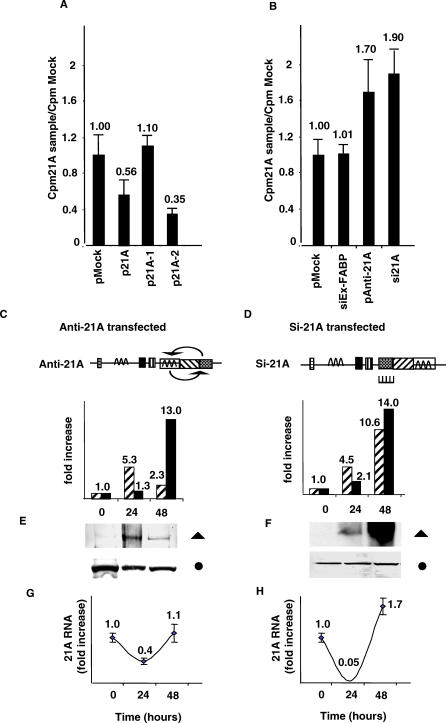
Modification of Cell Proliferation Rate in *21A-*Overexpressing HeLa Cells (A) Proliferation inhibition of HeLa cells after 48 h of *21A* constructs transfection. Results emphasize the specificity of the *Alu Jb*-containing region as a source of proliferation inhibitory transcripts. (B) Proliferation increase of HeLa cells after 48 h of pAnti-*21A* and si*21A* transfection. siEx-FABP, unrelated chicken-specific siRNA (negative control). (C and D) Constructs structures. Anti-*21A,* the transcript region is inverted and the construct maintains *21A* promoter as well as its termination site. *si21A,* siRNA *21A*-specific. (E and F) CENP-F protein expression level after 0, 24, and 48 h of constructs transfection. Full triangle, anti-CENP-F antibody; full circle, anti-tubulin antibody; striped columns, quantitative determination of CENP-F expression modulation as determined by Western blot analysis; full columns, quantitative determination of *CENP-F* mRNA expression modulation as determined real-time RT-PCR analysis. (G and H) *21A* RNA level in transfected samples indicating that the endogenous *21A* expression is inhibited after 24 h of anti-*21A*/si*21A* transfection.

Given the central role of *CENP-F* in mitosis, we tested the effect of ectopic *21A* expression on cell proliferation. By measuring [^3^H]-thymidine incorporation, we observed a dramatic arrest of cell proliferation after 48 h in *21A*-transfected cells. Again, the effect was specifically associated to the downstream *21A* transcribed region (p*21A*/p*21A-2* constructs), while transfection of the MIR-containing upstream moiety (p*21A-1* construct) did not alter cell proliferation (Figure 4A). Although at the present state we cannot exclude a contribution to this effect by *Alus* from other loci, this experiment demonstrates an inverse correlation of *21A* transcription and cell proliferation that is in accord with the inhibition of *CENP-F* synthesis demonstrated above.

To further support the antisense role of *21A,* we transfected HeLa cells with a construct expressing the transcript in antisense configuration (here referred to as pAnti-*21A*), thus quenching the activity of the endogenous *21A* molecules. Results showed an increased cell proliferation 24–48 h after transfection. Similar results were obtained when a *21A*-specific small interfering RNA (siRNA)–expressing construct was transfected in HeLa cells, while the negative control sample (cells transfected with an unrelated chicken-specific siRNA) maintained a cell-proliferation rate similar to that of pMock-transfected cells (Figure 4B). In both of the experiments an increased *CENP-F* expression was detected both at the protein and mRNA levels (Figure 4E and 4F). As evidenced by real-time RT-PCR in the same experiment, a concomitant *21A*-RNA decrease was observed after 24 h of transfection in anti/si*21A* treated cells, although a complete recovery of *21A* RNA synthesis occurred after 48 h of transfection (Figure 4G–4H). As shown in these experiments *CENP-F* modulation and *21A* RNA decrease were analyzed only at 0, 24, and 48 h after transfection, rather than at 0, 24, 48, and 72 h as in the previous experiments. In fact, at 72 h after transfection the *CENP-F* synthesis determinations would be strongly biased by an early cell culture overconfluence caused by the proliferation increase that follows *21A* downregulation.

These data suggest that the decreased amount of *21A* transcript consequent to its siRNA-mediated silencing, as well as its suppression by antisense technology, specifically increases *CENP-F* synthesis, thus keeping in line with the proposed role of *21A* as *CENP-F* regulatory cogene. In addition, it has to be considered that the increased proliferation rate observed here supports the idea of a widespread regulatory action of *21A* that may control at the posttranscriptional level the expression of several target genes similarly to what has been proposed for miRNAs [26].

### The *21A* Regulatory Effect Is Human Specific

**Figure 5:**
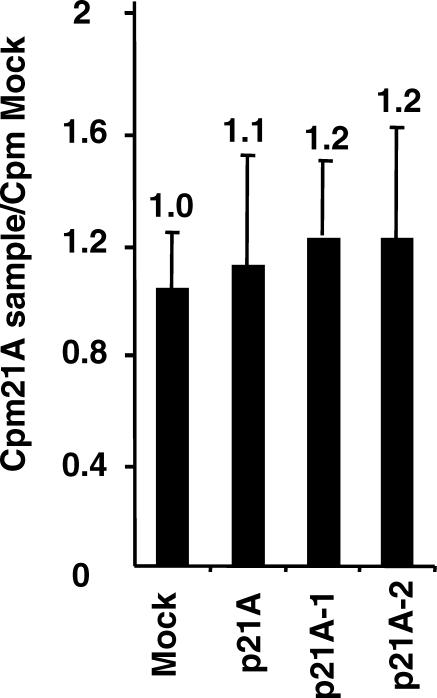
Mouse NIH-3T3 Cell Proliferation Rate after Transfection of *21A* Constructs No proliferation decrease was observed.

Considering that a *21A*-driven cell-proliferation inhibition is expected to be primate specific (*Alu* sequences were not found in other mammalian orders), we tested for its possible occurrence in mouse. We found that, after transfection of p*21A,* p*21A-1,* p*21A-2,* and pMock, the murine fibroblast NIH 3T3 cells did not show any proliferation decrease as assessed by [^3^H]-thymidine incorporation (Figure 5). The species-specificity of *21A* action, together with its inability to cause a nonspecific cell reaction that leads to a proliferative blockade in mice, further strengthens a *21A*-specific (perhaps multilocus) regulatory role.

In fact, considering these data we rule out a nonspecific effect of *21A* on cell proliferation, perhaps due to the activation of a more general biological process, such as the interferon response (an antiviral cell reaction shared by all mammals), rather than a specific multilocus *21A* regulatory action.

### *21A* Endogenous ncRNA Is Downregulated in Tumor Cell Lines

**Figure 6:**
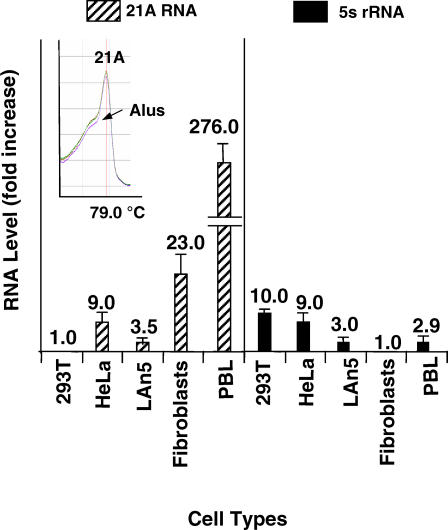
Real-Time RT-PCR Analysis of *21A* Endogenous RNA in Different Cell Types with Respect to the Less Abundant Samples (293T for *21A* RNA and Fibroblasts for *5S* rRNA) Striped columns, *21A* RNA; full columns, *5S* rRNA. The dissociation curve of *21A* amplification product in PBL is reported in the inset.

As demonstrated by transfection experiments, *21A* overexpression is inversely correlated to cell proliferation. In accordance with this finding, its basal expression level is very low in fully proliferating HeLa cells. To better investigate the inverse correlation between the endogenous *21A* expression and cell proliferation, we analyzed by quantitative real-time RT-PCR the *21A* expression levels in cell types characterized by different proliferation potentials. Results showed that in three immortalized, fully proliferating cell lines analyzed here (HeLa as cervical adenocarcinoma; 293T as renal epithelial adenovirus transformed cells; LAN5 as neuroblastoma), the level of *21A* transcription was very low if compared to the unproliferating/resting PBL (peripheral blood lymphocyte) cells, in which a 276-fold-increased–*21A* transcription was evidenced. In the same experiment, according to an inverse correlation between endogenous *21A* transcription and the cell proliferation rate, the *21A* RNA level in primary skin fibroblasts (of which the proliferation rate is significantly lower than that of the tumor cell lines analyzed here) showed a 23-fold increase compared to 393 T cells, and a very low expression level if compared to the resting/unproliferating PBL (Figure 6). Again the dissociation curve analysis of *21A* amplification product showed in PBL a peak at 78–79 °C, characteristic of a single specific molecular species that resembled the one obtained in *21A*/*21A-2* transfected cells (where the amount of *21A* transcripts was strongly increased), although a slight shoulder, most likely due to a cross-amplification of other very similar transcripts, revealed a detectable endogenous *Alu* transcription background (Figure 6). Altogether these results evidence a very active *21A* transcription in PBL/resting cells that further strengthens the idea of *21A* as a novel key factor of cell-proliferation control.

In order to check if the endogenous *21A* overexpression in unproliferating cells was related to a widespread increased RNA polymerase III activity rather than a *21A*-specific activation, we measured by real-time RT-PCR the *5S* rRNA expression level in the same samples. The results showed no direct correlation between *5S* rRNA expression and the cell-proliferation rate variations, evidencing that the *21A* overexpression in resting cells was the consequence of a *21A*-specific transcription activation rather than a wider, nonspecific increase of pol III activity (Figure 6). These data thus suggest the existence of an unexpectedly specific expression regulation of *21A* promoter (related to the cell proliferation state) that needs to be investigated in detail.

### Conclusions

We propose that the noncoding fraction of the human genome includes a larger than expected number of ncRNA genes controlled by DSE and PSE promoter elements. Due to their promoter structure, a number of these genes is likely to be transcribed by pol III. We refer to them as cogenes since they could specifically coact with a protein-coding pol II gene. Given the very high sequence homology between pol III and pol II transcript pairs, and in light of the results we have obtained investigating the regulatory activity of the *21A* transcription unit, we propose that a large part of these novel elements may act as antisense inhibitors of protein translation and/or mRNA maturation, although some of them (those whose homology with the pol II target gene is in sense configuration) could play a role in gene-expression regulation with different mechanisms. Altogether these findings provide evidence for the existence of an ncRNA gene set associated to PSE/DSE-containing promoters, whose products coact with a corresponding set of protein-coding targets.

In conclusion, this study provides: (i) a collection of novel noncoding transcripts to be investigated for their potential regulatory action with respect to pol II target genes; (ii) a novel source of PSE-dependent promoters useful for the identification of common regulatory regions specific for this type of promoters; (iii) a novel class of molecules involved in the RNA-dependent gene expression regulation; and (iv) a novel transcript (*21A*), whose role in tumor cell proliferation deserves further investigation in the context of cancer studies.

## Materials and Methods

### Databases and searches.

All of the sequence searches and alignments were carried out by means of BLAST at the National Center for Biotechnology Information (http://www.ncbi.nlm.nih.gov/BLAST). The sequences used to query were the following: *H1* PSE, nCACCATAAAnGTGAAAn or nTTTCACnTTTATGGTGn; *U6* PSE, CTTACCGTAACTTGAAAGT; *7SK* PSE (as reported in PMID: 2011518), TTGACC-TAAGTG; DSE (*Octamer-Binding Transcription Factor 1 [Oct1]* consensus sequence), ATTTGCAT or ATGCAAAT with or without a single base of mismatch.

### Cell culture, transfection, and luciferase assay.

For transient transfections, HeLa cells (grown in DMEM supplemented with 10% FCS), were grown in multiwell petri dishes 16 h before transfection. The expression *(21A, 21A[1], 21A[2], 21A[3])* constructs containing the regions of interest cloned in the pTopo vectors (Invitrogen, http://www.invitrogen.com) were introduced into the cells using the Fugene 6 transfection reagent (Roche, http://www.roche.com/home.html) according to the manufacturer's instructions. A plasmid expressing luciferase was used as a control of transfection efficiency (to which all the results were normalized). Cells were harvested 24, 48, and 72 h after transfection, and firefly luciferase activity was measured by dual-luciferase reporter–assay system (Promega, http://www.promega.com) according to the manufacturer's protocol. To specifically inhibit RNA polymerase III and/or RNA polymerase II, a cell-permeable chlorobenzenesulfonamide (ML-60218) (Calbiochem, http://splash.emdbiosciences.com) and/or α-amanitin (Roche, http://www.roche.com) were used at the concentration of 20 μM and 10 μg/ml, respectively, in the medium for 25 h (ML-60218) and 12 h (α-amanitin) before the luciferase activity detection.

### RNA interference–silencing assay.

To test the promoter activity of the novel transcription units, we prepared six plasmid constructs expressing a firefly luciferase-silencing hairpin (Gregory Hannon, Cold Spring Harbor Laboratory, Cold Spring Harbor, New York, United States); transcription was driven by the *11A, 14A, 21A, 29A, 38A, 51A* promoters, respectively. The hairpin sequence (targeting a firefly luciferase mRNA from a cotransfected expression plasmid [Promega]) is: 5′-GGAUUCCAUUCAGCGGAGCCACCUGAUGAAGCUUGAUCGGGUCUCGCUGAGUUGGAAUCCAUU-3′. Oligos used to subclone the novel pol III type III promoters within Not I/HinD III restriction sites (uppercase letters) were the following:

11AFprom Not I: 5′-atgcGCGGCCGCatttgcatgtcgctatgtg-3′

11ARprom HinD III: 5′-gatcAAGCTTcatcaggtggctcccgctgaattggaatccacgcactcagctcgtg-3′

14AFprom Not I: 5′-atgcGCGGCCGCaactgatgtatgattatatctt-3′

14ARprom HinD III: 5′-gatcAAGCTTcatcaggtggctcccgctgaattggaatccattattatctcctttgttctgt-3′

21AFprom Not I: 5′-atgcGCGGCCGCacagctgtagcagatgct-3′

21ARprom HinD III: 5′-gatcAAGCTTcatcaggtggctcccgctgaattggaatccaccacacttggtcaactat-3′

29AFprom Not I: 5′-atgcGCGGCCGCttctcacctaaaggagtc-3′

29ARprom HinD III: 5′-gatcAAGCTTcatcaggtggctcccgctgaattggaatccttctaatcctcctaagatca-3′

38AFprom Not I: 5′-atgcGCGGCCGCttcactaagatccagtgc-3′

38Arprom HinD III: 5′-gatcAAGCTTcatcaggtggctcccgctgaattggaatccgattcatgaacacagaatatt3′

51AFprom Not I: 5′-atgcGCGGCCGCgttgaacatttaactctgtat-3′

51Arprom HinD III: 5′-gatcAAGCTTcatcaggtggctcccgctgaattggaatccctcatggcacttggagat-3′

In this analysis, the above constructs were cotransfected with a pGL3 plasmid-expressing (Promega) firefly luciferase as a target to be silenced and with a pRL plasmid expressing (Promega) renilla luciferase to which all the determinations were normalized. Cells were harvested 24, 48, and 72 h after transfection, and firefly/renilla luciferase activities were measured by dual-luciferase reporter–assay system (Promega) according to the manufacturer's protocol.

### Plasmid constructs generation and sequencing.

The plasmid constructs *p21A, p21A(1),* and *p21A(2)* were generated amplifying from a genomic DNA preparation of the regions of interest; the PCR products were then subcloned into the pNEB193 vector. The oligos used to generate *p21A* PCR fragments were the following:

*21A* forward: 5′-GGAAATCTTACCTTCCTGCC-3′

*21A* reverse: 5′-TGGCTAGGTCATGTGACCAT-3′

*21A(1)* forward: 5′-GGAAATCTTACCTTCCTGCC-3′

*21A(1)* reverse: 5′-TTCATTCATTCATTCATTGATTCAC-3′

*21A(2)* forward: 5′–CAGCTGCAGCAGATGCTAGCAGGGC-3′

*21A(2)* reverse: 5′–TGGCTAGGTCATGTGACCATTC-3′

The plasmid construct pAnti-*21A* was generated amplifying the transcribed region from *p21A* plasmid using the following oligos:

Anti-*21A* Terminator-containing forward: 5′-CTGAAAAAGTAGTCCCAGCACTTTG-3′

Anti-*21A* Bam HI-containing reverse: 5′-ATGCGGATCCGAGACAGGGTCTTGCTC-3′

Thus the transcribed region was generated in antisense configuration. The p*Anti-21A* promoter was obtained by amplifying p*21A* promoter with the following oligos:

*21A* Forward: 5′-GGAAATCTTACCTTCCTGCC-3′ p21A Bam HI-containing reverse: 5′-ATGCGGATCCGAGCCACCACACTTGGTC-3′. The PCR products were digested with the restriction enzyme Bam HI, purified by gel electrophoresis, and ligated by T4 ligase (Invitrogen). The insert obtained was then subcloned in pTOPO vector (Invitrogen) following the manufacturer's instructions. Prior to transfection all of the plasmids were sequenced by DNA sequencing kit (Applied Biosystems, www.appliedbiosystems.com) following the manufacturer's instructions.

### RT-PCR reactions.

To isolate and sequence a partial *21A* cDNA, we performed different RT-PCR reactions. Starting from about 5 μg of total RNA, cDNA was synthesized by using an oligo (dT)_12–18_ primer or a random hexamers mix and a superscript first-strand synthesis system for RT-PCR (Invitrogen). cDNAs were diluted 10–50 times, then subjected to PCR reactions. The oligos used to isolate *21A* RT-PCR product were: oligo forward 21AF 5′-gctcacgtagtcccagcacttt-3′ and oligo reverse 21AR 5′-actatgttgcccaagctggtct-3′. PCR products were separated on 1.5%–2% agarose gel. The DNA bands were cut, purified by the Millipore DNA gel extraction kit (http://www.millipore.com), and sequenced.

### Real-time quantitative RT-PCR.

The RNA for *21A* was measured by real-time quantitative RT-PCR using the PE ABI PRISM@ 7700 sequence detection system (PerkinElmer, http://www.perkinelmer.com) and Sybr Green (Applied Biosytems). The sequences of *21A* forward and reverse primers as designed by the Primer Express 1.5 software (Applied Biosystems) were 5′-GCTGAGGCAGGAGGATCACT-3′ and 5′-GCACTACCACACCCAGCTAATTTT-3′. The sequences of *CENP-F* forward and reverse primers were 5′-CTGCAGAAAGAACTCTCTCAACTTC-3′ and 5′-TCAACAATTAAGTAGCTGGAACCA-3′. For endogenous control, the expression of *GAPDH* gene was examined. The sequences for human *GAPDH* primers were 5′-GAAGGTGAAGGTCGGAGTC-3′ and 5′- GAAGATGGTGATGGGATTTC-3′. The sequences for human *5S* rRNA primers were 5′-TACGGCCATACCACCCTGAA-3′ and 5′-GCGGTCTCCCATCCAAGTAC-3′. The sequences for human *7SK* RNA primers were 5′-AGGACCGGTCTTCGGTCAA-3′ and 5′-TCATTTGGATGTGTCTGCAGTCT-3′. The sequences for human *c-Myc* primers were 5′-CGTCTCCACACATCAGCATAA-3′ and 5′-GACACTGTCCAACTTGACCCTCTT-3′. Relative transcript levels were determined from the relative standard curve constructed from stock cDNA dilutions and divided by the target quantity of the calibrator following manufacturer's instructions.

### Anti-*21A* siRNA synthesis.

The Anti-*21*A siRNA was synthesized against a region of the *21A* transcript of no homology with *CENP-F* so that the silencing effect was specific for the pol III regulatory RNA and did not interfere with *CENP-F* RNA stability. The siRNA synthesis was carried out taking advantage of the siRNA construction kit (Ambion, http://www.ambion.com) according to the manufacturer's protocol. The sense/antisense oligos used were: 5′-aaGTGTGGTGGCTCACcctgtctc-3′ and 5′-aaGTGAGCCACCACACcctgtctc-3′.

### Proliferation assay.

We tested proliferation of HeLa cells transfected with *21A, 21A-1, 21A-2, 21A-3,* and Anti-*21A* constructs plating 5 × 10^5^ cells per well in round-bottomed 96-well plates, incubated for 24, 48, and 72 h after transfection, and pulsed with [^3^H]-thymidine (1.0 μCi/10 μl/well) (Amersham Biosciences, http://www5.amershambiosciences.com) for the last 18 h. We harvested the cells and evaluated cell proliferation by counting the thymidine uptake. We calculated the averaged proliferation rate, measured as counts per minute, and standard deviation for the triplicate wells of each sample.

### RNA isolation and Northern blot analysis.

Based on a single-step acid-phenol–guanidium method, total RNA was extracted using TRIzol reagent (Invitrogen) according to the manufacturer's protocol. Total RNAs, from HeLa cells, were electrophoresed through 1.5% agarose gels in the presence of formaldehyde and blotted onto Hybond N membranes (Amersham). The blot was hybridized with an 85-bp–long probe contained in the region from nucleotide 1,194 to nucleotide 1,278 of the *21A* reported sequence (Table S2), spanning a region internal to the transcript. The probe was obtained by PCR (using the *21A* plasmid construct as template) using the following oligos: 21AF 5′- GCTCACGTAGTCCCAGCACTTT-3′ and 21AR 5′-AGACCAGCTTGGGCAACATAGT-3′. Blot prehybridization was performed at 65 °C for 2 h in 333 mM NaH2PO4 (pH 7.2), 6.66% sodium dodecyl sulphate, and 250 mg/ml denatured salmon sperm DNA. Blot hybridization was performed at 65 °C for 18 h in the same solution containing 10^6^ counts per minute/ml of denatured and labeled probes. After hybridization the blots were washed twice at 65 °C for 30 min in 0.2% sodium dodecyl sulphate, 2× SSPE and once at 65 °C for 30 min in 0.2% sodium dodecyl sulphate, and 0.2× SSPE. Membranes were exposed to autoradiographic films for 24–48 h and then developed.

### Western blot analysis.

Equal amounts of proteins (10 μg/sample) from each sample were loaded on standard 4%–12% NU-PAGE gradient gels (Invitrogen). Blotting onto Protran nitrocellulose membranes (Schleicher & Schuell, www.schleicher-schuell.com) was performed in the X-Cell Sure Lock Electrophoresis Cell (Invitrogen), according to the manufacturer's instructions. The membranes were saturated overnight in 3% nonfat milk in TTBS buffer (500 nM NaCl; 20 mM Tris/Cl [pH 7.5]; 0.05% Tween-20) and incubated for 4 h at room temperature with the human anti-mitosin/CENP-F ab90 (ABCAM, http://abcam.com) and/or anti-alpha tubulin (OMIM 191110) (Sigma-Aldrich, www.sigmaaldrich.com) mouse monoclonal antibodies. The anti-mitosin antibody recognized a weak signal at a very high apparent molecular mass (350–400 kDa), while the anti-alpha tubulin showed a clear signal at 45 kDa. The immunoreactive band was revealed by an alkaline phosphate-conjugated affinity-purified monoclonal anti-rabbit–mouse IgG (Sigma-Aldrich), and (in the experiment indicated in Figure 1C) the enzymatic chemiluminescence (ECL) detection system (Amersham), or (in the experiment indicated in Figure 1E) the alkaline phosphatase substrate BCIP/NBT (ICN Biomedicals, http://www.mpbio.com).

## Supporting Information

Table S1 Schematic Representation of PSE-TATA Box Distances (52 KB PPT)

Table S2 Table of Sequences (104 KB DOC)

Table S3 Putative Secondary Structures (429 KB PPT)

Table S4 Sequence Analysis by Repeat Masker (45 KB RTF)

Table S5 Transcription Unit Features (74 KB DOC)

### Accession Numbers

The National Center for Biotechnology Information (http://www.ncbi.nlm.nih.gov) accession numbers for the genes and gene products discussed in this paper are: *U6* (M14486), *7SK* (001445), *H1* (002312), *U2* (002716), *CENP-F* (016343), *5S rRNA* (V00589), *c-Myc* (002467), and *GAPDH* (002046).

## Abbreviations

CENP-F: centromeric protein F
DSE: distal sequence element
GAPDH: glyceraldehyde 3 phosphate dehydrogenase
MIR: mammalian interspersed repeat
miRNAs: microRNAs
nc: noncoding
pol: polymerase
PSE: proximal sequence element
siRNA: small interfering RNA
snRNA: small nuclear RNA
RT-PCR: reverse transcriptase PCR
