# Supplementary material for: New Small Nuclear RNA Gene-Like Transcriptional Units as Sources of Regulatory Transcripts
Source: PLoS Genet. 2007 Feb 2;3(2):e1. doi: 10.1371/journal.pgen.0030001 (PMC1790723; doi:10.1371/journal.pgen.0030001)
Supplement: Supplementary file 1 [file pgen.0030001.st004.rtf]

Table S4: Sequence analysis by RepeatMasker Web Server (available at : http://www.repeatmasker.org/cgi-bin/WEBRepeatMasker)


Seq Name	Seq. Length	Rep. Length	Position^a^	Type	
					
11A	-	-	-	-	
12A	-	-	-	-	
14A	-	-	-	-	
17A	156	115	42-156	Mir (MIR3)	
19A	-	-	-	-	
20A	-	-	-	-	
21A	324	307	9-315	SINEs (AluJB)	
22A	-	-	-	-	
23A	-	-	-	-	
24A	304	302	1-302	Line L1 (L1MC)	
27A	-	-	-	-	
29A	346	287	58-344	SINEs (AluJB)	
30A	142	135	8-142	LTR/MALR (MLT1G3)	
31A	-	-	-	-	
32A	128	122	7-128	LTR (LTR7)	
33A	-	-	-	-	
34A	-	-	-	-	
35A	-	-	--	-	
36A	-	-	-	-	
37A	50	49	2-50	LINE (L1M4)	
38A	350	341	1-341	LINE (L1M3)	
39A	-	-	-	-	
40A	485	220	254-473	MIR (MIR b)	
41A	-	-	-	-	
42A	-	-	-	-	
43A	-	-	-	-	
44A	223	104	107-210	LTR/MALR (MLT1M2)	
45A	-	-	-	-	
47A	-	-	-	-	
48A	-	-	-	-	
49A	-	-	-	-	
50A	-	-	-	-	
51A	-	-	-	-	
52A	-	-	-	-	

^a^ Positions are given with respect to the putative transcription start site
