# Supplementary material for: New Small Nuclear RNA Gene-Like Transcriptional Units as Sources of Regulatory Transcripts
Source: PLoS Genet. 2007 Feb 2;3(2):e1. doi: 10.1371/journal.pgen.0030001 (PMC1790723; doi:10.1371/journal.pgen.0030001)
Supplement: Supplementary file 2 [file pgen.0030001.st002.doc]

TABLE S2


Table of Sequences

.	The predicted TATA box, PSE and DSE consensus sequences (in sense as well as in antisense configurations) are indicated in red.
.	The putative transcribed regions are underlined, with the transcription start site (TSS) indicated in blue. Except when otherwise indicated, the TSS was predicted by moving 30 bp downstream of the TATA or AT-rich element and selecting the closest pyrimidine-purine sequence motif. The purine of this motif was assumed to be the TSS. A run of at least 4 consecutive T residues (T_4_) was generally considered as a transcription termination signal. In some cases, a T_4_ element was found very close to the TSS; in these cases, it was assumed that Pol III could Òread-throughÓ the T_4_ element (as documented in the literature) and terminate to the subsequent termination signal. The predicted termination signals are in red.
０.	The 21A region in antisense configuration with respect to CenPF mRNA is indicated in green.


Human Genome Map 14q11.2 (784bp sequence)
11A
 
ATTTGCATGTCGCTATGTGTTCTGGGAAATCACCATAAACGTGAAATGTCTTTGGATTTGGGAATCTTATAAGTTCTGTATGAGACCACTCTTTCCCATAGGGCGGAGGGAAGCTCATCAGTGGGGCCACGAGCTGAGTGCGTCCTGTCACTCCACTCCCATGTCCCTTGGGAAGGTCTGAGACTAGGGCCAGAGGCGGCCCTAACAGGGCTCTCCCTGAGCTTCGGGGAGGTGAGTTCCCAGAGAACGGGGCTCCGCGCGAGGTCAGACTGGGCAGGAGATGCCGTGGACCCCGCCCTTCGGGGAGGGGCCCGGCGGATGCCTCCTTTGCCGGAGCTTGGAACAGACTCACGGCCAGCGAAGTGAGTTCAATGGCTGAGGTGAGGTACCCCGCAGGGGACCTCATAACCCAATTCAGACTACTCTCCTCCGCCCATTTTTGGAAAAAAAAAAAAAAAAAAAAAACAAAACGAAACCGGGCCGGGCGCGGTGGTTCACGCCTATAATCCCAGCACTTTGGGAGGCCGAGGCGGGCGGATCACAAGGTCAGGAGGTCGAGACCATCCAGGCTAACACGGTGAAACCCCCCCCCATCTCTACTAAAAAAAAAAAATACAAAAAATTAGCCATTAGCCGGGCGTGGTGGCGGGCGCCTATAATCCCAGCTACTTGGGAGGCTGAAGCAGAATGGCGTGAACCCGGGAGGCGGAGCTTGCAGTGAGCCGAGATCGCGCCACTGCATTCCAGCCTGGGCGACAGAGCGAGTCTCAAAAAAAAAAAAACC


Human Genome Map 2p24.3  (3000 bp sequence)
12A 

TGTATTTTAAATTATGATACATAATGACTATTTAACTTCCAAACAGAATTCACTCATTTACATTTATGAACATTCTGGGTATAATATCCAGAGGGAATTAAACCACTATCTCAGAGAGATATCTGCATTCTGATGTTCACTGAAACATTATTCACAGTAGCCAAAATACAGAAACAACCTGTCTGTCAACGAATTAATGGATAAATAAAAGAGATAAGGAATATATATATACACACACATACACACACAAGCACACACACACACATACAATGGAAAATTATTCATCCTAAACGGAAATAAAATTCTGCTATTTACAAGAAGAAGAATGAAACTGGAGGACCTTCTGCTTAGAGAAATAAGTCAGACATAGAAAGACATATACTGCATGATCTGACTTGTATGTGGAATATAAAAAAGTAGAACTCATGAAAATAGAGTAGAAGGGTGGTTACCAGAAGTTATGGGGTGGGAGAAATGGAGAGCTATTGGTCCAAGGATGCACACTTTGAATCATAAGGAATAAGTTCTGGAGACCTGATGTGCAGTAGGATGACTATAGTTAATAATCATGTATTATATGCTTGAAATTTGCTAAGAGAATAGATATTCAGTATTCTTACAACACACACAGACACACACACACACAGGTATGTCAGGTGATGGATATGTCAATTAGCTTGATTGTGGTGATCATTTTTATAATATATACATATATCAAAATAGTATATTTCCAGTTTTTCACTTTTCTTTTAATTTTTATTATCATATATTTTACTATATAAAATATTTTTAACTAACATGATGTCAGTCCAGCCTGACCAACATGGAGAAACCCCATCTCCACTAAAAATACAAAATTAGCTGGGCATGGTGGTGCATGCCTGTAATCCCAGCTACTGGGGAGGCTGAGGCAGGAGAATCATTGGAACCTGGGAGGCGGAGTTTCTGGTGAGCTGAGATCACACCATTGCACTCCAATCTGGGCAACAAAAGCAAAATTCTGCCAAAAAAAAAAAATTCTGGACAGAATTTTGCATAGAAAGCCCTTTTTCATCCCCAAATTATAATAATAACATAGTACATTTTCTTTTTGTAATTCCAAGGGATCTATTTTTTGTTTATTTTGACATATAGCTCTTAGGTTCTTTTGGCATTATTTAGTGTGTAAGAGTAAGTAAGGATATATTTTTATAGTTTTCCAAATAATAGCAAATATCCAGAAATAACTTATAAAACAGGTCATCCTTTCACCATAAATGTGAAATGCTACCTTTATCCTATGTATTTGAATATATATACATATATATTCAAGTACATTCTCTCTATATATGTGTGTTTATAATATCATATATATACACACACATATGTGTGTGTGTGTGTGTGTGTGTGTGTTACCTCTTTCAATTCCATAGTGTTTTAAGTAATCTAATTTTGGCATACTGAAAATACTGATAAGAAAAATTCTTATTTTTTCTTTCAAAATTTCCTTTGCATTTATAATACATTAATTTTCCAGATAATCTTTAGAATCAGCTTATCAAACTTTGTTAGAAGTGTATTTTATGTTAATCGAGACAATACTGGAGCTTGTAGAATAATTTCAAGAAGAAAGACTCATCTAACATATTTGAGTATTTTCATGCAAGGGCAGAGTATGTTTCTTCCTTTATTATTCTTTGCTCTCCTAAAGTAAAGATTTATAAGTGGTTTATAATCCTTTTTTACTTTATATTAAGTTTATCTCTAGTTTTTTATAGTTTTTGCTATTATTATGGCTATAATTGTCTTTAATTGCTATTTTTAATTGAATGTTAATGTGTTAAAGGAAAACCATTAATTTTTGTATACTGATTTGTGTCCTGTTAAGTTCATAAACTAGATTACTACTTCTAAAAGTTTTATTTGATTGTTTTGACCTTTAGGTATAGAAAATCACATTGCTTGCACCTTACTGCAAGTTCACAAATCCTTTCCATTACTTATACTTTGTAATTTTTTATCTGTATTTAAATTAAGTAGTCCAGTGCAGTCAATATTGAATAAATGGTACTAGTTATAGCAGGCCAGTTTTACTTTTATTATGAATTCTTACAGTATTATCAAAGACGTTCTTTATCAAGTGAGGCAGTTTTTCCTATTTGCAGTTTGTCAAGAGTACATTTTTAGTTTTACTATAAATTTGCGTTGAGTATTATCAAATGACTTCTTTCATATAGTGTCATTATCATGTATTTTCTCCTTTCACATTATCAAGTAGAGAATTACATCAACAGTTGTCCTAATTCATATCATCCTGAACAAATTCTACTTAATCATGGCACACTAAATATGTAATTTTTTATAATAGTTTGGAATCTGAATTTATATTTTTGAATAAAATTTGTCGATAGTTTTTATTTTTCTATATGTGATTAATTTGCATTTTTGGAACAATGATTTTGCCAGGTTCATAGACTGAGTGAGAAAGGTCCATTAAAACTAGATTCATGTATTATTTGCTGATTAATAAATTAAATAACTTAGAAGTTATCTGTTAATAAATAGGTTACTAAAACTTGCCAGTGAAATTAGAGCTAAATTTTTATTTTTGTGTCAAGTATTTGCCGAAATTCACTTTCTAAATTGTTATTGAACTATTTAAATTTTCTACCTATTCTTGAATCAAATTTTGTAATGTATATTTTTGTCAGTCTTACATGTTGTTATAATTTTTAATATTATTTCATGCATTTGCATAAAATGTCACAAATTTCTAAAATACGTTATGTTCCTGTAATTATGCTCATTATTTCATACTAACAATTTTCATTCTAGTACTCTTTTTTCCCCTTTCATCAGACTTAACAAAAGAGTTGTCTATTTTATGAATCTTTGCAAATAAGTAGCCCTTGATTTTTATTTTTAAGCCTATTTTTTATATGGAATTGTAATTGGAAGATTTAAAAAGTCAATATTGCACTGGAAAATATAAAATAAAAATTAATGTTTAATTCTATGTGT


Human Genome Map 3p12 (1921 bp sequence)
14A 

TTCTCTTTTCTCCACATCCTCACCAACATGTTATTTTTTGTCTGTTTAATAATAGCCATTCTAACTGATGTATGATTATATCTTATTTTGGTTTTAATTTGCATTTTTCTGATTAGTAATGTTGAGCATTTTTAATATGCCTCTGGGCTATTTATATTTCTTCTTTTAAAAATGTCTATTCATGTTCTTTGCCGACTTTCTAATGGATGATGGAATGCTAAAGGCCCAGACTTAACCACTATGCAATATAGCCATGTAACAAAAGTGTACTTGTACCTCTTAAATTTATGCAAATAAAAACTCAAAAAAAAAAAAACAAAAAAACCTAAGATGACTAAATGTCAGAAAACCAGGTTTTACATGCCACTTCATTTGCTGAAATACAACGTACACAGCCTGTTAAAATGAAGTCGTCTGCCCCCCAAAATATATAATATTAATAAGGTCTCTACCTAGAATCACCAGTTTACAATAAATGCAGAGGATAGATGCACATGTTAGAAAACACCATAAAGGTGAAATCACCCAAAGTCTACTCGACAAATTATCCAACTTCTTCAACCATTAAATAGCATAAAAGTTAGGGGAGGGGAATCTGTTACAGAACAAAGGAGATAATAATATATCATGCAAACACAAACCCATCTATATCTTGATTCAAATATAAATTGCAAAAAACGGCTTGAAATTACTATAGAAATTTCAACAGAAACAAGGTCTTAGATAAACAGTCCCCAACTTTTTTGGTACCAGGGACCAGTTTTGTGGGAGACAATTTGTCCACAGACAAAGGGTGGAGAGGTGGGGATGGCTTCAGGATGAAACTGTTCCACCTTAAATCATCAGGCATTAGTTAGATTCTCAGAAGGAGTACACAACACAAATCCCTCACATGTGCAGTTCACAATAGAGTTCATGCTCCTACGAGAATCTAATGCTGCTGCCCATCTGACAGGAGGTAGAGCTCAGGCGGTAATGCTTGCTTGCCTGCCACTCACCTCTTGCTGTGTGGCTCCGTTCATAACAGGCCACAGACTGGAACCCATCTGCAGCCCCAGGGTTGGGGACCCCTGTCCTAGATAACATTTAGTAAATACAGTTAATTCTTTTCAGTGCAATAACTTTGTTGAGATTGTTTTTTAACTGGCTAGCCATATACAGAAAACAGAAACTGGACCCCTTCCTTACACCCTATACAAAAATTAACTAATAAAAAAACACTGACATGTAAGACCTAAAACCATAAAAACCCTAGAAGAAAACCTGGGCCATACCATTCAGGACATAGGAAAGGACAAAGGCTTCATGACAAAAACACCAAAAGCAAAGGCAACAAAAGCCAACATTGACAAATGAAATCTAATTAAACTAAAGAGATTCTGCACAGCAAAAGAAACTATCATCAGAGTGAACAGGCAACCTACAGAATGGGAGATAATTTTTGCCATATATTCTTCTGACAAAGGGCTAATCTTTGTCAGAATCTACAAGGAACTTAAAAAAATTTACAAGAAAAAAGCAACCCCATCAATAAGTGGGCAAAGGATGTGAACAGACACTTCTCAAAAGAAGACATTTATGCAGCCAACAAACAAATGAAAAAAAGCTCATCATCACAGGTCATTAGAAAAATGCAAATCAAAACTACAATGAGATACCATCTCACACCAGTTAGAACGGTGATCATTAAAAACTCAGGAAACAACAGATGCTGGAGAGGACGTGGAGAAATAGGAACGCTTTTACACTGCTGGTGGGAGTGTACATTAGTACAACCATTTTGGAAGACAGTGTGGCAATTCCTCAAGCATCTAGAAACAGAAATACAATTTGACCCAGCCATCCCATTACTGGGTATATACCCAAAGGATTATAAACCATTCTACTATAAAGGCACATGCACACGTATGTTTATTGTGG


Human Genome Map 9q22-9q31 (2521 bp sequence)
17A 

TTTCAGCCTCCCTTCTACCCCACTCCAGGTACTTCTGCCTCTGTGGAATTCCTGCTGATTCTAAGCCATGATGAGCATGGCTACCCTACCCTCTGATCTTCCCTCCTACCGTGCTGGGCTCCTGTAGGAGGGGATCCCTCTCTTCCTCCTCCACCAAATGTTGTCTCTTTTTGGAACCTTGTCTGAGCACTCTCCCCAGGTGGGATGAGTCACTTCCTCCCTTTGTTCCCAGGCCCCTTTGTTCCTGTTTCCCCTGAGAGGTCTCTGTCTTCTTCACCATGCTGGGAGTAACCTGAGGACAAGGTCAAGGCCGATGATGTCTATGAGCCCAAGAGAGGGTCTGGTGCGTAAAAGCTGTTTGAGAGAGTATGCAGAAGGAATGGACAAATGAAAATTAGAGACTGACTTACAACTGGGGAAACTTCTCGTTGACCCTTTCTGTTCCTAAAGAGAGTGTCACCGGATAGGGGTCAGGAGCCTGGGCTTTCAGTTGCAACAAGAAGACTTCTTTGCTGTGGGCTTTCTGAAAGACAGTTCCTCTCTCTGTGACTCTTCAAAACAGACATGACAATCATGTGTGCCCTGCTTGCCCCTGAGGCTGCGTTGAGAGATATAAAACCATCAGGAAAGTGCTCAGTGGCTGTGCACCTGCAGCCAGCACCTCTGGCCAGTGTTGGAGAGCAAGGAAGGGAAAGCCAAGGGAAGCCAATTCCTGGGAGCTTCTCCTGTCTGGGATGCCAAGGTGGAAATGAACTTGAGACCCAGACCAAACTTGAGGCTCTTTCATAGTCAGGTAATTTGGGCACCCAGGGCATTGAGATCAGTCTGCCATTCACCCTGTGGCTAGCCACACCTACCTTCAGCTTTTTGACACTGGTACAGGGATCGTTGGAGAAGCTCTCGGTGTCTGAAATCTCAATGTCCTCGCCATACAGAACTCCAGTCAGGTCATTCCGCACCTGTCAGCAAAGAGAAAGCAGAGGGTGGGTGTGCTGGGGACCACAGGAAGGGCCAGTTCCGAGGGGTCACCCTGGGGAAGTCAATTGGGCAAAGCGATTTTCTCTACCGACAATGCAAAGTGAGTGGTTTTGTTTTACATTATTAACTAGACCGCCCCACAAAAACTTGAGGATCCCCCAGTCCCACCCTGCAACTGACACATGGATACAAGGAGGCCAGACAGGGAAGGGACTTTCCAAGATTGCCCAGGGAGTTCCTGCAAGAGTCAAGATTAGCACCTTTGCTGGTGTTTCTCCACCACATCACACTGTCTCCAAATCAGGCTATTCAATTGTGTCTTTGTTAATATTTTGCACTATTTATTTGCAACATTATTTCACTTTTATGGTGAGGAAATAGCTAAGATATTCAAAGACAATATAGAGTAAAGGAAAGAGGAAAGAAGTATGGAACCTGCCTATGATGTTACACGTAACTATGTGTCTACTGACACTCAGAATGAGGAATATCTATGGATGTGAAAAGCAAAGGGCTGCAAACTCCAGTGTTACCAGGACCAGCCAGTGTGTGAACTAGCCTGTGTGGAAGGATATTACAGAATGATAGGGCTGGGCGTGGGCTCACACCTGTAATCCCAGAGCTTTGGGAGGCCAAGACGGGGGGCTTGCTTGAGCCCAGGAGTTCAAGACTGCAGTGAGCCGTGATCACGCCACCGTACTCCAGCCTGGGTGACAGAACAAGACCTTGTCTCAACAGAACAAAACAAAACAAAAGACAGTAATAGTTGGTTGCTGAGTTAGAATGTGGGTCAAGGGTTACCAGACCTTCTGATTTTTGAGGGGAGAAATCAGAAATTTAGACTTTTAAAATATAAAATCCCCTAATTTTTAAATGTTGATACTATTTTGAATTAAAAAAAAAAGAAAGTAAGGATCAAACCAAATAAACTTAAAGTCTGTATCTGGCCTGTGGCCATGGCTGTGCACCCTCTGACATATAGCAATGGAAACTGGATTTTGGGTTTTAGTAGCAAGAACTAGGCTGGGGTTAGGGGATCCAGCTTCCAGGTCCTGCTCTATCACTGACTTGCTTGTGACCTCAATCTCTCGTGTGATTCTTCCCTTCTCTGGGCCTCAGTTTCTTCCGCTTTAGGAGATGCTTAAAGCACTTCTTGTTCACACCCATTAGCATGGCTATTACCAAAAAGCAAAACCACAAGTGTTGGTGAAGATGTGGAGAAACTGGAACCCTTGTGTGTTGCTGGTGGAAATGTAAAATGCTGCCACTGCTGTGGAAAACAGTATAGCAGCCCCTCAAAAAAATAAATATAAAATTACCACATGATCCAGCAATTCCACTTCTGGGCACGTACCCAAAAGAATTGGAAGCAGGGACTTGACAGACATTTTACACCCATGTTCATAAGAACATTTGTTCACTGCAGCTAAAAGGCAGAAGCAGCCCAAACGTTCACTGCTCGATGAATGGATAAATGAATTGTGGTGTATACAGACAGTGAAATACTATTCAGCCTTCAAAAGGAATAAAATTCTGACACAT


Human Genome Map 3p12.3-12.2 (2641 bp sequence)
19A 

ACATATGATTTTTTCAAATTTTCTAAATAGTAATTATTTCCTAGCTCTGCCTTCTGAAAAGTCCTAGAATTACAACAAGCTGGAAACAATGAACAAATGGAGCCTTCAGACTGTAATCTCTAAATATGATTTTCCTTTTAGTGAAAAGATTTCTTTGGAGAAATAGTTGATTATAGATCTAGGTCAAGACATTTATGAGATGACCCTGGGACATTTTATTTTTGTCAGAAAGCCTGGAAAGTATTAATGTGTCTACACAAAACAAAGGAACCAACTTAAAAGAGCAGTCACTGACCACAGCAGAGATAATTGAAGCATCAAACTGAATAAAAAATATAACTCATTCAAGCAGATGCAATTAGTTATCCTGAAAATGATAAATGCATGAATGTAATCAAACATTAATACTTGGTTTCCTGTGCAATTCCTTTTCAGGGTAATGAAAACTGATGAGTGAGAGTTAAATGAAGGACTCCAGAAAGAATGACAGTTACAATATAATGATTTGTGCCCCCCAAATAAAATAATTGATCTACACAAAATACATCAGTATTAGGCAAAACTAGATGGTAAAAAAATTTCGAAGAAAAATAGATTACGGAGAACAAATCAGAACTCACTGATCAAACTTGATATGACTAATTTATAGTTATTTGAGAAATCCACATGCTGTTTTCCATAGAGGTTGACTATTTTACATTCCAACTAATAATGTATAAGGCATTCTCTTTTCTCCACATCCTCACCAACATGTTATTTTTTGTCTGTTTAATAATAGCCATTCTAACTGATGTATGATTATATCTTATTTTGGTTTTAATTTGCATTTTTCTGATTAGTAATGTTGAGCATTTTTAATATGCCTCTGGGCTATTTATATTTCTTCTTTTAAAAATGTCTATTCATGTTCTTTGCCGACTTTCTAATGGATGATGGAATGCTAAAGGCCCAGACTTAACCACTATGCAATATAGCCATGTAACAAAAGTGTACTTGTACCTCTTAAATTTATGCAAATAAAAACTCAAAAAAAAAAAAACAAAAAAACCTAAGATGACTAAATGTCAGAAAACCAGGTTTTACATGCCACTTCATTTGCTGAAATACAACGTACACAGCCTGTTAAAATGAAGTCGTCTGCCCCCCAAAATATATAATATTAATAAGGTCTCTACCTAGAATCACCAGTTTACAATAAATGCAGAGGATAGATGCACATGTTAGAAAACACCATAAAGGTGAAATCACCCAAAGTCTACTCGACAAATTATCCAACTTCTTCAACCATTAAATAGCATAAAAGTTAGGGGAGGGGAATCTGTTACAGAACAAAGGAGATAATAATATATCATGCAAACACAAACCCATCTATATCTTGATTCAAATATCAATTGCAAAAAACGGCTTGAAATTACTATAGAAATTTGAACAGAAACAAGGTCTTAGATAAACAGTCCCCAACTTTTTTGGTACCAGGGACCAGTTTTGTGGGAGACAATTTGTCCACAGACAAAGGGTGGAGAGGTGGGGATGGCTTCAGGATGAAACTGTTCCACCTTAAATCATCAGGCATTAGTTAGATTCTCAGAAGGAGTACACAACACAAATCCCTCACATGTGCAGTTCACAATAGAGTTCATGCTCCTACGAGAATCTAATGCTGCTGCCCATCTGACAGGAGGTAGAGCTCAGGCGGTAATGCTTGCTTGCCTGCCACTCACCTCTTGCTGTGTGGCTCCGTTCATAACAGGCCACAGACTGGAACCCATCTGCAGCCCCAGGGTTGGGGACCCCTGTCCTAGATAACATTTAGTAAATACAGTTAATTCTTTTCAGTGCAATAACTTTGTTGAGATTGTTTTTTAACTGGCTAGCCATATACAGAAAACAGAAACTGGACCCCTTCCTTACACCCTATACAAAAATTAACTAATAAAAAAACACTGACATGTAAGACCTAAAACCATAAAAACCCTAGAAGAAAACCTGGGCCATACCATTCAGGACATAGGAAAGGACAAAGGCTTCATGACAAAAACACCAAAAGCAAAGGCAACAAAAGCCAACATTGACAAATGAAATCTAATTAAACTAAAGAGATTCTGCACAGCAAAAGAAACTATCATCAGAGTGAACAGGCAACCTACAGAATGGGAGATAATTTTTGCCATATATTCTTCTGACAAAGGGCTAATATCCAGAATCTACAAGGAACTTAAAAAAATTTACAAGAAAAAAGCAACCCCATCAATAAGTGGGCAAAGGATGTGAACAGACACTTCTCAAAAGAAGACATTTATGCAGCCAACAAACAAATGAAAAAAAGCTCATCATCACAGGTCATTAGAAAAATGCAAATCAAAACTACAATGAGATACCATCTCACACCAGTTAGAACGGTGATCATTAAAAACTCAGGAAACAACAGATGCTGGAGAGGACGTGGAGAAATAGGAACGCTTTTACACTGCTGGTGGGAGTGTACATTAGTACAACCATTTTGGAAGACAGTGTGGCAATTCCTCAAGCATCTAGAAGCAGAAATACAATTTGACCCAGCCATCCCATTACTGGGTATATACCCAAAGGATTATAAACCATTCTACTATAAAGGCACATGCACACGTATGTTTATTGTGG


Human Genome Map 14q22.1 (2341 bp sequence)
20A 

AGGCCTCAGTGTCCTAGACTAGCACAGAACAAGCAGATGAAACAAAGTTTATATCAGAATGTCAACTGAAAAAGTATCATTTACCATAAATGGAAGATAATTGTATTAAATTCTAGTTAGATGCTAAAACTTCAAGAACTTTTAGAGTCTGTACCTGCATTCTGTTAAAAATATAGATTAAAAAATGCTAACATGTTAACACAAAGGACTTTCCAGAAAGACTTAAAGAAAAGTGAAAGGGGAATAACTGTCTTGCAATGTAATTCATTGTCGTTTAAGACTGGGTCTATGGAACACCCTAAATCACCTGGTTCCATCACGTTCTTTTTAACATGGAGATGGATAGTTTTTCCCCATACTCTATATATTGAGCATTCTATAGTTCATGATTTTTCTGCATAGAGAATTGTTCAAGCCGGGGGTGCAGGCTCACCGACTGGATAGTGAATCAAGAAAATAGTGTGTTCATTAGTTCATCATTACCCTGAGTTTCCAACAAGAATTTAGTACAGGAAAGTAGACAGCGGAGCTGGGAGCCATCTATTTGAAACTGTCTTAAGCAAAACTAAGAAACCGAGTAAGCTTGCTTTTGGTGTCTTTCATCCCTTCTTGTGTGCCCCCTAATTATTCACTCCCCAATGCCCAGACATTATGATGCCTTCTCCTGCTCAGAGACCTTTCTGGGAGGAAGACCTACTCAGACCTGGTATTCCCTCATCCTAGGCTCTACCCTATTTTTCATCCAGCTGTTAAAGCTGAGTGACTAATTTCACACTTATGTACGAATGACCCATAACTGGCTTAATGCTGTGACCATCTTGGGGGTATTCAAAGCTGATAAACACTTTTTTAAGTTATATAATAATCAAAGAAGCTTATCTTTCTGCTTTATTTCAAATTTCACCCCACAGGCCTTACTTATTTTTAAGATCAATGATTTTGATGGGCCCCCCCTTCCCACTCTTAATTCAGGGTATTTCTGGCCCCATCCGGATCCAAACTCTAATGCTCATCTCTTCCATACTGTCCTTTGCAGGTCATCGGTATTGCAAGAGTTGCATAAGGCCCAATTCAGTCTCTGCCCCAAAAGCTCAAGTCCAAACTTCAGAATCTGGGAGGACAAGGATTCAGGAAATTTTGTCAGAACTATGAGCTTTGAACTTTCACTTTTATGGTGAGGGTCACATTTGGTCTGAATCAATTAATCCATTACCCGCCCCCCCCCCCCCCCCCACCACCACCATGTGTGAATTCAAAATAATCAACTTGGGTTTATTATAAAAAACAAAATATATTAATATAAGTATACTAAGATTTTTCTAGAAAACTTGGCCGGGCGCGGTGGCTCACGCCTGTAATCCCAGCACTTTGGGAGACCGAGGAGGGCGGATCACAAGGTCAGGAGATCGAGACCATCCTGGCTAACACGGTGAAACCCCATCTGTACTAAAAATACAAAAAATTAGCCGGGCGTGGTGGCGGGCGCCTGTAGTCCCAGCTACTCGGGAGGCTGAGGCGGGAGAATGGCGTGAACCCGGGAGGCGGAGCTTGCAGTGAGCCCAGATCGCGCCACTGCACTCTGCCTGGGTAACAGAGTGAGACCCTGTCTCAAAAAACAACAAACAAATAAACTTAGAAGAATATATGTGACTATTGGCCGGGCGCGGTGGCTCACGCCTGTAATCCCAGCCCTTTGGGAGGCCGAGGCGGGCAGATCACGAGGTCAGGAGATAGAGACCATCCTGGCTAACATGGTGAAACCCTGTCTCTACTAAAAATAAAAAAATAAAAAATAAAAAATGCGAGGTGGCGGGCGCCTGTAGTCCCAGCTATTCAGGAGGCTGAAGCAGGAGAATGGCGTGAACCCGGGAGGCGGAGCTTGCAGTGAGCCGAGATCGCGCCACTGCACTCCAGCCTGGGCAACAGAGTGAGACTCCGTCTCAAAAAAAAAAAAAAAAGAAGAAGAAGAAAGAAAAAGAAAAGAAAAAGGAAAAAGAAAACTTAATTCTGGCAATGGACTGTTTCTAAAATAATATATTAATACTACTTAATGAGGAAGAAAAAACCTCTGACATCCTAAAATGCCAAGTGTTTGCCTTTACCAAGGTTTAAGCACACATAAACACGCATATTCAAATACCACCCAAAGTGGAGGTGCAAAGATCAGCCTGTACCGCACAGTAACACAGACTGGGTTGTTTTTTGTAAAGAAGGCAACTAGTCCAGTGAGTAATCCCTTCATTTTCCACACACATACCCTTCTGTTTTCTCCCTCTCCTCCCCCCACACCCTCCACTGCAGTTAAAACGTAATTCGAAGAAGCCTAAGGTAAAAGCCCCT


Human Genome Map 8q24.13  (2100bp sequence)
21A 

GTGGACAGGGAAATCTTACCTTCCTGCCTCTCTATGTTCAGGCTGAGTGGGTCAGAAGGAGAGTGTATTAGGTAAGAAAATTTATCAGTATTATTTAGTGAACACTGGATTTATCCTTTTGCATTCTGGCTGTAGTACCCAACTTCCACATGGCAATGCACCCTCACCTCAGCCCTCCGCCCACGTGGTCCCCTTGCTGAGCACTTTAATGAATGACTGCATCTCATTTTCACAGCTATTTGATGCACCTGCTATTATTACTCTTATTACCATTTTCCAGTGGGAAGCTGCTTCTTGGGCAGGGTGGATTTCCATCTGCGTCTCCTTTTCGGTGTTGAAAGCTGGTAAGTGAGGACACCAGGATTGGAACCTGGGTAGTCTGAGTCCAGAATCTCTATTTTCAAGTCTTCCTGCTCTCTGCTTCTGGCAAGTTTGATGTCCACTTTTGATCTTCACCTACATTCCAGCATAATAGCTACTTTTGGTTGTTTTCTCAGCAGCACAAGAGAAGTGTGGCGAGATTTTTAGGTGAGTCATCTAGAGAAGTTAATCTTATTTTGGGAATTCTACTGGCAGCTTCAGGTGGGGAAAATTTTGTTATTTTCTATCCTCCTCTAGGTTCTAAAAGGGAAGAAAGATGGTGAGCGTAGAAAGATGTGACTGTATTCACTATTCACCCTTTGTCGGGTGGTGAGTAAGCAGCTTGCAAAAGCAATGAAGTTTGGAACAATCCAGAGAACCAAACTTTCAGCTGCCAGAGATGGCACCTGGTATCCTGGGTACATCTGCCTGTAGGGCCCAGAAAGAGCTGGAAGCCAAGTGCATGGATCAGGTCTGTAGGAAGGTGGGAGAGCCAGGAATCGAGTGTCAGGGGGCATTTATTACCCATGGAAGCAGGTTTTTGTCAATTTTGTTCACTGCTGGATCACTAACACCTGGACTGGTGCCTGGCCAGGTGGTGGCTTCATAATCATTTGTTGAGTGAATCAATGAATGAATGAATGAACAGCTGTAGCAGATGCTAGCAGGGCTTCCTATTTCTTCCATCACCATAAAGGTGAAAGACATCATAAACGGGAATTTAGACAATCCTCAGAAATTTTCAACTGCCATGTATCTTGACTTGATGCTTCTAGTAGTTATATTTATTTGTAATTCAATCTTTCTTTTTAAATAGTTGACCAAGTGTGGTGGCTCACGTAGTCCCAGCACTTTGGGAGGCTGAGGCAGGAGGATCACTTGAGCCCAGGAATTTGAGACCAGCTTGGGCAACATAGTGAGACCTCATCTCTTAAAAAAAAAAATTAGCTGGGTGTGGTAGTGCACACCTGTGGTCCCAGCTACTTTAGAGGCTGAGGTAGAGGATTGCTTGAGCCTGGGAAGTTGGGGCTGTAGTGAGCTTTGATTGCATCACTGCACTCCAGCCTGGGTGACAGAGCAAGACCCTGTCTCTAAAAAATTAAATAAATAATAAAAAAATTAAAAAGTAACTCCCTTTTCTTTATTTTCAGGCTTCCTTCCCACCTGCTAATTCAAACACTTTACAACCAAAAATATCTTACCTTGATCCTGTTTCTTTCTCTATAACCTCTCTATTTCTGTTTCTTTCAACCAAATTTCTTAGGTCATCTATAATTTTGTTTCTACTTTTTCTATGCATGCCTCAATCCATTGCCAACTCCTCAACCTGCCCCAAGTGCCCACAACTCCACCAAAAGTAATTCTAACATTTTACCAATCCAATACATCACAGTTTTTTATAAAAAACTTAAGAAATATACTTTAGTTGAATTTGAAAGAGTTGCCCACTTGTGTTAAATATTTCTTTCCTTGTGTCTGGGATATCATTTGATTCTGATTCTATTCCTAATTCTCTGACCACCCTTTCTTCGTAGATTTCTCTTCCTTTGTTCAGCCTTTCACATCCTTGGAGTTCCATCCTCTGGTGATTGTTTGTCCTGTTCCATACATTCTCCTTATATGAGCATTGTGTTTTAGCTTATGAATGGTCACATGACCTAGCCAGGCCAATCAGAGTCTTCCATGAGACTTTTGTTTATTTATTTATTTATTAATTTATTTATTCTTCCACATGCCATC


Human Genome Map 6q16-q21   (2100 bp sequence)
22A 

GCACACCTGAGCAAGGGAGAGGAAAGGGTTCTTATTCCTGACACAGGTAGCCCCTACTGTTGTGTTGTTCCCCTGTTGGCTAGGGCTGGAACGCACAGTCAAAGCTAATTCCGATTGGCTATTTTAAAGAGAGCAGGCGTAGGAGCCAGAATGGTGGGGCGAGTAGTTTGGCGGGAAGGTCAGTTACAGAACAGGTGACTCAGGATGACTCAGGTCAGAGCAGGTGACCAGGGGTGTCTCAGGATGGAGCAGGTGACCAGGGGTGACACAGGATGGAGCAGGTGATAGAGGCTAGGAGGGGGTTGTTTACTGAAACTAGGGGCAAGGAGATGACGAGAACGAGAAAGTTAAACTTTAAAATGAAGAACAAAGAACAGGGGAGCTGAACATACTGATAGAACTCTTTCAAGTCTACTTAGGTAACTATTTGTTTGTTTTTCTGCTTCTAAAATTTTGTTGAAATTTTCTCCTTTCTTATTCTCATTGTTCTTGAGGTTTCGTGTATTTAAAAAATCTTCTTACTGTAATTGTCATAGTTGAGTAGGGAGCAACGTTAGATTAATATATTCAATACTTCACTGTTACCTGGAATAAGAGCCCTCTCTTTAAACAAAATATTATGCAGAAATCTAATACAGGAAGCAAATAAAAACTAGAACTACTCTGGTTCAAATAGAGTGAAGACAGAGCAGATCTTGTTCTTGTAATTGAAAGGAATATGATATAATAAGTATTGACAATATTTTCTTCTCACCAAATAAGTTTCTAATTCTATATATAAAGGAAATACTTTCAGAATAAAACGAATATATGAGTTTTATTTTTAAATCACAAAACGAAGTTCAAGAACATTTTTGAAACTGGGAAGATTCATATTTTAGTATCTGTCAAATGATGATAAATTCGGAAGCCAGTGTAATTTATACCCTAGGGGCTGAGGTCTAATTCAACATATTCCAGTTTCTATTTTCTAAAGCTAAAGAAACATGTGTTACAATGTAGATAGGGAATACTTTCTTAATGAACCATGCTGAACTGTAAGATTTTTAAGACTCCTTTTTAATGCATTACATTACACTGTATCTTGTTTTCACATTTATGGTGAGGTTAATATAAAAGAGACATTAAACAAATATATTTCTGCTCTTTACAAAGGATGATTATTGTTTTCTTACATTTCAACTAAAAATTTCTATAATATTATACTTGCAAGAAGTATAACACTCTTAATGAGCAATACAGTTAACCTTAAGGTTAACTTGCAAAATTTCATGTCTAATTTAGTATCATTAACACATTGAAAAATCTCTCCTAAATTTCACTCATCTTGATCAAAATCCATGTTAAAGGTTTTGAAACTACACTTAATACATCTGCCTTATTTTATGCCCCCACTTATACTACTAGTTATTAATGCACTTTGGACAGCTGGTTCCTCTGCCTTTTGAGGATTCTGTGGTGTAACTGATTGGTTCTCAGCTTTTTCCACTGCCCATTTGGGATGCAACCCTTTCAAGTCTGCTTAGGTAACTATAATTTGTTCATGTGTTTTTCTACTTCTAAAATTTGGCTGACATTTTCTCCTTATTCTTGTTGTTCTTGAGGTTTTATGCACTTAAAAAATCTTTCTACTGTAATTATCGTAGTTGAGTAGGGAGCAACATTAGATTCATGTATTCAATACTTCACTGTTACCTGGAATAAGAGCCTTTTTTAAGGGCTCTTACTGAAAAACACAATACACTTATGTTCTTCTATAATGTTTTAAGGAATTTTTTAACATTAATCTCCTGTCTCAGCCTTTAAGGCCATTAAATGACTTAAGATAGTTGCTGTGGCTCCAAACATTGTATCCACATTTCCACAAGAGGAAGTAAAAAAAGAAAAAAATGAGTCTTGCCCTTTCCTTGTAAAGACAATTTCCACAAGGTTCACATTCCTCTGGTCAGAAATCAGTCATACACCCACGCCTAGCTGGAAGGTAGGTTGGGTAATGTGGACTTTAATTCAGACAGTATTGTGTCAGTAAAAGTCAGGGGTTCTATTATTAAAGAAGCAGTGTAAACAGACATGAGAAAACAAACTGTAGCCTCTGTGCTT


Human Genome Map Xq21.3  (2160 bp sequence)
23A

TAAATATAAATAAATCATCACTAGGTATGTTCTAATAAAATTTCAGAACACCAAAGATGAAGATAAATTTTAAAAGTAGCCAGAGGAAAAAGGTGAATTACCTTTAAAAGGTTACAGTTTAACAAGAAGTCTGACTTTTCAACTACAAAGATTTTCAGAATAATATCTTCAGTATGCTGAATGAAGTTTTAAAAAGCAGCTGCCAAAAGAGTTTTAAACTGTGTAGGTATATTTCAAGACTAAAGGGAAATGAAAACATCTGTAGATAATGAAATCTGTAATTACCAACTGAACTTAAGGATATGCTTCAAGTGGAAGAAAAGTTAATTGAAATGGAGGGTCTGAGATGTAAGACAGAATGAGAAATAAATAAAATAGCAAATATATAAATAAATCTAGATGAACACTGACTGCAAAAAGCTACAATAATGATAACATCTTTGGGCATTTAAAAAGATAATTAAAATAAGTAAGAGGAGTGACAAATTCTTTGGGAGGTGATTAAATTGAATGAAAGTATTCTAAGGTACTTGCACAGTCACAAGGGGTGAAAAAAGGTTTTGTTTATAGTAAGACTTTGTCAAGTAGGCATATGTAATTTCCAGGTTACCCATAAAAAATTCAAAACAGAAAGTGAAATTTCCAAATTAGTAGAGAAAAAAGTGACATAATAAAAATTATTCAATTCAAAATGAGGCAAAAAAGAAGAGACAAAGAAACACGGGATATTTAGTACAAATAAAAATCACAACATAATACAGTTCATTAAAACTCAAATTTGTTAGTAATTCCATTGATTATATATGAATTATGTGTTCCATTTATGAAGCAAAATATCAGAAGAAAAATCCACCCATATACTGTTTTAAAGAAACATTTAAGACATAAGAATACAGAAAGTTTGGCAGTAAATGAATCAAAAATGAAATAATGAGTCTACGTGAGGATAATCAATGTTCACTGAAATATTAGGGTGAAATGCTGATGGAGAACTTTATAATAGATATACCAGGTCTATAACACCTGAGCCTAATCAAATGTAACACCATAAAAGTGAAATATCCAGACGCTATCCACCAGATTATAGGAAATGCAAAGCAAAAGAATATTAAAAGACATAAAGATGACTCAATCTTGCAATTCCAGAACATGGGAACTTCTAAAAGATAAATTGTTTTAATCAATATGTAGTAAAAAGGGAAAGGGAACTGTTATTGAATAAAAGTGACATCGTGACCAAATGTAATGTAATAACTTTGGACACTGCTTGAAGAAACCAACTATAAAAATTCATATTGAGTCAGTCAAGAACATGTTTATATTGACTGGAATTTTATTACTTTAAGGATTAGTATTAATTTTTCAGTGTAGTAATGGATTGTAGTTATAGTAAAAAAAAAGTTCTTATTTTTGAAATTTACATTGAATTATTGATGAATAAAATTATGTGATATTTGGAATTTTCTTTAACATAATTTTCATTATTAATAATAAAATCATGAAAAGGAACAACTCTTGTTGAATGCACATTGGAACTCTGTTGAAGCAGGCATTTCTGACCTAGGGGGAAAAAAAACATAAAAGAGAAGATTTTTATGTGATAAATACAGGTGGTTGCCAGGGGCTGCCGGGTGGGGAAAATGGGGAGATGTTAGTCAAATGGTACAAAGTTTCAGTTGTGCAGGATGAGTAAATAAGCTCTGGAGATCCAGTGTACAACATGATGACTATAGTTAATAATACTGTATTATATACTTAAAATTTTCTGAGTAGGTTTGAAACGTTCTCGCCATACACACACAGAAAAGGGTAACTGTGAGGTGATGAATACGTATTCAAGCTAATCACGTAATTAGCTCGATTGTGGTATTTATTTCACAATGTATAAGTAAATTAATAAATCACATTGTACTCAACTATATATATTTTTTGTCAATTATACCTCAATAAAGCTGGGGAAAATGTAAAAATAAATAAATAAATTACCGAAAAAACCCAAACATCCATAAATGAAAATGATACCAAATCTGGCGCCACTTTTTACAATGGATGTAAAAGTCAAGAGTTAAAATCTTTAACATGCATGCTTACTATGTCGAAAGATCACGTACATGAAAACAAACATACTTTATTGTGATTTTTTTGAATGTAAGCGATGAA


Human Genome Map 12q21 (2219 bp sequence)
24A 

TATAATTATTAACTGAAGTCATAGTTTACATTAAGGCTTACACTTTGTGTTGAATAGTTCTATGGATGAGGGAAGGGGCTAAAATGCATAATTTTATGCATTCACCATTAAAATATCATGGGGAATAGTTTTACTGTCTTAAAAATTTCCTTCATTTCAATTATTTGTTCTTCTCTCCACTCTCTAAAGCCCTGGAAACCACTTATCATTTTATTGTCTCTATATTTCTGTCTTTTTCAGAGCGTCATGTAGCTGGACTTATACAGCAAGTAGCCTCTTCAGATTGGCTTCTTTAACTTAGTAATATTCATGTAACATTGCTCCATGTGTTTTCGTGGCTTAATAGGTCATTCCTTTTCATTACTGATCATTTTATTCTGTGCATGTACCACAATTTGTTCGTCTACTACTGAATGATGTCTTGATTGTTTCGGTTGTTGGTGATTATGAATAAACTTGCTATAAACATTTACTTGTGTGGATGTAAGTTTTCAACTTATTCAGATAATATTTAAAAGAGCAATTGCTGTATAGTATGGTAAGATTATGTTTAGCCTTGTATGGAACTGCCAAAGTGGCTGTACCATTTTGTATTCCTACCAGCAATGAATGAAAACACCTGTTGATCTGCATCCTTACCACTATATGATATTGTCATATTTCAGATTTTAATCCGTCTAATAGATGTGTAGTGGTAGATAGTTGCTTAATTTTCAATTCTCTTATGACATACAATGTTTAACATCTTTTTATATGTATATTTGCTATCTGTATATCCTCTTTGGTGAGGTGTCTGTTCAGATCTTTTTCCCATTTTAAATTGGATTGTTTTCTTATTTTTGAGTTTTAAGTGTTCTTTTTATATTTTAAGTGCAAGCCCTTTATCAGATATGTATTTTGTGCATATTTTCCCACTCTGTGGCTTGTATTTTAATTCTCTTAATAATATCTTTGCAGAAGTTTTTAATTTGAACAAATTTCACTTTTATGGTGTGCTTAAGAAGTTGTATCTAAAAACACAAGGTCACCTATATTTTCTCCTGTTACAGAAGTTTTAGACTGTGGGTTTTTTATTTAGCTCTATGATCCATTTTGAGCTAATTTTTGTGAACTGTGTAAAGTCTATGTCTGGATTCTTTTTTTTTCCAATGTAGATATCCAGTTGTTCCAGCATCACTTGTTGAAAAGATTATCTTTTCTACAGTGAATTGCATTTGTTTCTTTGTCTAAGATCAGTTTACTATATTTGTGTGGGTCTATTTCTAGGCTCTCTATTCTGTTCTATTGGTCTATGTGTTAATTCTTCCATAACATGCTGTTTTGACTATTGCAGCTTTATAGTAAATTTTCCATTTGAATTGTGTCATATTCTTCTTTGTTCTTCTTCTTGTGTATTATGTTGCCTATTCTGAGTCTTTTTGTATTTTAATATAAACTTTCTTGTCAATTTGTTGATACACAGAAAATAACTTGCTTGGATTTTAATGGGAGTTGCAATGAATGTGGAAATTAAGTTGAGAAGAATTGACATCTTAGCAATATAGAGTCTTTCCTGTCCCTATACATAGAATATCTATCTAGATCTTCTTTGATCTCCTTCATCAGACTTTTGTAGTTTTAGCCACATAGATCCTGTACATATTTTGTTTGATCTATACTTAAATATTTTATGTAATCAATTGACTTTTGTATATTAACTTTTTATCCTACAACCTTGCTATAACAGCTTATTAGTTTCAGGGACTTTTTGCCAACTATGGGATTTTCTGCATATAAATCATGCAAAATATGCAATCATGTCATCACCAAACAAATATAGTTCTATCTATGCCTTCCCAATATGTACACCCTTTATTTCTTTTTCTTGTCTTATTGCATTGGCCAGGCCTTCCAGTACAATGTTGAAAAGGAATGGTGAGATACAATATTCTTGCCTCTTTTTTCATTTTACGAGGAAAGCATTCCTTTTAATAGTAGGCAGTCAGAATATAATATGTAATATTTTTAAAGGCAATAAATAGACATCTAAGTGAGTTATTTTAAAATTGAGAGTTTAAAATCAAATAAAACTAAAAGAATTTAATAGTTTGCCTCTGTATCATGGAATGAAGGAGTTAACAAGAGCTGATGAGAGAATCTGCTATTTGTCACTATATCTTTTATTAGCATTTGACTTTTAAAATATGTTACAATGAATATTTTAATAATTTTCTTCATAA

Human Genome Map 7q22 (2160 bp sequence)
27A 

ACATCTGTCTGTTTTGTGTGGCCGTCACAAAATAATCAAAGACTAAGTAATTTATAAAGAACAAAAATTTATTTCTCATAGTTAATGGAGGCTGAGAAGTCCAAGACGAAAGTGCTGGCATCTCATGAGGGTCTTCTTCCTGTGTCCCCACAGGGCATAAGAGTGTAAGACTATGAACTCATTTCTGCAAGCCCTTTATACAATGATGTTAATTCATTCATGAGAGTGGGGACCTCGTGACCTAAACACCTCCCATTAGATTCTACCTCCCAACACTGTTGCACTGTGCATTGAGTTTCTAACACATAAATTTTGGGGGGCACATTCAAACCATAACATGGAGTTTTCTGCATTGAGAAATGAAGGATCCATTTAATACAGGGACCTCAAAATACAAAGAGAAAACTGACTGGCTGTATGGAGCTAGACGAAGAGGAGTAAGAAACTACTATTTGCAAGGCTGTGTAATTCCAAGGACTGTTATTCTTGGATGCTATGATGTTTTTAAAGAACAAACTACTATACATTTGTAAGTTATTAAATTATTAATATTATTTGAGAATTTCAAATGGCTTAACTAATCAATGTGACATAGTGGGAAAATTGGGCCTTTAATTGAAGACACAATTTGCTGATTACCACTTGGTAACTTAGCCCTTGCTTCTCTAACCCTTAGTTCATCTTGTAAAATGCATTAGTTCTACTCCATAGGGGTATTGTGAGATTTAAACGCAGTGCAGCATATTAAGCACCCAGTGTAGTCCCTGATACATAGTGAAACATCAATAATAAATTGTTGCTACTGGTAGAAATCCCTTGGCGTTTGGTAGATTTCCAATAAATACTAATTCTTCTAAAACTTTTAATGATTATGTAGATAGATATATGCCTAGATCTGGTAACAAATATGCTATATCAATAGTCAAAACATTCTCTCTTAATTTTATTATGATATATATTGGAAATCTTAGTGTGGTTTTGATTATACTAACATAATTATGTGGCATTAGTATGCCAAATGTACTCACAGTTATGCCAAAATTACCTGCCCCAAATTACAGCTAATCCTTTCTTTGGTCCTAGGAGAGATACGCACACTAGGGATATCACCATAAAAGTGAAGAAACACTTTATTACTGGCTGGGCTTGTTTCTGAAATTCTAACACAGAGTTCTTATAACATGGACTTTTCCTTGCCTCCTAGTTCAAGCTTGAGGGCTTACTGTGCTCTTGCAGGGAAAGATAAAAGAAAGTGTCAGAGTGAAAGAATGGTCAAATGTATGAACTCTTCTTTTATTTATTAATTTAAATACAGTGACTCTGTTCACTAGTAAACACACCTAACCCCTGCCTTAGAGTCAGATTAACATCTTCTTTGAGGACAGCCCAAAGAAGAAAAATGCAAGGATGAAGCCTAGAGAGGTTTCCATCTCGTATACTTATATTCCACTATCTTTGGTTCTTTCTTTCAACCATTAGACTTAAACCCAACTGTATAATTAATCAAACATGTGGATATTTCCTTGGAGGAAGAAATAGAGAAGTGTCAGGGAAGTTCGACCGCCACCTAAGTGTGTCTGCTTTTTTAATGCTGCCTTATGGTCTAAAGAGATGGGTGAAAAGCAGAGTATTCATTTCAAGGCCATACTATATTATATGCCATCTATTCACTCCAGGCTGCTTGTTGTCAAGGAAGAATAAAAACCTTGATATCAAAGAGAATTAAGCTCTCAAAATTAGTTTCTCTTTCACATACCAAAGTAACCTTGAGCTTTCTAGCCTGCAAATTTCTCTCCCTTAATATTCTTTCTCTGTTCCGTTCCACTGAAAGTGATGTCACAGTGGTGTAGTTAGAGTCTGGGTTACTCTTTCCTGCAGAACTGTTCTTCAGTACCTCTAGATAGAAAATAGTCCAACATCAAGTCTTGCATGAGTTTTCCTTTACCAAAGATCTAGTAGTCTAGAAGATATTTAAAAATCACATTATTGAGCCCACATCTGCAAAAAGGAGAAGTATATACAATATTCTTAGGACTCAATACATATTACTTGTGTGCTTGTCTTTGCATGGACATGTATGTGTTTTAATTTCTCTTGCGTAAACACTTAGGGTTGGCATTGCTGACCCACATGGTAAGTGTATGTTTAACTTTATAAGCA


Human genome Map 11p15  (2160 bp sequence)
29A 	(a run of 18 A residues has been considered as a putative Pol III terminator. See Emerson and Roeder (1984) J. Biol. Chem. 259, 7926-7935)

TACTGCTTCATCCTTGAGTTCTACAAAAACACACACACACAAAAACCAACAAAACTTAACTATAGGCTGGGTGAAGTGGCTCATGCCTGTAATCCCAGTGCTGTGGGAAGATCTTTTGAAGCTAGGAGGTTTAAAATCAGCCTGGGCATCAAGGCAAGACCCCATCTCTACAAAAAAAAAAAAAAGCCAGGCATGGTAGTGCACACCTGCAGTCCTAGCTACTCAGAAGGCTGAGGTAGGAGGATCACTTGAACCCAGCAGTTTGAGATTGCAGTAAGCCATGATCACATTACTGCACTCCAGCCTGGCTGACAGAACAAAACACCACCTCTAAAAATAAAAATATAAAATAAATAAAAAAATTTAAAAACCTAAACATAGCTGCACTTTACTCAATATATTTACAGTTCTACATATGTAAAAACTTGTATATTGACTATGTTTTAAATGTGTAGGGGAAGTTTCTCACCTAAAGGAGTCCCATAGTGAACATTTAAGAGCAAATGATTCCTTTTTTATTTGTATTTTTGGTTTTGCCTCTAGCACATCAGGTATTCTTTAAGAAGGCTATGCCTCTGAGGTTGCATGATCATTAACTAATTCATAATTTCCCTTGCATATATTTGGGTATTTTGGTGTTTCAGCCTTTCCCACACTTTTTTTATTTGCATGTCTTCACGATCACCATTATATCTTTGTTCCACCTGTACTATTATTTACTCACTCTTTGTCTTTAAATCAAATCACGTTTCTTACTCAAGTAGATTTAGTTTTAAGACAAACCTTATGGCCGGGCACAGTGGCTCACACCTGTAATCCCAGCACTTTGGGAGGCCAAGGCGGGTGGATCATGAGGTCAGGAGTTTGAGACCAGCCTGGCCAACGTAATGAAACCCCGTCCCTACTAAAAATACAAAAAATTAGCTGGGCGTGGTGGCGGGCACCTGTAATCCCAGCTACTTGGGAGGCTGAGGCAGGAGAATCACTTGAACCCGGGGGGGCAGAGGTTGCAGTGAGCCGAGATCGTGCCACTGCACTCCAGCCCAGGCAACAACGCGAGACTCTGTCTCAAAAAAAGAAAAAAAGGAACTTTATGTCGCTACCATAAATGTGAAATTACTAGAACTCACAATAAATAGAAGTTAGTAAAGACACTGAATTCTAACTAGACGCTATTGCTTGTTGAAGGCTTTGATCTTAGGAGGATTAGAAAGCATTCTAGGCCAGGCACGGTGGCTTCCTGTGTGTAATCCCAGCAGTTGGAGAGGCTGAGGCAGGCGGGTTGCTTGAGCTCAGGAATTTGAGACCAGCCTGGGCAACATGGCAAGACCCTGTCTCTACAAAAACATACAAAACTTAGCCAGGCGTGGTGATGGCCACGTATGGTCCCAGCTACTCAGGTGGCTGAGGCAGGAGGATTGATGAACCTGGGAGGCTAAGGCTCTAGTGAGCCATGATCACACCACTGCACTCCAGCCTGGGTGACAGAGCCACACCCTGTCTCAAAGGAAAAAAAAAAAAAAAAAAGAATTCTAGTGGTGTGGTGTGGAAGACACATTCTCAGCAGACTAAGGTTGTATCTTTATAACCACAAGGATTGAAAAAGAACGGAAGGACAATAACTTTCTCATAAGGTGATTCAATGTTATTTAGTGCTGTTTCTGTGTACCATCAAAAATCCTCTTACTACACACAGAATATTATAACACCATCTCATTGTCCACATGAGCTCAGAAATTGGTCATCAAAGCAGAAAAGTCTTTAAAACATTGATCTCCGGCCGGGCGTGGTGGCTCACACCTGTAATCCCAGCACTTTGGGAGGCTGAGGCGGGCGGATCACAAGGTCAAGAGATCGAGACCATCCTGGCCAACATGGTGAAATCCCATCTCTACTAAAAATACAAAAATTAGCTGGGTGCAGTGGCAGACGCCTGTAATCCCAAGCTACTCGGGAGGCTGAGGCAGGAGAATTGCTTGAACCCAGGAGGCAGAAGTTGCAGTGAGCCGAGATCACGCCACTGCACTCCAGCCTGGGCAACAGAGCCAGACTCCATCTCGAAAAATAAAATAAAATAAAACATTGATCTCCAAGAAAGTAGATCATATCTGCTCTCTATCTGACCACATTGTTAAACTTGGTTATGTTTGCAGGTTAAAG


Human Genome Map Xp21 (2040 bp sequence)
30A (no TATA element downstream of the PSE; the TSS was predicted by moving 50 bp downstream of the end of the PSE and selecting the closest pyrimidine-purine sequence motif).

CAAAGACAGGCCAGTGCTCTACTCCTTGCTTCCTGGGCTCCCCAAAAGGGAGCTGACTCCTCATCCCTCAACCTGGAGAACCAGTTCAGCTCTTCTTTTCACCAGAATCCTTTCCCTGCTTCCGACTCATCTTCTTTTTCTCAAAGCTGTTGTAACTGTATTGTTCTCACCTGCTTTGCCCAAGACAGATTCCCCAGTCCTCCCCATCAGTGTTTGGCATTTATTCTGGGTGTTCTACTAGTAATGCCCAGCCCCGGTCCTGGGCTTCCTGCTGTTTCTATTGCATCTCCCTAACTCTTACATCCACCCCAACTCAGTGTTTTTGGCCTTCCTCAGCAACCAGGAATCTAAACCACCCTCCACCCCATAGCACCCTATGGATGACGGAGCCTTAGTTCTTGATGGTGATGCAGACACCTTGAGGTGTGGCCATGACATTCACTCAGCCCTTGGCCTGGTAGCAGCAATTTTCCCTGATAAGGTCCCCAAACTGACCCTCAGTTGTCCCCTGCAGTCCCATTAGGGCCTGTGGAATTTACGACTTCCATACACAGCACCAGGAAGTTGAGGATGGCTCCACGTGCTAGCTCAGTCTCTTTGCCCTCTCTCTGCCTGTGGCAGATTGTATTTTCCAAAGATGACTGCACCAAAATATTCCACCCCATGTTATCTTCTTAAATGTGAAGTTCACACTAATTCTTCAAGAAATGGGGCCTCTGTTTACACCTGCTGAATCTTGGCAGGCCTATAATTATAGTGGTTATGATTCTAGTGATGCTATATGACTTCTGAGACCATAAAAAGACAATACAGCTTCCACCTGGTCCTATTGGAACAGTCATTCTTGGAACCAAGCCACCATGTTGTGAGAAAACCCAGCCCACATGGGAAGGTCACATGTAGGGATGACAGTCCCCACTGAGCCCCAGCCAATAGCCGGCATCAACTGCAAGACATGTGAGTAAGCGAACCCTCAGATGATTCCAGCCCCCAGCCTTTGAGCTGCCCCAACTGATGCTTTGTGGAACAGAGAAAAGCTGTCCCCATTGAGCTCTGCTCAGATTTCACATTTATGGTAAAAATCTATATGGTCCTTACTTTAAGTTACTAAATTTGGGGATGCTTTCTTTACATAGCAGTAGGTAATTAGAACACTGCCTGATCAAACTGCACTGCAACTTTTACTCGGCTGCTAACTATATGGCTATAGCCGAGCATCATGGGGCCACCGTGTCTGGCAGTCCCCACATCCGAGTTCCAAATGCGGAGCACAAAAGTCCCACTGTCACTGATCTTCCCTTCCACTCTCAGAATCTCAGTCTAGTATGGGGAAGCAAGGGTCGAACCATGTGCTTCCCCCGTCAGGGCAGATGGTTCTCTTCCTGCCTGGAAGGAATTCCCTCTACATAAAAGCCTCTTTCCACCAGGTATGGTGGCTCAAGCCTGTAATCCCAGCACTTTGGGAGGTGAAGTGGGCAGATCACCTGAGGTCAGGAGTTTGAGACCAGCCTGGCCAACATGGTGAAACCCCATCTCTACTAAAAATATAAAAATTAGCTGGGCATGGTGGTGGGCGCCTGTAATTCCAGCTACTCGGGAAGCTGAGGCAGGAGAATCACTTGAACTCGGGAGGCGGAGGTTGCCGTGAGCCGAGATCATGCCACTGCACTCCAGCCTGGGCAACAGAGTGGGACTCCATCTCAAAAAAGTAATAATAACAAAAAATTTTTAAAAAGTGTTCTTCTTCCCAAGAAAGCAGACATCAGACATCTTTCCCCCTTCATTGGGGCCTTAATTGCAGATGGGACTCTGGAAGAGATACTGACATAAGCATAAAAGTAGGTCCAAGAATATTTAACCTCTACATAAGAATTCAAATAAGCTATTGACCTTATGAGAGAGTCACAATGATGGACACCTTCAAAGGAAGGAGAAGCACCATGGAGGGCAGAGGAGAAAGACCATACCTGCTTGACTTGGTGTGAGAGGCATTCTAGATGTTATTGACATCATATAATAGACAGATGACAAGCATAGAAG


Human Genome Map 12q21 (2100 bp sequence)
31A 

TGTACATGCATTCATTTTGCTATCCTGCATTTGTTCTTTCTTCCTCAGACCTTTTCAGTCCCTAAGAAAATGGTATCATTCTGGGTAGGACCTAAGATCAGTGATGAAATAACAGAATGCAGGATGGCAAGACTCTATATGGAGAGGGAAATTTTACAGAATCTAAACCTGGGGATACTAAATTAGATCAATTGAGTATAGGCAATATCAGAGGGTAATAAAATAGTTTAAGAATACATAGATGTTTTTTGTTTGTTGGTTAGTTTTTGCTTTTCTTGTAATTCAGGTTAAAGATGTGACATTTCCTAACAGCACCAAGGAAGGAGCACCAGAGAACATGAAAAGGCACTCAAGCTAAGTGGTCTGTTGAACTTTGCATATCTTCCTCTTTGTCAATGAAAGAAGGCAAAACCAAACCTAAATGAAACAAAACAAAGAACTACTTACAAGGAGTTTTAAAATATCTCAATCTCTGCGTTACTTATGTAACCCACAGAGTATATAGATGACCACTTAGAGGTATTCCTACTATTAACAAAGGTATTAGTATCTGTGTTTTACCCATGAAAAATTAGAAACTTAGAAAAATATCTTGTTCAGTTTCACACTGGTACAATATGAGGGTATGAAAGTTGTAACTGACTCTAAAGCTTGAGCTATTTTCTTATAGATATAATTTAAATAATGTCTATCAACTTTCTTGAAATATTCTCATTGTTACCTAAGAATTTAAAATATTGATATGCAATTGATCTAAGAGAGGTTAAACATGAATTGGAAATTCCTTCTCCGATATCAGGTTTGATTTTTCACAAATATCAATGTTTATGAAAGTATTCCTAAAATTTCAGGTAAAACCACCCAATAATATGACTGTGAAAAATTATGCTTCTTCCTCTATGAGCTACTACATTCTTAAATTATCTGAGCATTCTATTAAAACTTAAAAAAATGCTTAACTTGAGTCTGCATGAATCTGAATTCCCTGCATATTTAATTTTAAAGAAAATAGTTTATTTTTTTGTTAGACCAATACTTTACAAACTTCCCCAACCAATAAGAAAGAACAAAGAGGAGAACATGAATATCCCTGGGTATTTGTGAGTAAATCCCCAAGAGACTAACCATAAATGTGAAATTTCTTTATAATTGTATGTCTTCTTCTAAAATATTCATGTGGATTGTGCATTCTATTCTATCTTCTTATTTTAATAAAATCTGTTTTAAATTATTTACTTCCTGGAAACAAATCTCCCTGTTGTGTTGGTTTATGAACATGGTTCTATTGCCTTCAGTCTATTGTCGGAAATAAAAACAGTCCTGCAGTTGTTGATTGAGTGTACTATGCCTTTAAGAAGTCATGGCACTCATGCAACAGCCATGTAGTTGTTGATTGAGAGTACTGTGTCTTAAAAAAAGAACTTTTGCTAAATAAACTGACTCTGTGAGCAGCCCTTCATCATTTAAGTGAGAAATGTATTGAATTAAGTTACCTTGATATTGCCTTTTGTTATATTTTTATTTCTTTGATACAAAAGAGTAACAATTTAATTCGAAATTTGAAATCCCTGAATTGCCTATCCTCTCCAGTAAGTCACTACACACCTGTATAGGGGAGCAGCCTTTCAGAATATTTTTCCTGAACATGAGAATATAAAGCAGGAGGTGGTCATATTTGTTTGAGTAGCACCTCCTGATACCATTAATCTGAGCAGAAGAGTATGGGTCCATACTAGAACAGGATATGACTAGGAAAATGAAGAAGAAATGAAAGCAAAGTATTCAACAGAAACATCTATGCTTTTTGCCATTAGCTGAATGTGACATAAGAGTATAGAATGATTTGACACGATTCCAAATCTAAATGTAACCAAGGAACTTTAAATATTATTAATGAGCATGGCAAAGTTTAGGGTCAGGGGGAACAAATTTAAAAACTATGAGCATTCTCATGACATGAGTTAAAATGCAAAGACGTAAGTTAAAACATAACTAATGACATTAATAAAGTGATTGAAGCTCTATGTCACTTAAAGATAAGAAGGTATGATAGTTTAGACATTGTTCTAAAGGCCAATCTAAGTGAAAAAAGTTTTCAGG


Human Genome Map 17q21  (2100 bp sequence)
32A (no TATA element downstream of the PSE; the TSS was predicted by moving 50 bp downstream of the end of the PSE and selecting the closest pyrimidine-purine sequence motif).

AGTTAGAATTCTGTGAGGTTTGTATAAAAGGAATAGAGTGGGGGCCCAAAAACCAGTAAGATGAGAAAGTACTGTTTGCTCAGTTCTAGGATCCATGAAATAAATAATAAATAAATAAAAAAGAGAAAGTAGTGTTTCCTGCCACTTTAGAGGAAGGACTCACATATCCTACCTTCCATCAGCCTTGAAGGAGATGAGTGCCCTCTCTCCAACACCTGGTGGCCTTCCCTACCCCTTCCCCAAAGCCTCCAAGAAGGCCCCTGGCCTAGCCTGATGCCCACTATCAGCAGGAACAGGCACGACAAACTTTCCCCTTCCTATCCCTCCCCACCTCTGGAAAGGGCTGGGGACAGCAGATGTGTCCTTGTTAGTTCCATCCATTTCAGCTTTGGCTGGGGAGCTAATTTCACTGGAGCCAGGATAAGCATTAGGGTAAGTAACTATTTTTCCTGTCTTGGGCAGTTTCCTCACTGACAAATGAGGGCAGAGTTCTAAGCTCTCTTCTAATTCTAAAATTCTAATGTAAAAATTGCCAGACTAGTGGTGGCGCAAGCCTGTAATCCCAGATACTCAGGAGGCTTAGGCAGGAGAATCGCTTGAACCCAGGAGGCGGAGGTTGCGGTGAGCTGAGATCGCGCCATTGTACTCCAGCCTGGCAACAAGAGGGAACTCCATCTCAAAAAAAAAAAAAATCACCAGACTAATATTTACCTTGAGTGTTATGCGCATCCATGTGAAGAGACCACCAAACAGGCTTTGTGTGAGCAATAGTTTTTTAATCACCTGGAGTCAGCAAAAGGAGATGGGGTGGGGCAGTTTTATAGGATTTGGGTAGGTAGTGGAAAAATTACAGTTAACGTGCGTTTTCTCTTGTGGGCAGGGGTGGGGGTAACAAGGTGCTTGGTGAGGAGCTCCTGAGACTCATTGTCCAGGAGAAGGAATGTCACAAGATCAATTGATCAGTTAGGGTGGAGCAGGAACAAATCACAATGGTGGAATGTCATCAGTTAAGGCAGGAACTGGCTATTTCACGTTTATGGTTCTTCAGTTGCTTCAGGCCATCTGGATGTATATGTGCAGGTCACAGGGTTATGATGGCTTAGCTTGGGCTCAGGGGCCTGACATTGAGGATTCTTTTTTATCTTCCTCTGATGCTCTTCTATAAGAATGACTCTGTTTTGGAAGAAAACGCAATTAAGATTTTCCATCACAACAACCACTATCTCCAAATCTGTATTCATTCCTTTTAATTCATTATAAGTCTCATCTACCTAATGAGATAACTTTTTTGAAGACAGGAATTGTATGCTGTTTAACAGTGCTTTGTTTCTTCCATAGTTCAGTCATCCTTGATATTTTGCGGGGGACTGGTTCTAGGATACTGCCCCCACACACCAGAATCTGTGAACGCTCAATCCCTTACATATAATGGTGCAGTATTTGAATATAACCAACACACATCCCCCCGCCACCACCCAATTAACTTTTTTACTTTTTTTCCCCCCCGAGACAGAGTCTTGCCCTGTCGCCCAGGCTGGAGTGCAGTGGCACGATCTCGGCTCACTGCAAGCTCTGCCTCCTAGGTTCATGCCATTCTCCTGCCTCAGCCTCCCGAGTAGCTGGGATTACAGGTGCCCGCCACCACACCGGGCTAGTTTTTTTTTTTTTTTTTTTCTTTGTATTTTTAGTAGAGATGGGGTTTCACCATGTTAGCGGGTGGACCTTGTGATCCGCCCACCTTGGCCTCCCAAAGTGCTGGGATTACAGGTGTGAGCCACCATGCCCAGCCAATTTTTGTATTTTTAGTAGAGACGGGGTTTCACCATGTTGGCCAGGCTGGTCTCGAACACCTGACATCAAGTGATCCGCCCACCTTGGCCTCCCAAAGTGCTAGGATTACAGGCATGAGCCACCGCACCCAGCCTCAGACTAAACTATAATAAAAGAGAAAGCAGAGAGAGTAAGAGCACCTCATATGGAATCACCTACATTTCAGAAGCTGGAAAGAAAGAGAGTGGTCTACTTGATGATATGAAGCATGATCAATCAGTATCAATACTAGCTTTAGGGTGAAGGCATAGCCAAATTGGAAACTGTGGA


Human Genome Map 1q32.2 (2100 bp sequence)
33A

AAAAAAAACCTTGTCCAGGCACAGTGGCTCACATCTGTAATCCCAACACTTTGGGAGGCCAAGGTGGGCTGATCACTTGAGGCCAGGATTTTGAAACCAGCCTGGCCGACATTGCAAAACCCCACAAAAACTAGCCGGGTGTGGTGGCACACACCTGTAATTCCAGCTACTTGGGAGGCCGAGGCACAAGAATCACCTGAAGCTGGGAGGCAAAGGTTGCAGTGAACCAAGATCATCCCACTGCACTCCAGCTTGTGACACAGTGAGACTGTCTCAAAAAATAATAACGAAAATAAAATAATCTAAAATTTAAAAAAACCCTAATTCATAGTTATGGAATTATTGAGCATATTAAATAAGATAATGCATGCAAAGTACTTAACAGCATCGGACATATTTTAAGCACTCACTGATGCTTGCTATATAGTTAAATTATATAGCTATATGTATGTGTATATACATGCAAAGATCAGGAGATATGCTGACATAGAATGACTATGGCAGGGTCCTGAAAGAGACCACAAAAGAGAGAAGTTTTCACACTGGTTCTTTCCTTTTGGGGGATGCTGACAGTTCCCCTAGAAGGCAGCAGACTTTGCCTCTGGGAGCAAGCCACTGGCCTGGCCCAGCACTCCTGAGATGAGCAGAAATGGGCAGAGGAGAGCTCAAGACAGAGCACAGGCCAGACTAATGTCTTCCTGAGAGGAGAGCAGTGGGAGGAAAAAGGGGGCAGCAAAGAGACAAGAGATTGCCTCCTTCACCTCCACCAATGTTACCTAAGCTAAAACCCCTCTGTCTACCAAATCAGCCCTGGTCACAAACTAAAACCCAAACCCAACAGGAGGCTTACTTATCCACGCCAATCTGAATTTCTCCATGACATGGACCAGGTGGGACTGTGGGTTTGGTGCCATGTACATGACCTGTGACTTAGTGGATGGAGTTCCTTAGGCCACAGCAGCCTCTGGCTCAATGAAGCTTGATCTACTGAGTACCTGGACCACATGGGGCTCTAGCAGCAGTCCTATCTTGAGCCCAGAACAGTAACTTTCAATAGAATATACACTGGTATGTTGAATCAGAAGTTCAAACGCCCTTCACCTTTATGGTGACTTTTCTCTCAAGGACCTCCACTGCTTTCTTCTACTATGCCTTCGTATCATTGACCATTCCATCAGTGAGGGCCACCACAGTCCCTCAGAATTCTTTAAGACTAACTAGGGGGAGATTAGAGTACCAATCCTTCTAAACCTTTCAAAAGGCTTCTTTTGAACCCTTTTCAAAAGATTTCTTCACTTAGCACCCTGGAACCAAATGGAAGTGAATATTTTTGAGAAGACGTGACATCTTTCTCCTGGGCCTTGCCCAGCCAAAAATGTTCTGTTATCTGTTGCAATTAAAAGAGAGCAAAGAGTAAGAAGTCTCTTTCCTTAAAGTTTCTTTGGCCACTTGAGCGGAGCTTCCCAGAGCAGTAAACTCCTTTAGGATAGGGACTGTTGGAATTAAATGAGCTGGGGAACCACAACCTAGAAACTGGACTTCAGCTTTGTAAACTCCGAAACTCATTATCACTGTGATGGTTAATTTGATGTGTCAACTTGAGTAGGCCATAGGGTGCCCAGATTAAATGTTGTTCTGGGTGTGTCCATGAGGATGTTTCCAGATGAGAATAGCATTTGAATTGGTAGACTCGGCAAAGTAGATTGCCCTCCTCAGTGTGGCTGGGCATTGAAAAGGCCGAAGAAAGAATTTGCCCCTCTTTTCCTGCCTCACTATTGAGCTGGGATATCTCATTTCACCTTCTCCTGCCCTCAAACTGGGATTTACATCATCAGCACCCCTGGCTCTCAGGCCTTTGGACTTGGACCAAACTGCATCACTGACTTTCCTCCATCTCCAGCTTGCAGACACAGATTATTGGACTTCTCAACCTCCATAATCATGTGAGCCAACTACTCATAATAAATAAATAAATAGGCTGGGTGTGGTGGCTCATGCCTGTAATCCCAGCACTTTGGGAGGCCCAGGTGGGCAGATCACGAGGTCAGGAGTTTGAGACCAGCCTGGCCAATATGGTGAAACCCCCATCTCTACTAAAAA


Human Genome Map 5q15 (2040 bp sequence)
34A 

TACAGGATGAAGGTAACAATAAGAGAAAGTGATCATAATAGTCAATATTTAATACATATTTAATATATATATTTTTAGATTTAACAACTGGTTAAATCTATTAACCATATATCATCTAACCATTTCTATACCTTCCTATCACTCTTCTTTTCCTATTCTCTCTTAATTCCAATTTTCCTACAACACACACACTCATATACACATACGCACACATGCACCCACTATCCATAAGACCATCACGTCTGGGGATTTTGCACATACAGAGCCTAATAAAATTCAGCAACAAAGATCATTCAAATTCATAACTCAAAAATTATCAGGTACAGAAATACATGAACTGAGGTGAAGAAAAGCCAAAATACTGGGAGAAGTAGAATATCTTTCACAAAAGACCTTCTAGGAAGATCATATGGTACTGTTCTGCAAATCTTTCCTTTTACAACATTGAATGTTTTAATGTGAGCTTTGCAGATTCAGTTTCAAATCTTATCACTAATCCTGCCCTTAAGCAAAGCTGTAAAGAAGGTGAAATTAATTTTATACATTTCCTATTCTGCATTCTGTCATCCTCATCTTCCTTTGAGGGTCTAACAACTTCCTACCACTTTCTGCTTGTGCCCATTACAACCCAGATTTTCATCTTTTGTACCTGGAACAGGCCTGGCCTTCCACAATGCTCTATGTGCTATGAAAGTCAGTTTCTGCTATTATCATTGTGTTCTATTATTTCATGTATTTTCTAATAGCCTTAAAGATTACTTTGAATAGGCCTGGAATCTCTAACACAATTAAATACTATAGCAGGCATATAGTATTACCAAGGAAAGTAAAGCAAGATATAGAACAAATTGAATGATGAAAACTGAGACAAACTGGAGACAGGGACCATGTCCAAAATGGACATCCATTATTCAACTGTAGCCTCATGTTATCATGTGGGAATGAAGGCCTAGTGTTGTAAGATTTTAGATTTTTCAAGAGGAGCCAGAAATTTGTACCTTCATACAAAAATTTCAATATTTGTAAAACACCATAAAAGTGAAAAAAATACCTACAAATCAGAATTTAGACTATGTTGACATTAACTTGCTACCTCTGTGATAAGCGATTTTAGAATAAGATTTTATTCTTGCTTAATTCTTCTCTTCAGAGATACCTAGTGTAGTAGGAGAATCTAACACTTAAAAACAGGTCAGGTATGGTGTCTCACACCTATAATCCCAGCACTTTTGGAGGACAAGGTGGGTGGATTGCTTGAGCCTAGGAGTTTGAGAGCAGCCTGGGCAACAGAATGAGACCCTGTCTCTACAAAAAATACAGAAATTAGTCAGCTGTAGTGGCCTACGCCTGTACTCCCAGCCATTTAGGATGCTGAGGTGGGAGAACACTTGAGCCCAGGAGGTCAAGTCTGCAGTGAGCCATGATCATGCTACTGCACTCCAGGCTGGGCAAGAGAGCAAGATCCTGTCTCATATAATAATAATAATAGTAATAATAATAACAATTTTGTGATGGGTGATAAATATCATAGGGGCAAAATGTCATGGGAGCACAGAGATGGGAGAGGGGACTTTCATAAGCCTAGAATGTTCTCAAGGAGTATGTCTCAGAGAATATGAATCTCAAATGAAAGTAGGCCTTTACCAGAGAGAGAGAAAATAAAGAGTATTCCAGAGAGTTGTGTGTGGGAAGATGCAGACATAAGAAACAGCAGCATTTACTTGGGGAAAAAATAGTTTAGGTTCTGTTCCCAGATAAGTGGAATTATATCAGATACAGTTTTTAAGGAGAGTCTATTTGGGGCAGGAGGGCCTCTTGAGTTCTTATTAATAGTTTTAAAATGTGAACACACCTTACTGCACATTAAGCACATGTACCCCAGAACTTAAAGTATAATAAAAAAAAATTTTAAAAAAAGAAAAAAAATGTGAACACACCTCTATTTCTCTCTCCAGGTAATTTTAACATGACCTTGCTACTCCCTTGGATGAAAGGATCATCACGAAGTTTTACAACAAACTTTATGGTTATGGAAGTTCT


Human Genome Map 8p11.2  (2100 bp sequence)
35A (no Pol III terminator detected; the transcribed region was arbitrarily considered to end at the beginning of a (TCCA)n simple repeat region)

TCTAAGGCTTCTGGACCTGAACTGAGCCATGCTACCAGTATTTCAGGATGTTCAGCTTGCAGATAGCCTGTCGCGGAACTTCTCAGCCTCTAGAATCACATGAGTCAATTCCCCTAATAAATCTCCTTTTATCTATCTGAACATCTCTCTTCATCTCTCCATCCATCCACTCATGTGTCCATCCATCCATCCATCTATTGCTATCTATCTATCCATCCATGCATCCATCCATTCAACCATCCATCCACCCATCCATCCATCCCTGTGCCATCTATATCTATCTATCTATATATCTATCTATCCATGCATCCATCCATCCATCTATCCATCTATCCATCCATCACTATCTATCCATGCATCCACCCATCCACCCATCCATCCATCCATCCATCCATCCATCACTATCTATCCATCCATGCATGCATGCATCCATCCATCCATCCATCCATCCATCCATCCATCCATTTATCGCTATCTATCTATCCATCCATGTATCCATCCATCCATCTGTTCATCTATCACTGTCTATATATCTATGTATCTATCTATCCATCCATCCATGCATCCATCCATGCATCCATGCATCCATCTATCACTATCCATCCATCCATCCATCCATCCATTCATCCATCTATCTGTCTTCTACCTACCTACCTATCTAACTCTCTGGAGAACTCTGACTAATAAACTAGCTTTATAAACATGTTATTCTCTCTCTGCAATGTCTATTGCTTTATCTTCAGGAACATTCCACACATCCTGTAAGACTTCAGTTAAATTATCTCTCTGTTTCTTCTCCAATCATCCTCTGCCTTCCCTAGTCTCCTAACGTACTTTGTACATCTGTCACAAACCCCTCATCATATTTACTGTAATTTTTTTCCTACAGATTTGGATAGGAATTGAGCCATTTTTTTAATTTCACTTTTATGGTTGTTACAAATAAAAGAGCAAGCAGGCCCCTCACTGTAATTCACCTGTATTTGCATTTAACTTATTAACCAAGGCATACTATTTCAAATAATCTAATATAGTATTTCCTATTTAATAACCAAACATACAGAACAGTTCCAAGCACATGTAACCATGTGATACATTTTCCTCTTTGAATAATAAATATATTTCTTATAATTAATATGTGATAAAATTGCAATATTTTTAATCTCCTACATCCTTCTCTTTTAATCAGGTTTCCTTATCAACTGGTTCCTATCTCACGGGGTTGTTGCAGAGATGAGGAAAAAAAGTATTCTATTGGTTCATGCATCTCAAAATAGGCAGATTCTTTTCTCTGCTTCTTCCTTCATTGGCTCAGGTGTGGAGTGCTTCTCCCAATTATATGTGCCAGCCTTGGTATGTTCTCATTGCTGTACCACACTGCCTGAGACATCCAAGACCACATCTTCCTTTGGGGGCACATTGGACCTTTGTCATTGGCACTGGCAGGGAAGCTTTTATTTCACCAGGTCTAAGGCAATTCTTCCAAAAAAATCCCAAATAGTGAAAGAATTGATTTATTCTTCTAATATTTAAGCAAATGTAAAAAAAAAGTTACATTAGTTATGTTTTTTTCAGATTTTGGATCAGTGAGACTTCATTAAAACACTTTGAGGTTATAAAGCAAGTAATTTTTGTTTCCAGAAAAGTTAGTTTCCTTTGGCTGAAGGGACATCTCTATGCAGGCCAGATCAAGACAAAAATAACTTTTAAGAAGGGAAATGAGGGAATGGAGTTTGGAAAACATAAATCCCACAGCAAAGTACGTCACCAACAATAAGAGTCATCTCTTTCACAGAGGCCTTTCCTAGAAAAGCCCTGACAGACTAGGAGTCCAATCTTCGGCTCCCATAGCACCCATGCCTGCTTCCACTCTGGAGCTTACTACTTTGCGTTGAAATTAATTTTTACATGTCTATGGCTTCTATTACAAATAGCTTATTGAAAAGAGAACCATGTACATTACAAATACTTTTTTAGAGTTGCTGAACTGAACAAATCAGTACCTACGGGGTTAGTATGCTGGCTTCTATTCCAGCAGGGTTTTGAGCCATGAGATTTTGAATGCTCCCGACATTGTTAGTTCAGGATGATTAAAAATAT


Human Genome Map 3p12  (2040 bp sequence)
36A 

TGAGAAAATGCAAGAAAGGAAGCCAGAGACGTTGTGGACTGCAGGCTCCTTCCCCATCATGTCACTCAAACACAATGTTTCATTTGTAAAACATATTTTAAAAGATTATGAATGCTATTTAAAAGAACACTAAAGGCCGGGCACAGTGACTCACTCTTGTAATTCCAGCACTTTGGGAGGCTGAGGTGGGCAGATTACTTGAGTTTAGGAGTTTGAGACCAGCCTGGCCAACATGGCAAGACCCTGTGTCTACTAAAAATACAAAAAACAAACAAACAAACAAACCCAACATGGTGATGTGTGCCTGTGGTCCCCACTACTTGAGAGGCTGAAGTGGGAAGATCACTTGAGCCTAGTAGGTGGAGGTTGCAGTGAGCCAAGATCACACCACTGCACTCCAGCCTGGGTGACAGAGCAAGAATACATATATATATATATATATATATATATATATATATATATATATATATGTATATATATATATATATATATATATATGTATATATATATATATACAGCAATCAACCAAACAACAGCAGCAAAACATTAAAGTGACATGCTGACTCCTTGGAAAGGTGGTAAGCTCTTTAGGCTTCCTAATCAGAGAAGGCAGATAAAAAGAGTAATTTAACATCTTTCTAATCTACCCCAATGGAATTTGCAGTGATTTTCTTTCCATTTTTTCTCATTGTTTTTCAACCTGATCACTAATAGGTAGCTGAAATGGAGTCACTTAATGGTTTTTGCTTTTTAAGAATTCCAAACTCACAGTTTGAAAATTATAGTTCTTGCATTTGAAGTTTTTCTTCATCGTGCTTGGCCTGCCTGGTGGCTTTTTGTTTTGTTTTGTTTTGGTTTGGTTTGGTTTGTGGTCTCTTTCATCTACCCAAAGCCAGTTGAAATAAATCAAAAGTTGTCTCACTCAGGAATTGTCTAAAGTAAATAAATGAAAAAAAAAAAAAAAACAAGACTAATAATTAGGCAACTCATTGAGTAGGCTGTTGAACCAGCTAAAGTGGGAAAGAAATTATTCAAGTTCTAAACCTTTCTACTTGCAAATTAGCCAAAATCAATTGCATTTTAAGCACTGCATCACCTTGATTGATTTTTAAAATGGATAGCACTTTGCTGTTCACATTTATGGTGAGCCGTGAAGGACTTGGCAATGGGCATCTTTTCTACCGTGTTCTGCATTAAACTCTTTAAATAGCTTCTGCTTCTTAATGTTGAATGAACTTTACTGCAGACTGAGTCTGAGGGTTTTTTTTTCAAGGTTGAAATACATTCTTCAGACTTTACTTTTTGCATGTAGCATTCTTCTTACTTAAAATACCTAAGAGTTTCTAACTAATTTCTTTCAGCCACAGGAAATATTTCTGTAATTTTAGGGTTAAAATGGAGAACTATAGGATGAAAATATAATACAAGAAAATGAATTAAACCCTAAAATTTATTATTATATGTGGGTAAAAGTAGGGGGGAAAATCACTGGTTTTGAAATTAAAAGATGAAAATTGTGAACACTTGCATGGAGACCCTATTTGTAAACTTGGACAAGCTTCAGTGCCCTCTCTCTGCATCTTCTGTCATTATTATTTTTCCTCAAGGCATCGTTTTGAGGATTAAATTAGAAAATGTGAACTAGCTTGAGAGTACAGTCAGCACTTGAACAGCATGGGTTGGAACTGCAAGAGTCCACTTATACACAAACTGTTTTCAAACAAGCTCTGATCTAAAATACAATATTTGTAGGATCTAAAACACGTGTATACAGAGGGCCAACTTTTCACATATGAAGGTTCTGCAGGGCCAGCTGCAGGACTTGAATGTGCCTGGATTTGGGTATAAACAGGTAGTCCTGGAACCGATACACCAAGTATACTGAGGGACAATTGTAATAGGCACAAAAATGTCGATTGATAGATTTTGTTCTTTTTCCTGAAATGCAAACCAGGACACTTACAAAACTAAATGAATAATTACTTATACATTTAGTGTCTCTGTGCTTCTCTCTTTTACCTTTTCCTACTTCTTCCGTAT


Human Genome Map 14q13  (2100 bp sequence)
37A 

AAGTACAATTGGCCAGGCGTGGTGGCTCATGCCTTTAATCCCAGTACTTTGGGAGGCGAAGGTGGGCGGATCACTTGAGGTCAGGAGTTCAAGACCAGCCTGGTCAACATGGTGAAACCCCGTCTCTACTAAACATACAAAAATTGGCTAGGCGTGGTGGTGGCACCTGTAATCCAAACTACTCGGGAGGCTGAGGCAGGAGAATGGCTTAAACTCAGGAGGCGGAAGTTGCAGTGAGCTGAGATCGCGCCGCTGCATTCTAGCCTGGGCAACAGAGCAAGACTCTGTCTCAACAAAAAAAAAAAAAAAAAAAAAAGTACAATCAGTGTTCTGTTGTGTTTTGTTGTTGGTTTTTTTTTTTTTTTTTTCTTTTTTTAGACAGGATCTTGCTCTGTTGCCCAGGCTACTATGCAGTGGCACAAGAACAGCTTACTGCAGCCTTGACCTCCTGGGCTCAAGTGATCCTCCCACCTCAGCATCCATAATAGCTGGGACTACAAGTGCACACCACCACACCCAGCCAATTTTTTAATTTTTTTGTAGAGACAGGGTCTTACTATGTTGCCCAGGCTGGTCTCGAACTCCTAGGCTCAAGTGATCCTCCCACCTCGGCTTCCCAAAGTACTGAGATTACATGCATGAGCCACCATGCCCAGCTCTATTGTGTTTCTTGTTTTTTAGCTTGACAGGAGGGTGTCAGGGATTTCCTGGCTTACAGAAATGATATGAATTTGCAGAAGAAACTAAAACTGTTAACAAACATTTGAAAAAAGTTAGCCCCATTATTTATTTTTTAAATGCAAATTAAAACAACATACCTCAGATTTAATGTAACTTCTATCAAAATCTCTGCTGGCTTCTTTGTAGAAATTGACAAACTGATTCTAAAATTCACATGGAAATTCAAGGGACCAAGAATAGCCAAAACAACTTAGAAAAGGAACAAAATTGGAGGGCCCACACTTTCTGACTTCAAAACTTGCTAGAAAGCTACACTAATTAAGACTGTGTGGTACTGGCATAAAGACAGACAAACAGATCAATGGAATAAAATTGAGAGTCCAGAAATAAACCTTCACATTTATGGTGAATTCATTTTTAAGAAGGGTACCAAGATAATTCAAGTAGGAGAAAAATAGTCTTTTCAACAAATAGTGCCAGGACAACTAGATATCCATATACCAAAAAGTGAAGTTGGACTCATACCAAGTACAAAAATCAACTCAAAGAGAAAAAAAACCTAAATGTAAAACTATAAAACTCCTAGAAAAAAAGATGTAAATCATTGTTACTTTGGATTAAACAATGGTTTCTTTTCTTTTTTTTTTTTTTTTTGAGACAGAGTCTTGCTCTGTCACCCAGGCCGGAGTGCAGTGACACAATCTTGGTTCACTTCAACCTTTGCCTCCCAGGTTCAAGTGATTCTCCTGCCTTAGCCTCCCAAATAGCTGGGATTACAGGTGCCTGCCACCACGCCAGGTTAATTTTTGTATTTTCAGTAGAGATGGGGTTTCACCATGTTGGCAAGGCTGGTCTAGAACTCCTGACCTCAGGTGATCTGCCCACCTTGGCCTCCCAAAGTGCTGGGATTACAGGCGTGAGCCACTGCGCCCACCCTAGACAATGGTTTCTTAAGGTACAAAACCAAAAAGACAAGTTATGAAAGAAAAAAAATAGATTGGACATCATCACAATTAAAAACTTTTGTGCTTCAAAAGTCATCTTCAAGAAAGTGAAGCCAAAAACAGAATGGGGGGAAAATTTTGCAAATATATATCTGAGAAGGACCTAATATCCAAAATGCATAAAGAACTCTTACAATTCAATAATAAAAGAAAATCAATCCAATTTTAAAATAGGCAAAGGATCTGAATGGACATTTCTCCATAGACTATACACAAATGGCTAATAAGCATATTAAAAGATGCTCAACATCATTAGCCATCAGAGAAACACGAGTCAAAAATCACTTACGATACCACTTCACACCTATTAGTACGACTATAATAAAAAAGACAGTTAACAACAAGTGCTAGCAAGGATATGGAGAAATTAGATCCTTTATATACTGCTGGTGAGAATGTAGAATGGTACAGCCCT


Human Genome Map 4p15.31 (2100 bp sequence)
38A 

TAGAAATATTGCAATGGAAACTTCAGAAGTAAAAATGATTATAACGGGCTATTATAAACAATTATGTGCAATATTTAATCAGGAAGAAATAGAAAGCTTGAATGGACCAATAAAAATTAAGAGATTGAAATATGAATTCAAAACTTTGCAACAAAGAAAAGCCCAGGACGAGATGGCTTCATGAATGAATTCTACTAAACATTCAAAGAAGTATTACCAATATTTAAATTCTCCCAACAAATAGAGATAGAAGAAATACCTGCAAACACATTTTACAAGGCAAGCATCACCTTGATCCCTAAGCCAATGACATCACAAAAAAGAAAACTATAGGCCAATATCTCTGATGAACATTGATGGAAAAATTCTCAATAAAATATTAGCAAACAAAATTCAACATCACATCAAAAAGATTATACATCATGACCAATAGGATTTATCCCTAGCATGCAAGGCTGGTTTAACATACACGAATGAAACAATGTGACACATCACATTAACAGGATGAAAGATAAAAAACACAGAATTTTCTCAATCAACACAGAAAAAGCATTTGACAAAGTTCAGCATCCTTTCCTGATAAAAACTCTTAACAGTTTATGTATAGAAAGAAAATTTCTCAACATAATATAATAAAGGTGATTTATGAAAAATCCACAGCTAACATAATAATCAGTGGGAAACAGTTGAAAGCTTTTTCACTAAGATCCAGTGCAAAGCACAAATGCCCACTTTTGCTACTTCTATTCCACATAATATTGGAAGTACTAGCAATAGCAATCAGACCAGAGAAAGAAATAAAAAGCATTTAAGTCAGAAAGAAGAAAAAGTAAAATTATCTCTATTTGCAGATGATATAATCCCTTATGTAGAAAACCCTAAAGATTCCACAAAAAACTGACAGAATGAATTAATTCAGTAAACTTGCAGGATACAAAATCAACATACAAAAATCAGTAGCATTTTTATACACTAATAACAACATATCTGAAAAAGACGCTTTAAAATCCCATTTATGAAAGCATAAAAATAGTTAGAAATAAATTTAACCATAAAGGTGAAATATTTGTATACCGATAACTATAAACCTTTGATAAAAAAAGTTGAAGAAGACACATATAAATAGAATAATATTCTGTGTTCATGAATCAAAAAATTTAACAATGTTAAAATGTCTGTATTAACCAAAGCAATATACAAATTCAATGCAATTTCTATCAAAATTTCAAGGATATGCATCACAGAAATAGAAAAAAAATTCTTGAAATTCATATGGAACCACAGACACATAAAAACAGAATAGGCAAAGGAACAATGAGAAAGCAAAACAAAGCTTGAGGCATCACACTTCCTAAGTTAAAATTATATTGCAAAGCTACAGTAATCAAAAACAGTATACAAATGGCATGAAAACGAAAATGTGGACCAACGGAACAGAATATAGAGAGCCAGAAACTTAACTAATTTTCAACAAGGGTACCAACAGGACACCCTGAAGTAAAGATAGTTTCTTCAATAAATGATTCTGGGAAAATTGGATTGCAACATGCAGAAGAATGAAATTGGACCCTAATCTTGCACCATATACAAAAATGGACTCAAAATAGATAGGAGACCTAAATGTAAGATGTGAAACCATAAAACTCCTAGAGAAGAACATAGGGGGAAAAATTCCTTGATATTGGCCTTGGAGATGATTTTTGGATATCACACCAAAAGCTTAGGCTACAGAATCGAAAATAAATAAATGGAACTACATCAAACTACAAAGTGTCTGCACAGTAAAGGAATCAATCAACCAAATAAAAAGGCAACATACAGACTGGGAAATATATTTTCACACAGCATATCTCCTAAGAGGCTAATATTCAACATTTGTAAAGAACACTTACAAATGAGTAACAGAAACAACAAACAGCTTGATTAAAAACAGGCAAGGGACCTGAACATACTTTTCTCCAAAGGAGAAATAATGGCTAACAGGATATGAAAAGGTATACAACATTGCTAATCATTAGGGAAACACAAATGAAAACCACTATGAGATATCACCCTTCACCCATTAGGATGGCTATTATAAAAAAAAAAAAAGACAAGAGG


Human Genome Map Xp11.3 (2100 bp sequence)
39A 

AGTAAGTGGTGGAGCCAAGAATTGAACCACAATGTTCAGGATTCATAGAATGATTAGAATATAGTGAAAACAAAGCAAGAGATAAATTAGAGAGCTGGCAGGCGGGGCCCACTCATGAGAGATGCATGTGTACGCCATGTAGGATACCCAGGTTTTCTTCTTTTGGTGAACGGAATCTCTTGAAGGGTGTTGAGCAAGACAGCAATATGAGCAGAGAGTTAGAAGCCCCACCACTCAGGAAGCAATGCAGAGGTAGGCACGGGAGTGCATCTGAGATGAAGAGAATGGGCTGAGGTTGCAGCTCAGAGGCATAAACTAGAATTGAATGTGCTGGGCCTCGGATGGACTCTAAGCCTCTTGTTGAGTGTTAGGGAGACAGGGCCATCATGGAAGCCAGCTTTGGACAGCAGGAGGGACACCTTTTCTACCGAGATGAAAAAGGAATGGCAAGTTGTGGATGTGGGGGCATGGGTGTGGTTTACATCTCAGGATCTCTATATGCTCTATTAACCTGGAGATGAGGTTTTCTGTTATAAATTGGAGGAAAATGGTGAAGTTAGGAGCTTAAGAGTGAGAAAGTCTGAAATATCCATTGTGGAGAGTGGAAACAGGTACAACTAGGCCTTGATTCCCTCCTTAACTGCTTCTTCATCCTCAGCTACTCACTGATCCATCAGTTGTCCAGTTTGTCATCTGTGACTTTGATTTCTATTGGCCCTTTCCTTTTGGACCTTATTGGTCCTTTCCTTTCAGCATATAGAATGTTCATGTCTCTCTCAACTTAAAACAACAGCAAAACCCAGCCCATGACCTTCTGCCCGGTAAAGTCATCCTGGTAGTGTCCACCAGCCTGGCAGAACCTGTCTTCCACAGCAGCCTGGTGACAACAAGAAGCCACAGCAGCAGTGTTTGGCAGTGGTCTGGCCCTCTCCAGACAAAATAAGCATCCCTGGGGTTCCTGGACAATGAAGGGCAAAGGAAAAGAGAACAGGTCTCAAGAATATACTGACAAATCTCCAAGAGGAATTCAAGATAATATGGCAAATGGGAATTAAATGAGAAAGTAAAAGAGAGGTGACCATATGCCAATCTGGAGCAGGTGGAATGTGTCATGATGGTTCAAGGGCAATCAAGAGTTCACATTTATGGTGAGGATTTCTCTTGATTTTTTTTCCTCCTGTCATCTTATTTTGTGTTTTCAGTTTTTCATGTATTGTTACCATTACCTTATATTGATTGCTTCTTGTCTTTTGCAATACATCCTAAATATAGCAGGTTCTTGAATAACATCACCTCGTTCAACATCATTTTATGATAATGTTGATGGGGAAAAAAAAATGGCTCCAGGCTGGAGCCACTGTCTGCATGAAGTTTGTACTTTCTCTCCATGTCTGCGTGGGTTTTCTCTGGTTTCTTCTCACATCCCAAAGCTGTGCACATGAGGTGAGCTGGCATGTCTATATGGTCCCAGTGTGACTGAGTGTGGATGTGTGAGTGCACCCTGCGATGGGATGGTGTCCTGTCCAGGGCTGGTTCCCACCTTGTACCCTGAGCTGCTGGGACAGGCTCCAGCCACCCATGACCTTGAACTGGAATAAGCACATTGGAAAATGAATGAACAAATGAATACAAATTAGGATAAAATAAAAACTCATCAAGTCTATGACAATAAAGGACATGGGACAAAAGCGCTCAGCAAGCCTGCTCTACTTGTGATTTTTTGGTTTTGAACTGCAAGGTGGGAAAAGATGCTCCTTACAATGTTCGCTCTGCAAACATTTATTCCCTGATTTAACCCATCACTACCATGGCCACTGCCACTCACTGATTCACCAAAATTGGGTAAATCATTGTCTTGTTTTTATTAATTTTTTTTTTTGAGACAGAGTTTTACTCTTGTTGCCCAGGCTGGAGTGCAATGGTGTGATCTCAGCTCACTGTAACCTCCGCCTCCCGGGTTTAAGCGATTCTCCTGCCTCAGTCTCCCGAGTAGCTGGAATTACAGGCGCCCGCCACCACACCCAGCTAATTTTTTGTATTTTTCGTAGAGATGGGGTTTCACCATGTTGGCCAGGCTGGTCTCGAACTCCTGACCTTAGG


Human Genome Map 11p15  (2100 bp sequence)
40A 

AAAGGCTTCCTTCCCTCAACAAAAAGGATCTCACCATTCTTTATATTCCAGGTTTACTTTCTGATTTACCCGTACAGTATCATAGCCTGGAGGCTCCTGAGATGCATTCTTTTTTGGAAGGGGCCTGATTTTGAAGTCTGACTCAGCCACTGATTAGTGAGCAGGCTTTCCTAAATCTTCCTTTCCTCTCTAGTAAAGTAGGTCAGATCGTAATCTCTTCCTCACAGGACCATTGTGCAAATTAAAGGAGATAACGTGAATGGTTTGGTAGCAACTCAATGAATGTATCTGAATCCTCCCGTCAATTTACTCTGCTGTCCATTATAGTCAGTTATGTATCTGTTTTATCCTGTCTGGTCGAGAATGGAATCTGAGGACCAAAACTCCTCTCTTACTCATCTCTGTGCATTTTCCTCTTTGCCCCTGCCCCTTCCTCCAACCCAGTACCTAGCATGGTTTCTTCATTTATGTGTAAATTTTATCAAAGTGGTGTCAGTCTGTGCAAATCATCTGCTGAAGTCTGTTAAGAACGTTCAGCAATATACACTTCAACAGTAACAGGGACAGTGTGGAAAACCCTTGTTTTCTTCCTACTCTCCAATTCTTCTGCCTCTTTCCACTGCTTTGGAGAGGAGGTTACAATCAGAGTCATTTCACCATAAGAAAATCATCAATTTTCTAATGAAAACCCCCCTCCTTAAATATTCAGCCGTGGCACAATCCCTAAAGGAGAGAACACATTAATGCAATGCACTTGGCCAATTTGTGAGCCCTGACTTGTTGAGCACAGACTCTTCCCTGACCCTGGAAGACAATCGGCAGGACCCCTGAATAATGAAGTATGAATGCTCACCATTGTGCTATCTCTAGCTACACCAGCTTCTACCTAATCTTTTTTTTCCTTTTCATTGCTCTCCTTTGCAACTTCCTAAGGATTTGGCCATTTTCCACTGTGTCCAACCTACGAAGCTGTAAATACCTGACTGAGAAACACAAAATGTGTTTATAGGACTTCTGATGGCTTCTCATCTAAACCATAAATGTGAAATGCATTTCAACATTTCTCAGAAAACACCCCATCCCCCAGATTCAATTGGCAGCACAAATTTACTTCTCAGAAGACAGCACCAAAATCTCAACATTGGCATTTTGAATCAAGCACACACACGCAGCTGCATTGTGTTAACAAAAGTGAAACATTATTAGGTCGCATACTCTGAGTGACAATATCCTCGAATGATCATTTCTGTGAGAAATTAAGCTATGAGAAATTCAATTTGGTAGCATTTGGGCATTACAGACAGCTAGTCTGTTTATATCAGCAAGGCTTTGATGTTAAGACTCTGTAACTGCAGCCACAGGAAAAGCAGACTGAATACAGGGTGGATAAGGTCACAGATATAAAAATCAGATAGAGTTCTGTTCTATTATCTACATAGTGTGTACTTTGGGAAGTTACTTAATATTTCTAAGCCTCAGTTTCCTCATAAAAATAAAAATGGCAAGCAATATGAAAACTATCTAATAGAATATTTGTGACACTAAATTGTAATAATGTATATAAATCACTTAGCCTAGTATGTGGCATTTATTAACACTCAAGCAAAAGTGTAATTTTTTAAAAAAAGACTCTTATATCCCTTACATGACAGAAATATTAAGACCAAAATGGTTACTGAGCCCTCAAAGGTATTATCTCATTCTGGCTGAGCTATCTGGACCTGGAGAAAAGTCTAGAAAGACTCTATTTCACTCCAAGTTTCTTGACCCTATCTTTATTTTTTATCTTCTATCCACTAGGACCTGTGATCAGGCCAGGATTAACCAGTGTTCTCTAGGATTAACGTTTTTGGCAGCTGGGGATGATTGCCTAAGATAATTGTTTTTGTGTCTGCCTCTCCTGCTAGAATGCAAACTCTCAAGGGCAGGACATATGTCTTTCTTTATCCTACCTGTTAGTGATCAACAGGAGAAGGCCACTGCTTAACTGTTAGTGTCAGGTCAGCTCCAAGCTGGTACTTCTTAGGAACTCTTTTCTTTTCTTTTTCTTTTTCCTTCCTTCCTTCCATTTCTTTCTCTTTCTTCCTTCCTTCCTTCTGT


Human Genome Map 2q31  (2100 bp sequence)
41A 

CACGTGAGGACACAGCATAATAGTAGCCACCTGCAAGCAAAGGAGAGAGGCCCAGGAGAAACAAGCCCTGCCAGCACCTTACTTAATCTTAGACTTCCAGCCTCCAGAATTGTGAGAAAATAAATTTCTGTTGTTTAAGCTATCCAGCCTGTGATATTTTGTTATGGCAGCCCCAATAAACTAGTATGTGTATAATGAAGCCCTAGACAACAAGGGACTCTCATTTCTCCGCATATTTGTAGAACTCATCCCAATTATATAGAGCTCCTACTCTGAGTGCTAGACACAGTGTTAAACACTTTCCCTGTGTTATCTCGTTTAACCATTAAGCTGAATCCTCCAAAACCCTTTGGAAATCAGACTTATCTAAGAAACTCACTATTGTAGTGAAGCTGTTTTAAAGAAGAATTGAAGGTATTTTTCTTTATCTTATAATCTGTTACATTGTGTTACATTTTAAGATAATACTAATCTAAGGACTGATAACAATTTAATTTGCCAGAATCATTAAACCAAATAACATCTTTAACAGTGGCTGCTAGACAGGGGCAGCTGTATATTTTAATGCCATATTTGGGGGAAAAAAAACAGGGTAGCAAACATATCTATAAATAAGAATTAATTGCTACAAATTACCTGGGAAGGGAAAAATGTCAAGTTCATATAAAGAATATTATTGACCCATGGATTTACAGCTATATAATAATTTGGTACCTGGTTTATTTCTTAAAGACCTAGCACGTTTCTTGTTTTCTCCTGCTATATTACGTGTACATGGCGTTTCAATAATCAAGCAAAAAAGATGTATGCACTATCTTAGTCTTTGTTGTCTAATTAAAACTTTTTATGCATAGCAATTGCTTACCATTTTGCATTATCACCAGAGCTCATTTCTCATGGAAAAAAAATTAGCATCAGTTTAAAAGAATATTTCTTTAATCAACAGTTCTGATTGTCAGTAGTACCATTTTGTAGATAGTTTTTAGCTGACTAACAAATCTTTTATTTTATTGGCTGTCTCATTTTGCTCTCTTGCATATTTCACATTTATGGTCTATTCAGACATTCTCCTGTTTTGTTAAGTGGAAATCTGTGTGGTCTTTGATGTAAGACATAATTTATTTGACAAGGAAATATGAGTCTGTGCCCTGAATCCACATTTAACTGATGGATTGAGAAATTTTAAAATTGCAACAAGATAGACTCTCCTCCAGATTGCCGTACTACTTGCATTTTGCTTATCTATTTGGGAGTGAATTTACATATGTGTGTCTATATACGAATATATAGAGAGTCATACAACCATGCAGCTGTACTTGTGCAATTTTTCTACTTTGTTAATAGAAAATGCAGTCTCATTTTGTTAGTCATTAATGGTTCCTATAGAAAATTTTTAAAGAATTTTTTTCTGAAATTAAATTCAAGATACTTATTATGTTTTATCTTCATATAGATAGCTTTATAAAGAGAGTGATGTCTTCAAGTCTGTACTGCTCGCTTCTCAGCCTAGTAAATGGAAGTTTTGTTAGCATTTCAAGATTTATATATTTCATATGTTCTCCCAAGTCTATGGCCCAGTTCTGGTAATTGGAAACTTACTTTCAGCTCATTCCCTCTGCTCAGACTACTTGTCAATTAACCTTTGCAAAATGATAGTTTTAAAAAATATGACTTTCATATTTCAATCATGTTCATTTTCAATCATCTCAAAATGTAGAAATTGAATAACACCCGGGGTTCTACAGTGCTTTTTACATATCATTTAAGGTTTAAAACATCTCTTTGATGTTCAAATATGACTGCCATTTATATTCAATGGATGAGATTAAGTGGTTAAAATTACTTGTACTGGGCATGCCCCTGCTTTGTTTATAGGTATGAACAAAACACTAAGGATTTTTCATAAATATGCACCATTTCCATTGATGTTTTTGACTGCTGTCTGTGACACACTAGGTAGGCCATATTAAGTAATGGGGAAGAAATCATAGGTCCTACTGTGATATTAAAAATTTACATTTTGATGAATTAAATAGAGTTGTTGACCATTCTACACTGTTGATTATATGAAGGGAAAAAGCTAACAACTTCTAAGAATAA


Human Genome Map 3p12.3  (2040 bp sequence)
42A 

AAAGGTAAAAGAGAGAAGCACAGAGACACTAAATGTATAAGTAATTATTCATTTAGTTTTGTAAGTGTCCTGGTTTGCATTTCAGGAAAAAGAACAAAATCTATCAATCGACATTTTTGTGCCTATTACAATTGTCCCTCAGTATACTTGGTGTATCGGTTCCAGGACTACCTGTTTATACCCAAATCCAGGCACATTCAAGTCCTGCAGCTGGCCCTGCAGAACCTTCATATGTGAAAAGTTGGCCCTCTGTATACACGTGTTTTAGATCCTACAAATATTGTATTTTAGATCAGAGCTTGTTTGAAAACAGTTTGTGTATAAGTGGACTCTTGCAGTTCCAACCCATGCTGTTCAAGTGCTGACTGTACTCTCAAGCTAGTTCACATTTTCTAATTTAATCCTCAAAACGATGCCTTGAGGAAAAATAATAATGACAGAAGATGCAGAGAGAGGGCACTGAAGCTTGTCCAAGTTTACAAATAGGGTCTCCATGCAAGTGTTCACAATTTTCATCTTTTAATTTCAAAACCAGTGATTTTCCCCCCTACTTTTACCCACATATAATAATAAATTTTAGGGTTTAATTCATTTTCTTGTATTATATTTTCATCCTATAGTTCTCCATTTTAACCCTAAAATTACAGAAATATTTCCTGTGGCTGAAAGAAATTAGTTAGAAACTCTTAGGTATTTTAAGTAAGAAGAATGCTACATGCAAAAAGTAAAGTCTGAAGAATGTATTTCAACCTTGAAAAAAAAACCCTCAGACTCAGTCTGCAGTAAAGTTCATTCAACATTAAGAAGCAGAAGCTATTTAAAGAGTTTAATGCAGAACACGGTAGAAAAGATGCCCATTGCCAAGTCCTTCACGGCTCACCATAAATGTGAACAGCAAAGTGCTATCCATTTTAAAAATCAATCAAGGTGATGCAGTGCTTAAAATGCAATTGATTTTGGCTAATTTGCAAGTAGAAAGGTTTAGAACTTGAATAATTTCTTTCCCACTTTAGCTGGTTCAACAGCCTACTCAATGAGTTGCCTAATTATTAGTCTTGTTTTTTTTTTTTTTTTCATTTATTTACTTTAGACAATTCCTGAGTGAGACAACTTTTGATTTATTTCAACTGGCTTTGGGTAGATGAAAGAGACCACAAACCAAACCAAACCAAAACAAAACAAAACAAAAAGCCACCAGGCAGGCCAAGCACGATGAAGAAAAACTTCAAATGCAAGAACTATAATTTTCAAACTGTGAGTTTGGAATTCTTAAAAAGCAAAAACCATTAAGTGACTCCATTTCAGCTACCTATTAGTGATCAGGTTGAAAAACAATGAGAAAAAATGGAAAGAAAATCACTGCAAATTCCATTGGGGTAGATTAGAAAGATGTTAAATTACTCTTTTTATCTGCCTTCTCTGATTAGGAAGCCTAAAGAGCTTACCACCTTTCCAAGGAGTCAGCATGTCACTTTAATGTTTTGCTGCTGTTGTTTGGTTGATTGCTGTATATATATATATATACATATATATATATATATATATATATATATATATATACATATATATATATATATATATATATATATATATATATATATATATATATATATATGTATTCTTGCTCTGTCACCCAGGCTGGAGTGCAGTGGTGTGATCTTGGCTCACTGCAACCTCCACCTACTAGGCTCAAGTGATCTTCCCACTTCAGCCTCTCAAGTAGTGGGGACCACAGGCACACATCACCATGTTGGGTTTGTTTGTTTGTTTGTTTTTTGTATTTTTAGTAGACACAGGGTCTTGCCATGTTGGCCAGGCTGGTCTCAAACTCCTAAACTCAAGTAATCTGCCCACCTCAGCCTCCCAAAGTGCTGGAATTACAAGAGTGAGTCACTGTGCCCGGCCTTTAGTGTTCTTTTAAATAGCATTCATAATCTTTTAAAATATGTTTTACAAATGAAACATTGTGTTTGAGTGACATGATGGGGAAGGAGCCTGCAGTCCACAACGTCTCTGGCTTCCTTTCTTGCATTTTCTCAG


Human Genome Map 4q13.3 (2040 bp sequence)
43A 

CTCAGTTTTAAATGTTTCCATCAATCAGTAATTCAGCTCCAGAGTTGCCATAGAAGGTTATGGGAAAAAAATCCTTCTGCTTTTCCAATATCAAAGAGAGAGATGTTCTGGAAGGTTTTATTTTTGCCACCCTGTTTCTAATGCATTTCGCCTTAAGAATAATATTACTCATCTCCAGCAATCAGGCTCAAGGAGGAAATTTGATACCATTCTGTGGGTCATCCCCAGATCTCTGCAGGCTTCTGGCAGATGTATGATGTAGTGGGCACCACTAACTTTGTCTGCTAGGGTATTAGTAGGACGTGGTCTACTGAATACCGGACAGACATTTGGAAATTAAACTAATCAAAATAGGCTAATTTCACGTGCTAAGCAATGCAATTTCCCTGAATTTGTAGTTCCATGATCTACTTTTTCTTCTACATTTCTCTTACTCCCTCTCTTCTCCGTTTACAACACAAAATTCAACAAACTGCTAACTCTAGCTATTAATATCCCATAGTATTTCTCTAAGCAGCTTCATAGTCACAGTTTCAGTTAAGGCTCTAGGGCTTTCATTCCTGAACTACGTGTACTACAGATCCCAGTTAAAATCTCTATCCTTCCCACAACAGTGGTTGACCACCTTCCTCAACATTGGATTTGGGGTGATAATGACAACATTCTGAACAAAGCTCATTTTTTCCCCTAACCCCCAGCTAAATAGAAGAAATGTTAATGTTACTCCTCTTTAGATTTTTTTAATTAAGAATATTTTTAAAGGATTTTTTTATGATTTAATGGGACACAATGAAAAGATATTTTGACAAAGGTAAACATCTGAAACTGAGGCAAAGAATTTTAGATGTTGCCTGTGTAACCATTATTCCATGATCAAAAGCCCAATGTTTAACATATCCTGATTTCATCATTAAACAAGCATAAGAAAAAAAAAAAAGAAAACCATAAAAGTGAAATAGATTACATCTTGTAATAATACAGCTATGAAATTCTGACCAGAATGAAAATATGAGTATGAAGAGAGTAATCATTTGCTTATTAATTCAAGGAACAATTTGCCATTTTTCAAGTATTATGAAAATAAGAGACTGTTGGACTCTTTTTAAACACGCAGGTTTTTCAAATGTATGTACAGTAATATTATAGCTCTGGTGAAAATTTTGATGAAAACAAAATTTTCTGTCTTCTTTTACTTACCTTGCCCTTTTCAAAAAATAGTGCTTAATGTTAACCAATGGACAGTCTAACTACCTGAAGCTTTTCATTCAGCTTTATTTTCTCAGCAACTATGGTTGAAACTGACAAGTTAGGTGAAAGGTTGTGTAAGTATCCAGGCAGGGGCAAAAATATCGAGTTATCCCCAAATACTAACAAGCACATAGGTAGAATATTTCTACCAAGTTAAAGAGAATAAAGGAACCACATTGAGCAGAGCTACCTTATTCAAGGACCTCATTATCTCAAGGCACCCCAATTGAATAAGTGTACCATTATTCCCTTCGTTCTGTGCAAACAGCAGACGTAGAGCACAAAGAGAGACGATTTCAGTGAATCACACTGTAATTATAAATGCCACATTAAAAAACGGAACAAAAGCAACAGCAGGACACTGTAACGTCGATGGTTAAGGAGGGCAAACAGAGAAACATTCTCAAAGGCCACAATAAATTACATGATCCAGTTCTTTGTTACAGGCAAATTATTGGACAATAAGAGAGACACTGAACACAACTTATCAGTGGTAAAGTAACTTGCACACATTCTCCTACCACTGAAATATTCCTGTTACCTACACAACTGCATTTGTATATACAAGCAAGAATTTTGATGCACTAAGTAATTAATATAGCTCTCTAGTATTTTTTTTACCTCTGTGCTTATTCTATCTGGGCAAGGTGTGGTAATAGCACCTTAAAAAATAAGATCAGATTTAGGAGTGAAGAACACTGCATTGGAAAAGGTAATTGCTAATTTTATTGTATTTTAATTATTTGACCATTTGTGCACAAAATTAAATAATACTTGCTTCATCTCTATATT


Human Genome Map 4q13.3  (2160 bp sequence)
44A 

GTGTATTGCTGTGAAAAAAGTCACTTCAAATTTAGTAGCTTCAACCAACAATCATTTTATTTGCTACCAATTTTTTGGGTGAGAAGTTTGTGCTGGGCTCAGTTGGACAGTTCTACTGATTTCCCTGTATATACCTCATGCAGCAAAAGTCATCCTGGTGGTTCTATGTGATGGTTAATTTTTGTATCGACTTGGCTGGATGATAGTCCCCAGATATTTGGTCAAACGTCATTCTAAATATTTCTGTCTATGTGTTTTGGGAATGAGATAATATTTTACTGGATTATTCTTCACAATGCGGGTGGGCCTCATCCAGTTAGTTGAAGGCCTTAATAGACAATAATTGACATCAGACCTAAGTAAGAAGGAATTCTGCCAGCAGACTGCTTTTGGACTCAAATTGCAACTCTTCCCTGAGTCTCAAGCCTGCTGGCCTACCTTGCATATTTTGGACTTACCAAGCTTCCACGACTGTATGAAGCATTTCCTTAAAATAAATGTACATATCTGTCTCTCTACACACATACATATATACACACTCTCACACACATCCTGTTGGTTCTCTTTCTCTGGAGAACCCTAACACACTCCACTCAGACAGATTGTCTAAGGTGGCTTCACTTAAGTTTTGGATATCGACTCTTGCCGTCACTCTCTTGAAACAATGTTCTTAGACTCTCATGTAAAGAAACTCCATCTAGCTGCACCCTGGCACTGTCCCAAAAAAGTGAGGATAATACCTGCAAGGTTTCTTAAGGATTATGCCTTGAAGTCATATTTTACTTCTGTAGAGTTCTATTAGATCAAACAAGTTACCTGGCCAAGCCTAGGGTCATAGTGGGAGAATTTTGAGGGCCATATGGCAAACAGCCCACCATACAATACATTCTCAAATGGCTTCTCAAATTTTACATTCTTGTGAATGATTCTCTCTTCTTGTGATTAATTTTACACACATTACTTCAATATAAATTTTTATCTCATTGCATTTTCAGTTTTTTTGTAATTTCACATTTATGGTACTCTTACAATGTGCCATGTGCTATTCTAAGTATTTTATGTAGATTAATTTAATTCTTACAACAACCTTTTGAGGTAGGTAATACTATTTTGTTCCCATTTTCAGATGAAAATGCCAAGGCATAGAAAGCTTATGTAACTTGCCCAGTAACACTCAGAGACTTAATGTCACAACCAGTACTTAAATGTATACTATCTGACTACAGGGTATGCATGCTTAGTCATAATGTTATTAAAATATCATTTGTGATGACTGAGGCATCATGGCAGATAGGAGGCAGGACTAGATTGCAGTTCCAGACAGAGCAGGAGACAGAGGCTTGCACATTGAATTTTAGCTCCAGATCGACTGCAAGAGCAAACCGGTAATCCTGAGAGGACCCACAGATCCTCTGCCGGAAGCAGACTGTTTCTGCAGGACCAAGGAGACACCACAGATACTGTGGGTGTCCCAACTGCAGAAATTGGAAAGGGAGACCCTTCTCTTCCAAACACACACCCCACTGGAGAAGCTGTTTCTGACTTTACCTGGAGCTGAGTCAAGTTAGAGAGCTGAGCCAAGTGAAATACAGGGGTAGGGGAAGTAGCGGAAAGACCCTGGGAGCTCGCTGGGTCCCCCAAGCAGCCCATACCTGCCTGGCACCACAGGGATCCACTGGGAGGTTGGCCAGAGAAGTAGGGGGTAAAATACCACAGGCAGAAGGAATTCTCTAGCTAAACTCTGTAACAATTTGAACGGGGCATGAAGCCTCCTGGCCAGTACTTCAAGGAGGGTGTGAATCCAGCATGCAGACCTCACAGGCAGGGGGGAAAACTAAAGCCCTTTTCTTTGGCAGCCGGGAGGTGGAAAGCCTCAGGCAAGTTTTCAAGCAGGGCTCACCCTCCACCTGGAAACAGACTCCAGGTTGTTGAGGGGGACACGGTGGGAGTGAGACTGGCCCTTCAGCTAGCATGTGAACTAGGTGAGGCCTGTGACTGCTGGCTTTCCCCTACTTACCTGACAACCTACATGACTCAGCAGAGGCAGCCGTACTTCTCCTAGTGTGTCCGGAATTGGTGGGTTCTTGGTCTCGCTGTCTTCAAGGATGAAGCCGCGGACCCTCACGGTGAGTGTTACAGTTCTTAAAGATGGTGTGTCCG


Human Genome Map 4p14 (2100 bp sequence)
45A 

CCTCCCACAAGGTCCAACTCAATCAGAACCAAAAGGGAGATCACAGCATATCCATGCAATCCCTGGCTGGACAGACGGGGCACCCTAGGGCCTGGAGTTACGCAGCTGACTGGCAGAGGTCAGTACCCAGTTCTACCCAGTGTGGCCACCCGATCCAACTCTGTGACTCATTTTAAATCATAAGTAGTTTGAACAAAGACTTAAAATTAACGGGTTTGGTCATCATTAAAGTTTATTTTTAAACGTCAAGTAATTTGGCATTTTACTAACAACAACTTGAGGATATCCACAGTGTATGAAACACCAACTCTTGTTTCAATAAAAGTCTAATGAAAGTTTCTCAAATTCTGAAACCTAATTCCAGGATTCTTATTTGGAACTGTTTTTTTGTTTTTGTTTTTGTTTTTGTTTTATCAGAGTACATTAACTCAGGGGGAAAATGAGATTATCTTTTGATTCAGAGAGAAACAGAACATTCCACTGATAGTTTAAAAATAACACAGTGACCACAGATAACTGTAGTTCAGCAATTTTCAAATTCAGATTCTGGGTCAGGGCACAAGATTATGCGTCTTTAACAAGCACAACTGATTATAATGCTGATGGTCTAGAGGAAACTTGATGGGAAACACTGATCTATTCAACACTTTTTAAAAGTACACTTTAGTACTATTTCTACCTAGTCAATAAAATAAAGAAGGAAGGAGAAAGGAGGACGAATAAGAGAGAATCCGAAAGACACACTACCCAGACAGACTACTCAGACAGACGAGACAGTCAGGCAGACATGGTGCTACCCGTCAAGCATGCAGGAAAGCAGCTTTCATGGAATAACATTCCTAAACTCTTGCCTAATAAATTATGCTGAGAGCTGCTGCTAAGAATTTTTTAAACCAACTCAAAGCAAAAAAGGAGCTATTCACCCAATCAACAGGTGAAATCAAGTCACAGACTAGTATAGGGTTTGGCAGATTTCAGGCCCTCCAGAAATATCTGTTTAATTGAGAAGCAACTCCAGCTCTAGCTAGAAATCTATTTAACCATAAAAGTGAAATCATAATGAATTTGGTCGTATCTTATTTTTCCCCTTTGTTTGTTTCCTCGGGGCATCTATAATGGCTGAATTGGAAATGGAACCACAAGTATTATAACAACATTTGTTGGAAAGTTCATCCTGTATTTTAGTAGTACATAAGTTGACAGATATGGCTTTATGAATTGTTCTCAGAGACTTAAAAAAAAAAAAACCCTGAACTTTGTAAAAATTACATCCATTATCCACCAAGTAACATTTGCAAGCAAAACCCTCTACTAGAAAAAATGGGTGCGAAAATAGGAAAAGGAGAAGAACAGGAGGAAGAAAAGGAGAACAGGACGTACAATTAATTGAGGGGAAAAAAATCATGAGTAAAGAAGTCAGAAATAAATGTAGCTAAAAATACAAACTGCTACTTTATGGTCCAGATATTGTAATATATCATTTTTAACATAAAAGAAAAACAAATCCTCAACAGACTTCCTATAAACGAAATTATCAGAGTTCCCGAGTACACCGGGGGTCGAGGGAAGAATCTCCATGTGCTCCGAGTATCGATAGCCAGTCCAGCTTCATTCACTCATTCATTTCTTTTCTTTCATTTCAGGAGAACATTTAGCAGTGTTTTGTTTTATTTATTTTATTCAAAGGGAAATCCTCATGTGACACTAGCGGTGAAAATAACTTGTATTTGTAAGTTAATGTCTGCTGTACATCTGAGTACACAATTGTCTTTCACAGAAGATGGAGCAAAGTATTACGGAAAGTTCATTGGCTTCTGAGTCTGAGAGAAATGGGTTCAAATCCTGAATACGTTCCTTATCTGTGTGATCTTAAGACTCATCATTTAATATTCTGAGTCAGTTTCCTCCTCTATAAAACAAGAATCAGACGGGGCACAGTGGCTCACGCCTGTAATCTCAGCACTTTGTGAGGCCAAGACGGATGAATCATCTGAGGTTAGGAGTTCGAGACCAGCCGGACTAACATGGGAAAACCCCCGTCTCTACTAAAAATACAAAATTAGCCGGGAATGGTGGTGTACGCCTGCAATCCCAGCTACT


Human Genome Map 2q22.1  (2040 bp sequence)
47A 

TTGATTCATGGGATGTTTATGTGGATAATTCCTTTGAAATCCAGCTTGATTTATGAACAATCTTCTCTGCTCTATTGAGCCATTAAATCCAGAGTATTAGTGCATTTGGAATACACAGAGATGATAATGACATCCAAAGAAGAGTCCAGCAAAACTTATTTCCATGAGGACTTTTTCAGAGGGATGAAGTAACATTAGCTATACAGGTTAGCATTATAAGACTTCCCAAGTGTAGATGAGAATAATGGCAACTCTGTGGTCCTAAGGATGAATATTGCTCTGGAATATGCATTTTACACTATATGAAAGAAATTAGGATCGATATAAGCTCACTTATCTTTGCCTTATTCCTCCTCATGTTGTTTTTGTCTAGATTGTCTCAGCCACTTGTTTTATTTTACTTAAATTTTAATTTCATCTTATTGTAAACCTCCATTCCTTCAGAAACAGGTCAAGAAACATGTCAATCTACCTAAGTGAATAACTAATATTAACAATTAAATAATAAATAGTACTGAATGAATATACGAATACAAGAATAAATAAAAATAAAATGTATTACTTCATCGATGGATTTCCTAGTAGATGGGGAAACGGTGAGAGGATATGAGCTTCAATAAGAAAAATGGTGCAAAATAAGGCAGAAGCAAATGCCCAAAACAAATCAACACATTCACAATTTTTCCAAGGACCTGTCATGTATATATTTTTTTCTTTTTTTAACCATTTGTGGCCCCTTTTTTTAACCATTTGTGGCCCCTTTCTTATATCTCATTTCTCTCTTTTGTAAGGCTTCTGTGTTAATTGACAGCATGTTCAGATATAAATCCATCACAGGAATGTGATGAAATTAGCCATTCAGACCCCTGATATTAAGAAATTCAAAGAAATGGATAGAGTATCCAACCAGTGAGGAATTAAAGAAGAAAGAAGAGAAAGAGAAGGAGAGAGAAACTAGCTGTAAAGTTGGGATGGGTCGGGGGTGGTGAAGAAAACCAATTATTCATTGAAGGTGCCAGAAGGAAAATTGATGGCATGAATCCATAGCTTCTCACCATAAAGGTGAATAATGACACAGACACTTAGATTGGGGAATGAGAAAAAAAAGGTGCATGCAAGGTTCTTCTATTTATATCTGATTAAGATATGAAAAGAAAATGAGAGACTGGATTACTAAAGAAAAATTCCAGACAGTTAAGCAATTTTAGGAATGATTCATTTTAAGATATGGCCATCAATTATTTATAAGGGTTAATAAATAGATTTATAAGCAAGAGGTACATGGAATCTAGAAATACATAAATGCTCTTCAAATTATTTACAGCTCTGACAAGTCATAACACATGAACTACTACCAAAAACACCATTTACTTGACTTTAAAATTTGCACCATAAACTATAAATGGACCAGTTATGGAGCATCAGCCATTTGTAATGTGCCATGCAATATTTAACATCAACTAAATGTGTTTTCACTAGCTGCTGACCACTTGGATTAATTTAATAAGCATGCCTAGTGCCTAATGATTTATTTGTGGGTAAATGATCATAACTATTTAATGGCCTTAATATTACAGATGTAATTCTGAAATAAAATATCATAACTTGGATTTAGTACATCCAGTTAAATAACAAGCATCGACATTTTTAAAAAATAATAAAAACAGTGGCCAGAAAAAGAAATTAAAGCACTTGCTAGTCATATGTCCCCATAGGTTTCCAGCTTCATATTGGCTTTATTTCTTTTTTTCCTTTCATTTAGGTCACCCATTAATTTTCTTTCTTCATTTGCACACCCTCTTCCATTTCCTGTACTATCTTTTGTTCTAATCCTCTAGTAATTCCCCAGTGAGCTCTCAGCTTCCAAAGGGCACTCTATTTCTATTAAGCATGGCAGTCAACAAGTGGAAATAGTCCTTGGTTGTCCTGCTTTCTGGGTGAATAGCAGAGTCCCTTTGCATCACCTCAAAGACTCTGATTCTCATGATCCTCAGTCTGGTGCTGAATTGTGCTTTTGCTCATCCACACACATCCCCTACC


Human Genome Map 11q24.2  (2100 bp sequence)
48A 

ACTCAGCAATGGGTAGCTATACTTGAGATAATAGGCGGGATTTTATGTGCAGCAATGTAGAGGATGCAGGGGCCCAGGATGGGCTGCCAGGTCTTCCAGAGAGTGCTAAGGTATCCACCAAGGATCATGAATGTGAACAAGATAATGAATCACTGTCTACTTACTCTTTTGGAAAAGCTTCCATATCTCTGCCAATTGAATCACACTATAACCAGTCCCAGGCAATTCAGGATGACAAGTTCCACTTCGAACAGTTCTGGGAGTCATCCTGAGGGTCCCTGTGTATAGACATAAAAAGTTCCATTTGTTCTTACACAGTGAAAATGACAGAACAAATATTATGGGGATTATGCCTGGGGAAAAAAAATCTGTCTCTGGATATTCCTGACACTATGGAGAGAAAATCAGCAAAATTTAGAATCTTGGATCTCTTCCACTCACACTAGGATGTTGTTTCTAGAAATCTCCCTGAAGTATGGTACTGACTCTTGGTGGTAAAAGTGGAGAGGCTTAGAACTGAAATCTGGTCAGTAGAAGACTGAGGGTTAAAAGTGGACGGTCAACCCATTGAATGAAGGCCTAGCAGGAAATAGAGAGACAAAAATACAGGCATTAAGGGAATAATAGCTGAATAGTAATAATAATACATTATGTCAACAGCGGTGACAAAGGAAACACTCAATGTATTTATAGAGCTAAATAAACGGCAGATCTAGGTCCTACGTTTTGACTCTGAACAACCTTCTCGGTTGGATTTTGCTTCTGCCTAAGGATTATTTTGGAAAGAGCTATTATTATCCGTGATTTATCACGCTGCACTGGGGGGAACTCATACTTTCCACGGAGACAATTACTGAATTCTCACTGGAGGCGCTTAAAGGAGCCAGGACCTGTTCTGAGGGTTCAGGTGGGAAAGGTGTGCCAGCAGGGGACTGCAGCCTGGCACCATGGGACGTGTGTGCTGTTGACCACTTCTGTGCCCAGATGCCTCAGGCGCTTTCTCATTAGATGCACCCTTCAATCTCCTGGTTATTGAAACAGGACTGGGGAGAGGAGTTCACATTTATGGTGAGCCCATGCAAGAAGACCCTCCGACAGGTGCCTGTCACCCCTGAGGAGTCACTGGTTGCAGCCCGTTCTGAAGTGTCATTGAGATAGAAACCAAGTCAAAGCCGTGGCCTAGAAAGAGAGTCTGGGCAGAATTCTGCAAGCAGATTCTTTATTTGAGTAAGTATTCCTTGAAGAAGCCCAGTTGTGCAGCTGTGTTTGGGTGGAGGTCATCAGAGGTTTAGAAAAAGAGAGAAGTCATGGTTAATATTAGAAAAGAACTCTGAGAATCTGGAGGAAGGAAAATGCATTACTAGTTCTAAGCAACAACTGTGGAAATAAACAATGATAAATACCGTATTAAATCTAAAGAGTTACGTTAATAGATAATAACAAGTAGGAGAGCTAATAGCTAGCCATTAATACAGGCCAATTTATTATTTAAAACATTTATTAAGATTTAACAATAGTCAAATAATTTTTTTGTGAAACAGTTATTAAACTGAATCTCTGCATACATTAATCAACTGATATTTATCATTCAGAATGTATCTCATTATATCCAAAAGGGTGTGTGTATAGGCTTCAAAAACAAACTGGAAGATTTAAAATGAACTGTAGTTCATTTTTGCAAAGTGTAGATGTGTAAAGATTATTATGTTTGCCAGCTGGGTAGCCAGACAGTGAAGTGGACTTGTCTAATTAGGAACAATCGCTGATAAATCAATTCTTTCCTTTTATAGGACAATTACAGTTTGTGTGTATATGTGATTGTGTTTTAAATTCTAATTCGATTTTGTGCATTGTTCTGTAACCAAGTTAATTCTTTGAAGCCTTTTTAAATGGTACAAATTTTCCATAAAATATAAATAGGTTTATTGCTGTTTTATCAGTCACGCAAATAATCCAAGATCCATCTATTCACATAATTCAGGCATTAACTGTGTATAATTACTCACATGAAGTCTTCAGTCTGGTTTACTATACGGAACCCCAAATATGACTTTAAATTGCTCGCCTCCTCTTTTCCTCTGTTATTTCTCTCCCTCTCTT


Human Genome Map 21q21 (2103 bp sequence)
49A 

TTGCATCATCTGACCTCTCTTCGAGTCCAAAGGACTGAGAACTAGAAGAACTACTGCTGTAAGTTCCAAAGTCCCAAGGCCCCTGAACTAAGATCTCCAATGTATGAGAGCAGGAAAAGATGGATATCCCAATTTAAGGAGAGAGAAAGAGAAAATTTGCCCTTCTTGTTCTATTTAGATCCTCAAGGAATTGGAATGCCCAACCACATTGGTGAGTGTGGGTCTTCTTTACTTATTCTACTGATTCAAATGTTAACAGATTCTGGAAACGTTCTCACAGATACACACAGAAATAATGTTTTGCCAGCTCTCTGGGCAGCTCTTAGCCCAGTCAAGTTTACATATAAGAATAATCAACCCAGCTTTTTATAATCATCTTAGTATTTAATCAAGGAAATGATATCAGCTATCTACTACCACAAAAATAATTTTAAAAATTGGCTTATAAAAGATGACTTAGTGGCCCTCAGCTGGGACAGCTTGTCACTATCCAACCAGCCTTTAAATCCTTCCAAGAAGCAATCCTGGCTTGTTCTCCTGAAAACTGGGTAGTTTTTCAAGAGATTGAGCAGAAGCATACAAGGCCTCCTGACACCTAGGTGTGAGATGGGCACACAACCACTCCTGTCAAATTCTATTGGCCAAAGCAAGTGACAAAGCCAATGCAGATTTAAGAGGTGGTGGAAAAAAACCCTAAAAATAGAAGTTGTTGAAAAGTCATATTTCAAAAGTCATTGGTATAAAGTAGTGAAAAATTTGACATTTTTGCAATCAAGCTTATCAAACAATATTATCCCAAAATATAACAATACACTCAGTTTGCACACTTGTTTACCTTTTGCAAAACAGGTAAGACAGTAGGACAAAGCAGGTGCTTTATGTTGTTTCAATCATTCAGGATTTGGACAGTTTGGATATTTTCTGTATCACTATAATTGATAAATACTCAGATGATTCTATAGTTAAGTTAGAATGGAAATTTTGGGTATAGTAACAAATACTACTTTAATTTAAACTTACATGTAAACAGTTTCCCTAAAGCAGTTAGAAGTGTGACCATAAAAGTGAAATGGTTTAAATACATGCATTTACATCTGTCCTAGAGTGATTAATGTAACTTTATTATAAAACTACTAATTTTGTGATTACATACACCCTTCCAAAGATACATTATACATTCCTATGTACACTCAAATATTATTTTTAAACTTCCATTCCAATCATTAAGTAGAAATGCATTTAAGAATCATGATTTTTTTAGAGTAAGTCTATAGGTGGTACTTTTATATTATAGATAACATTTCCTATACCCTTTCCACATAAACACAAGAACATTATGCTATAGATTGAAAATTCCTGTAAACACTAAGCAGAGCTTTTGTACATAAACTTGTAAAAACTCTACATAAATGTATTCAGAAATACATGCTATTAAAATATTTTATTGTATATTACTGTTTGGAAGTTTTCAGCTTAAATATTTTTATTTGATGATCAATAAGATCTAGTATTAAATGGTCTTATTTATTAACCATTAAGTTAAATACATGGAGAAATCCACTATGTCTTTTCCTCCAGCCTGTAAGTAACACAGGGTTGCATTTCTAATATTAACTAAGTTACATGTATTTTCCATTGAGAAGAGTGCTATCGAACTCATCCATGTTAAATCACTCTTATGTGGAAAAGGCTAACATATAAATAAAAAAACTAGAAAATTTAAAAAAGGATAAAGAAAGAAGAAAAATGAACAGAATTTAACAGCAGTGCAACAGTAGTCTCTTCCTACCTTTCCTGGGCATCTTCCAATTTTATGGTGGTCTGATAAGCTTTCCAAAACACTTTGCTCATTTCCAGCACTGGACATTTACACTCAAGACTGCAGACTCGAGGAGTCACACACTCAGCATCTTTTAGCTGTATGTTGTCAAGTTCAGACTACTCAAAGTGGCATGTCTTTAAATTAGAATGTGTCAAGTGGGTCTAGTAACTGCACCGAAATATTTTAATAGTCATATTAATCATTAATAAGTCAGGAAACATGTTTTTCTAATTTTCAGATCCCAATACACATGACTGATATGGTTTGCATCTGTGTCCCTC


Human Genome Map 21q21 (2100 bp sequence)
50A 

AAGGTTTGGAAAGCTTAAGTCAAAATGTGTTGTTCATAAATACGGTCTGAATAATTTGAACATTTTCTGTTAATGGTATTTGTTCAACTATAATGATATTTTCCAGCCAAGATATAATTGGCAATGTCAAAGTCACACACAGATGGGTAAAATGGCCAATGTCTCTGGAAAATCTTGATAATAACTTTTTAGTATCTCTGGTGCAAGGTCACTTAAATTCAGAAAATAGCACCCAAGGAAAAAATAGCCATATTCAAAAAAATAAGCTCCATATATTTAGATGTAGATATAAATTTGGGGTGATTTATTTCTTATTAGACACTAATATTTTTTAAAACAGAGAATGACAAATAAGGAAATTTTGCAGTTAACTATGTCCTAATGAAAAAGGGTAGTAGTTTTACAAGAAAGATATAATTCATCAAAAAGGCAGGGAAGCATTCAGACTAAACATTGAGTATGTTTGGAAATAATAAAAATTATTGTTTCTTTTACCAACATCAACAATCTTTTCAAATTAATTTATAAAACTGTCATCTCTGTTCACTAATTTTGAATTACTCATATTATTTTTAATTTTGAATACTTATAATATTACTTACTAATTTTTAATTAATTTTGATATACCTATATCACTGTTTTGAATTGATCTATGAATGATCTAGAAATGACTTTGCCTGTTTTTTTTTTTGACTCATGGGTATTTACTTTTCATTAGGTAATTTTAATGTATTGTTAACTAGAAAAATAAGATGAAGAAAAAAACATTTTAAATGCAAAATATAAATTTAAAGAACTTCAAAAGAATAAAATTTCAGTTTTATGTCTTTCAAGTAAATTTGCTGTTTTCAAAATTATTTTTTGTTACAAACCTATTTTATTTCAAAAAATATGCTATTGTTTTTAACCTATAATTTTTAAATATCTGACAGCATTGTAGGACTTAAAGCTATTAAATATATAAAGATATAATAGAACTTATTGGAAATATTCAAGGAAAAACTAACATATTCTTTAAAAACATTTTAATTTTTAAATTCTATGTTAATTGACTTTTTGATACATATTTTACTTTTCCTTCACTTCTTTTGTCAATTCTTAAAAATGTCTTTCTTCATAATTTTTGGCAATTAGTTTTTACACTTTAATAGCAAACATTGCCATAAAAGTGAAATTAAGCATTAATTAATTTTATGTCTGCAGGCAGAGTGATTTCCTTAGGGAATCAATTTAATAGAGAGAACTATGTTTGTACCTGGCAGGATATTCACAGAAATAAAATATTTATTGGCCATCTACTTTGTTTAAGACCTCTTAACAAACCATAACTTATTAAAGCATAAAGTAACATACATAGTAAATACTTTTAAAATCTGTAAACAACTAATTCCTTTCTTCTTGTGAAGTCTTGTTTAGATCATTAAAGTAATAGCAGATTTTCTCACAACAGGTTTGTGAATATTGTCTGTTTAACATGAAAACTATAAAAAAATTAAAGACAATTGATATATATTTATTCAACTATGTCAACTCAAAGATGATCTGCAATTGTTTTCTGAATAACTTATTAATAATGCTTAGGCCCCTTTGTTGAACATGCTTTTATTTGTGTAAATAAGAATTCATTTAAAAATACATTGTACAACTTCAACACATTGTGTGTCCCTGAAGGTACTCTGAGATTTTGCAGTTATAGTATAAATGAGACAAAACGGCAGAGAAAATATTCCCCATGTGTAATTCTTTCTACATTTATTTCCCACATCAATCTCACAAGTGTTTTTATTTCACACTGATTGATATCATTGAGCACATACCTCAATATCTATTATCACAAAAACTATCATTATCAACAAGGACTTTAAAAAATATCTAAACATTATTATCTGGGTAGCAACTCTATACTCCATTTTATCCATTAATTTTGTCTAATTAGTAAAGAAGTACTTATGGTAAAAACAAATTAAAAATAGTACAGAAAACATACTCCTGTATGCAATTATTACAAATATTTTATTTAGTTCCTATAAAGTATTTACATAGCTGAGATCACTATATAATATTATACTCATGTTACTTTATGTCCTAACTTTATATCA


Human Genome Map 11q23.2-q24.2  (2040 bp sequence)
51A 

CGGCTCTCCTGGCCTCGCGCTGCACATTCTCTCCTGGCGGCGGCGCCACCTGCAGTAGCGTTCGCCCGAACATGGCGACACGGAGCAGCAGGAGGGAGTCGCGACTCCCGTTCCTATTCACCCTGGTCGCACTGCTGCCGCCCGGAGCTCTCTGCGAAGTCTGGACGCAGAGGCTGCACGGCGGCAGCGCGCCCTTGCCCCAGGACCGGGGCTTCCTCGTGGTGCAGGGCGACCCGCGCGAGCTGCGGCTGTGGGCGCGCGGGGATGCCAGGGGGGCGAGCCGCGCGGACGAGAAGCCGCTCCGGAGGAAACGGAGCGCTGCCCTGCAGCCCGAGCCCATCAAGGTGTACGGACAGGTGAGCAGTTTTGCAACCCGCCTCCCTCCAGTTTTTTCCTCTCCCTGCACTTCCTCACCCCCGCATCCATCCGTTGCAGTCGCCTCCTAGGTGCAGGCACCACTGGGGACTTCCCGGCTTGCATTTGTTTTTTTCCTTCACGAGTACAACCGTCAGCACTTGAATCGCATTGATCTTTCCTTCTTCCTGTCGATTTAGTAAACGTATTCCAGGTAACTCGCCGGGTGCAGTGCGTATTACCCCAGGGTGTGTGCAGAGAGATGTAGTTTCCGGCAGGTATAGGAGGGGTGCAGCTTCATTTTACATCTGGATAAAAAACGGGCTTTCTTTAGTGTATCATCAGTTGGCAGTGGAGGCGAGCACCCTGCAGTTGCGGTACACTTACACAGAACAGCACGAGGTGGGGGTTTCCACACTTAGCATTATTAGCACAATAAAAGTGGGCAAACCTGAAAGCTTGTCGACTATCTCTGTACAGTCAGACAAGAGGTGTGTGTATGTGTGTGCGTGTGTAAAGGCTGAATTTTTAATTTTTAATTTTTGGCGAGCGTGTGAGATGCTCTCCATTCCTTCTTCCCCACCCTTCAAGATGCTGACTCTCCCACCCCCGTCAAGATAACTTTATTTTGGAGAGGAATACCCCTCATGGCACTTGGAGATTTGAAAGGACTGCAGGAAATTTGGTGGGCATTATTATTCTATAAGTGATTTATTTCTACCCAGGCAATAGGTTTATTAGATCATAAGTAACGTGAATTTCACTTTTATGGTCAGACTTACTGCGAGGAATTGCAGATGGAGTTTGTAGGTTAGGATCAGCACTGGCAAAATTAATTTGACCGTGTTATTGCCTCATGAGACTCCCAGTCCTGCAGTTAAGATTGACATCAGCAAAAGTATAAGGTCGGTGGGGGAGAAAAAGTAGGACCAGAGGAGGGGGTAAATACACTTGTTTTCTAGAGTCAAATTGTTCCTTTTGAAGTAGAAATTATTAATAAAAGATTACCCTGAGTTCTGCCTTTTCTCACTAATTTCACTTTAGCCATTTCTTCAGGAAATACAGAGTTAAATGTTCAACCCTTGGATCCAGGACGAACCTTGTAAACATATCACCCTATTGTGTCATTTTGTTGGTGAAGAAACTGAAGCGTGGAATGGTGAAGTGACTAGTCCAAGGTCATACCGGGAAGGTGGCCTGCTCTCTAGTTTTTGTCTGCATTGTCTCAGTGACCTTTGCTTGACTGCAGTCACCCTGTCTTTATGCAATGCTGCTGAAATACCTCCTTTCTAAAATAAAATAGATCTGGTATAAAGGGGGAAAGGATGGTGGTGACTGGGTGGGAGCGTTGGATTTCCCTCCACTATTGGTCCCTGGGCAAGAATGTGTGCCCCAGGGCATGTAACTAATGGTGGCCACAGGCTGCAGGAACCTGCATGCTCAGTTCCTCTTGGGCCCAGATCCTTGTCCCCCTGTCCCCACCCCATATGACAAATATGTGTATGAACAAAAAGAAGTCATCAAGGTCCTTGCTCTTAACAGCGACACCAGCATGGGGCTGATGGAGGGTGGGAGAAGGAGGAGGAGTGGCCCACTTCTTCATTGGGCCTCCGCAGTCAGCCCAGCTCTGCTGTGCTCTTGAATCAGCATTCTGGGAACTGGGAGTTGGGGGCTGGTGGGAGACAA


Human Genome Map 8p11.2  (2100 bp sequence)
52A 

ACAAAAGGCAAATTGGTGTCTCTGTCCTGGAGTCCTTACTCCTCATCTTGTGCTTAGACATGAAATTACACATCTCCAGCCTTGGGATTCCAGGACTTACACCAGTAGCATGCCTATGTTCTAAGGCCTTTGGCCTGGGACTGAGAATTACACCATCAGCTTTTCTGGTTCTAAGGCTTCTGGACCTGAACTGAGCCATGCTACCAGTATTTCAGGATGTTCAGCTTGCAGATAGCCTGTCGCGGAACTTCTCAGCCTCTAGAATCACATGAGTCAATTCCCCTAATAAATCTCCTTTTATCTATCTGAACATCTCTCTTCATCTCTCCATCCATCCACTCATGTGTCCATCCATCCATCCATCTATTGCTATCTATCTATCCATCCATGCATCCATCCATTCAACCATCCATCCACCCATCCATCCATCCCTGTGCCATCTATATCTATCTATCTATATATCTATCTATCCATGCATCCATCCATCCATCTATCCATCTATCCATCCATCACTATCTATCCATGCATCCACCCATCCACCCATCCATCCATCCATCCATCCATCCATCACTATCTATCCATCCATGCATGCATGCATCCATCCATCCATCCATCCATCCATCCATCCATCCATTTATCGCTATCTATCTATCCATCCATGTATCCATCCATCCATCTGTTCATCTATCACTGTCTATATATCTATGTATCTATCTATCCATCCATCCATGCATCCATCCATGCATCCATGCATCCATCTATCACTATCCATCCATCCATCCATCCATCCATTCATCCATCTATCTGTCTTCTACCTACCTACCTATCTAACTCTCTGGAGAACTCTGACTAATAAACTAGCTTTAAAACATGTTATTCTCTCTCTGCAATGTCTATTGCTTTATCTTCAGGAACATTCCACACATCCTGTAAGACTTCAGTTAAATTATCTCTCTGTTTCTTCTCCAATCATCCTCTGCCTTCCCTAGTCTCCTAACGTACTTTGTACATCTGTCACAAACCCCTCATCATATTTACTGTAATTTTTTTCCTACAGATTTGGATAGGAATTGAGCCATTTTTTTAATTTCACTTTTATGGTTGTTACAAATAAAAGAGCAAGCAGGCCCCTCACTGTAATTCACCTGTATTTGCATTTAACTTATTAACCAAGGCATACTATTTCAAATAATCTAATATAGTATTTCCTATTTAATAACCAAACATACAGAACAGTTCCAAGCACATGTAACCATGTGATACATTTTCCTCTTTGAATAATAAATATATTTCTTATAATTAATATGTGATAAAATTGCAATATTTTTAATCTCCTACATCCTTCTCTTTTAATCAGGTTTCCTTATCAACTGGTTCCTATCTCACGGGGTTGTTGCAGAGATGAGGAAAAAAAGTATTCTATTGGTTCATGCATCTCAAAATAGGCAGATTCTTTTCTCTGCTTCTTCCTTCATTGGCTCAGGTGTGGAGTGCTTCTCCCAATTATATGTGCCAGCCTTGGTATGTTCTCATTGCTGTACCACACTGCCTGAGACATCCAAGACCACATCTTCCTTTGGGGGCACATTGGACCTTTGTCATTGGCACTGGCAGGGAAGCTTTTATTTCACCAGGTCTAAGGCAATTCTTCCAAAAAAATCCCAAATAGTGAAAGAATTGATTTATTCTTCTAATATTTAAGCAAATGTAAAAAAAAAGTTACATTAGTTATGTTTTTTTCAGATTTTGGATCAGTGAGACTTCATTAAAACACTTTGAGGTTATAAAGCAAGTAATTTTTGTTTCCAGAAAAGTTAGTTTCCTTTGGCTGAAGGGACATCTCTATGCAGGCCAGATCAAGACAAAAATAACTTTTAAGAAGGGAAATGAGGGAATGGAGTTTGGAAAACATAAATCCCACAGCAAAGTACGTCACCAACAATAAGAGTCATCTCTTTCACAGAGGCCTTTCCTAGAAAAGCCCTGACAGACTAGGAGTCCAATCTTCGGCTCCCATAGCACCCATGCCTGCTTCCACTCTGGAGCTTACTACTTTGCGTTGAAATTAATTTTTACATGTCTATGGCTTCTATTACAAA
