# Supplementary material for: New Small Nuclear RNA Gene-Like Transcriptional Units as Sources of Regulatory Transcripts
Source: PLoS Genet. 2007 Feb 2;3(2):e1. doi: 10.1371/journal.pgen.0030001 (PMC1790723; doi:10.1371/journal.pgen.0030001)
Supplement: Supplementary file 3 [file pgen.0030001.st005.doc]

TABLE S5

Tr.Unit	Tr.Length(nt)	Hum.Gen.Map	Genomic Contig	Blast Human Genome	gi(gen.ident.)	BLAST Human ESTs	gi	e-value	
11A	344	14q22.1	-	H1 RNA	-	-	-	-	
12A	152	2p24.3	RP11-98I18	-	51460874	DB275493	83216976	2,00E-12	
14A	169	3p12	RP11-206J21	-	21206095	-	-	-	
17A	156	9q22-q31	RP11-349P17	GPR51 (intron 3) Sense	51467683	-	-	-	
19A	169	3p12.3	RP11-206J21	-	19774315	-	-	-	
20A	548	14q22.1	R218E20	-	11611180	-	-	-	
21A	324	8q24.1	RP1-316L14	CENPF(Intron 7,14,18)Antisense	22657510	AA737281	2767556	1,00E-31	
22A	201	6q16-q21	RP11-487F5	-	10045412	AA361955	2014276	6,00E-29	
23A	64	Xq21.3	RP13-258015	-	6855342	-	-	-	
24A	304	12q21	RP11-997P16	-	9957971	DA811538	81279558	6,00E-15	
27A	89	7q22	CTA-369K23	-	2341013	-	-	-	
29A	346	11p15	AJ400877	ASCL3 (intron 1) Sense 	8052236	BX645799	34480132	2,00E-36	
30A	142	Xp11.4	RP11-157D23	-	50582666	 DA496935	80536970	3,00E-05	
31A	230	12q21	RP11-743I10	-	21039699	AW303617	6713306	2,00E-19	
32A	128	17q21	MCK41	-	75875068	CA310957	24529055	9,00E-73	
33A	204	1q32.2	RP11-465N4	-	21622744	-	-	-	
34A	24	5q15	RP11-274E7	-	21281496	-	-	-	
35A	202	8p11.2	RP11-1147M13	-	28565756	BF995135	12401458	5,00E-21	
36A	117	3p12	RP11-564-P9	-	20334518	-	-	-	
37A	50	14q13	RP11-192K2	-	37550867	BM724961	19046292	4,00E-10	
38A	350	4p15.31	RP11-19D21	KCNIP4 (intron 1) Antisense	19807889	BF475563	11546390	8,00E-17	
39A	46	Xp11.3	RP5-1158H2	FLJ22843 (intron 10) Antisense	9581533	-	-	-	
40A	485	11p15	RP11-265F24	-	27413210	BG570298	13577951	4,00E-04	
41A	76	2q31	RP11-12N7	-	15668089	-	-	-	
42A	117	3p12.3	RP11-564P9	-	20334518	-	-	-	
43A	64	4q34.3	RP11-43303	-	18129587	-	-	-	
44A	223	4q13.3	RP11-401E5	-	18464317	-	-	-	
45A	76	4p14	RP11-1I10	APBB2 (intron 1) Antisense	18450176	-	-	-	
47A	43	2q22.1	RP11-745P9	-	16950374	-	-	-	
48A	402	11q24.2	RP11-168K9	-	32188045	-	-	-	
50A	154	21q21	AP001675	-	7768691	-	-	-	
51A	272	11q23.2	RP11-730K11	SORL1 (intron 1) Sense	14517581	BG698692	13966211	8,00E-04	
52A	140	8p11.2	RP11-1147M13	-	28565756	-	-	-
